# Organotypic human lung bud microarrays identify BMP-dependent SARS-CoV-2 infection in lung cells

**DOI:** 10.1016/j.stemcr.2023.03.015

**Published:** 2023-04-20

**Authors:** E.A. Rosado-Olivieri, B. Razooky, J. Le Pen, R. De Santis, D. Barrows, Z. Sabry, H.-H. Hoffmann, J. Park, T.S. Carroll, J.T. Poirier, C.M. Rice, A.H. Brivanlou

**Affiliations:** 1 Laboratory of Synthetic Embryology, the Rockefeller University, New York, NY, USA; 2 Laboratory of Virology and Infectious Diseases, the Rockefeller University, New York, NY, USA; 3 Bioinformatics Resource Center, the Rockefeller University, New York, NY, USA; 4 Laura and Isaac Perlmutter Cancer Center, New York University Grossman School of Medicine, NYU Langone Health, New York, NY, USA

**Keywords:** SARS-CoV-2, COVID-19, lung organoids, lung buds, fetal lung, endemic coronaviruses, micropatterned hESCs, BMP signaling, lung development, lung differentiation

## Abstract

Although lung disease is the primary clinical outcome in COVID-19 patients, how SARS-CoV-2 induces lung pathology remains elusive. Here we describe a high-throughput platform to generate self-organizing and commensurate human lung buds derived from hESCs cultured on micropatterned substrates. Lung buds resemble human fetal lungs and display proximodistal patterning of alveolar and airway tissue directed by KGF. These lung buds are susceptible to infection by SARS-CoV-2 and endemic coronaviruses and can be used to track cell type-specific cytopathic effects in hundreds of lung buds in parallel. Transcriptomic comparisons of infected lung buds and postmortem tissue of COVID-19 patients identified an induction of BMP signaling pathway. BMP activity renders lung cells more susceptible to SARS-CoV-2 infection and its pharmacological inhibition impairs infection by this virus. These data highlight the rapid and scalable access to disease-relevant tissue using lung buds that recapitulate key features of human lung morphogenesis and viral infection biology.

## Highlights

• Derivation of self-organizing and commensurate lung buds on microchips from hESCs
• Lung buds display *in vivo*-like proximo-distal patterning of lung tissue
• Parallelized quantitative analysis of SARS-CoV-2 viral life cycle in lung buds
• Identification of BMP signaling as a key mediator of SARS-CoV-2 infection via ACE2

## Teaser

Brivanlou and colleagues describe a high-throughput platform to generate self-organizing human lung buds from hESCs on microchips. Lung buds display *in vivo*-like proximo-distal patterning of lung tissue, and are amenable to high-throughput quantitative analysis of SARS-CoV-2 cell type-specific infection, transmission, and cytopathology. Transcriptomic comparisons with COVID-19 postmortem tissues identified the BMP signaling pathway as a key regulator of SARS-CoV-2 infection.

## Introduction

The emergence of SARS-CoV-2 in late 2019 sparked an explosive global pandemic of COVID-19 disease, with >665 million confirmed cases and >6.7 million deaths to date (Zhou et al., 2020; Zhu et al., 2020; World Health Organization, 2023). The rational design of COVID-19 therapies will require an understanding of the life cycle of the virus during infection in human cells. Key infection routes of SARS-CoV-2 involve the nasal passages, lung airways, and alveoli (Zhou et al., 2020; Zhu et al., 2020; Hou et al., 2020). In particular, the lung is the one of the most vulnerable target organs for SARS-CoV-2, as acute lung injury and pneumonia-associated complications are primary clinical outcomes in severe cases of COVID-19 (Zhou et al., 2020; Zhu et al., 2020; Hou et al., 2020). In this organ, airway multi-ciliated cells, alveolar type 2 (AT2) pneumocytes, and club cells are the primary targets of the virus (Hou et al., 2020; Muus et al., 2020; Sungnak et al., 2020; Ziegler et al., 2020). However, how SARS-CoV-2 induces local tissue damage and pathology in the lungs is not completely understood (Zhou et al., 2020; Zhu et al., 2020; Hou et al., 2020).

Current models of SARS-CoV-2 lung infection rely on *in vitro* cultures of human primary lung tissue (Hou et al., 2020; Katsura et al., 2020; Youk et al., 2020; Salahudeen et al., 2020; Tindle et al., 2021; Lamers et al., 2021), which remains challenging due to its irregular nature as well as its high inter-donor phenotypic and genetic variability. These features are of particular importance as genetic heterogeneity plays a large role in SARS-CoV-2 replication and outcome (Williamson et al., 2020; Zhang et al., 2020). To circumvent these limitations, protocols have been developed to differentiate human embryonic stem cells (hESCs) into lung airway and alveolar cells as an alternate source of tissue to study lung biology and disease (Jacob et al., 2017; McCauley et al., 2017; Dye et al., 2015; Miller et al., 2019; Green et al., 2011; Huang et al. 2014, 2015; Chen et al., 2017). hESCs-derived lung tissues have been used to study cellular responses upon SARS-CoV-2 infection and to identify small molecules that halt SARS-CoV-2 infection (Han et al., 2020; Huang et al., 2020). This highlights the potential of using hESC-based platforms for the study of SARS-CoV-2-associated lung pathology and for high-throughput identification of therapeutics for COVID-19. A caveat of stem cell-based models of lung tissue is that, despite providing an inexhaustible supply of human lung cells, they lack the controlled tissue organization observed in developing and adult lung tissue such as the coordinated segregation of alveolar and airway tissues (Morrisey and Hogan, 2010; Nikolić et al., 2018). Moreover, current protocols take 1 to 3 months to differentiate lung cells from hESCs, underscoring a need to develop fast and scalable platforms to generate these cells *in vitro*. Finally, many of these organoid systems suffer from inter-organoid variability and reproducibility (Nikolić et al., 2018), which limits their compatibility with chemical and genetic screening at a high-throughput scale. In this article, we describe a novel lung organoid technology platform to generate thousands of nearly identical lung buds from human embryonic stem cells (hESCs). The process allows for rapid and scalable access to lung tissue, resembling human lungs in cell type and tissue complexity. The reproducible and scalable nature of these lung buds allow quantitative analysis of infection by SARS-CoV-2 and endemic coronaviruses, but also interrogate cell type-specific cytopathogenic and intercellular transmission events.

## Results

### Reconstituting human lung development on confined geometries

**Figure 1:**
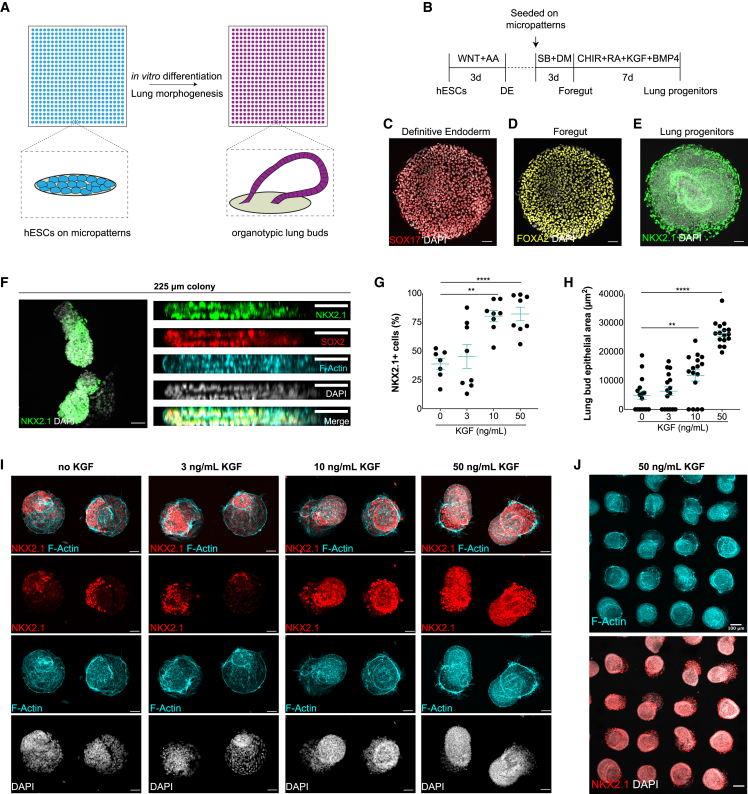
Generation of self-organized epithelial lung buds on confined geometries (A) Experimental paradigm to generate self-organized lung tissue from hESCs on micropatterned colonies. (B) Protocol for the generation of lung buds on confined geometries in micropatterns. (C–E) Generation of SOX17+ endoderm cells (C), FOXA2+ anterior endoderm cells (D), and NKX2.1+ multipotent lung progenitors (E) at the end of definitive endoderm (DE), foregut, and lung progenitor induction stages, respectively. (F) Top and side views of 3D epithelial buds containing NKX2.1+ multipotent lung progenitors in 225-μm colonies. (G) Proportion of NKX2.1+ progenitor cells at increasing doses of KGF (N = 8 independent experiments, data represent mean values ± SD). (H) Quantification of lung bud epithelial area in micropattern colonies at varying doses of KGF (N = 14 independent experiments, data represent mean values ± SD). (I) Micropattern colonies containing NKX2.1+ epithelial buds at increasing doses of KGF. (J) Low-magnification images of epithelial buds containing NK×2.1+ lung progenitors grown on confined geometries of 225-μm diameter at high doses of KGF (50 ng/mL) (^∗∗^p < 0.01,^∗∗∗^p < 0.001, ^∗∗∗∗^p < 0.0001, Dunnett’s multiple comparison test; scale bar, 50 μm).

Micropattern cell culture technology allows the robust and scalable generation of human organotypic tissues that model the *in vivo* embryonic counterparts in glass chips. hESCs grown in confined geometry self-organize to generate patterns of differentiated cells, modeling the early development of human organs (Warmflash et al., 2014; Haremaki et al., 2019). To overcome current limitations of existing stem cell-based models of lung development and respiratory infections, we sought to develop a micropattern-based platform to generate organotypic embryonic tissues that model fetal human lungs (Figure 1A). The generation of self-organized lung tissues relies on the stepwise modulation of signaling pathways that direct lung development *in vivo* (Figure 1B) (Jacob et al., 2017; McCauley et al., 2017; Dye et al., 2015; Miller et al., 2019; Green et al., 2011; Huang et al., 2014, 2015; Chen et al., 2017). As lung progenitor cells are derived from anterior endodermal progenitors in the embryo, we first induce hESCs to differentiate into SOX17+ definitive endoderm in standard monolayer cultures, by applying WNT and ACTIVIN stimulation for 3 days (Figures 1C, S1A, and S1B). Endodermal cells were then seeded on micropatterned substrates and exposed to the transforming growth factor β inhibitor SB431542 (SB) and BMP inhibitor Dorsomorphin (DM) for another 3 days to induce FOXA2+ anterior endoderm (foregut; Figure 1D) (Green et al., 2011; Huang et al., 2014, 2015). Finally, cells were exposed to WNT-activation, KGF, BMP4, and retinoic acid (RA) stimulation for 7 days, to promote the differentiation of NKX2.1+ multipotent lung progenitors (Figure 1E). These progenitors give rise to both airway and alveolar cell types (Morrisey and Hogan, 2010; Nikolić et al., 2017, 2018; Rawlins et al., 2009; Zepp and Morrisey, 2019). We find that compared with standard monolayer differentiation protocols where KGF is dispensable (Jacob et al., 2017; McCauley et al., 2017), a combined induction by KGF and BMP4 together, rather than each alone, led to a significant increase in the proportion of NKX2.1+ cells on lung progenitor micropatterned colonies (Figures S1C–S1F). Specification of lung progenitor fate is dependent on the levels of KGF and BMP4 signaling, as we detected a dose-dependent increase in the proportion of NKX2.1+ progenitors in micropatterned cultures (Figures 1G–1J, S1C–S1F, and S1H–S1J). This platform allows us to generate thousands of self-organizing lung progenitor colonies of defined sizes in a single micropattern chip (Figures S1G and S1H).

During early human development *in vivo*, fetal lung progenitors arise in epithelial buds that form from an outpouching of the anterior endoderm, co-expressing NKX2.1 and SOX2, around week 4 of gestation (Morrisey and Hogan, 2010; Nikolić et al., 2017, 2018; Rawlins et al., 2009; Zepp and Morrisey, 2019). Later, NKX2.1 expression becomes highly enriched in the alveoli, while SOX2 expression specifically demarcates the airway (Morrisey and Hogan, 2010; Nikolić et al., 2017, 2018; Rawlins et al., 2009; Zepp and Morrisey, 2019). Interestingly, we find that upon induction, multipotent lung progenitors self-organize into three-dimensional (3D) epithelial chords containing NKX2.1+/SOX2+ progenitors (Figures 1F and S1G), reminiscent of fetal lung buds *in vivo*. The number of individual buds increased with colony size and was restricted to a single individual bud in 225-μm-diameter colonies (Figures 1F and S1G). We find a dose-dependent increase in the epithelial area of lung buds upon KGF modulation (Figures 1G–1I and S1H). High doses of KGF (50 ng/mL) robustly induce epithelial lung buds that are commensurate in size and morphology (Figures 1J and S1H). This model highlights the self-organizing capabilities of stem cell-derived lung progenitors on confined circular geometries with *in vivo*-like morphogenetic features.

### Proximodistal patterning of fetal-like human lung buds

**Figure 2:**
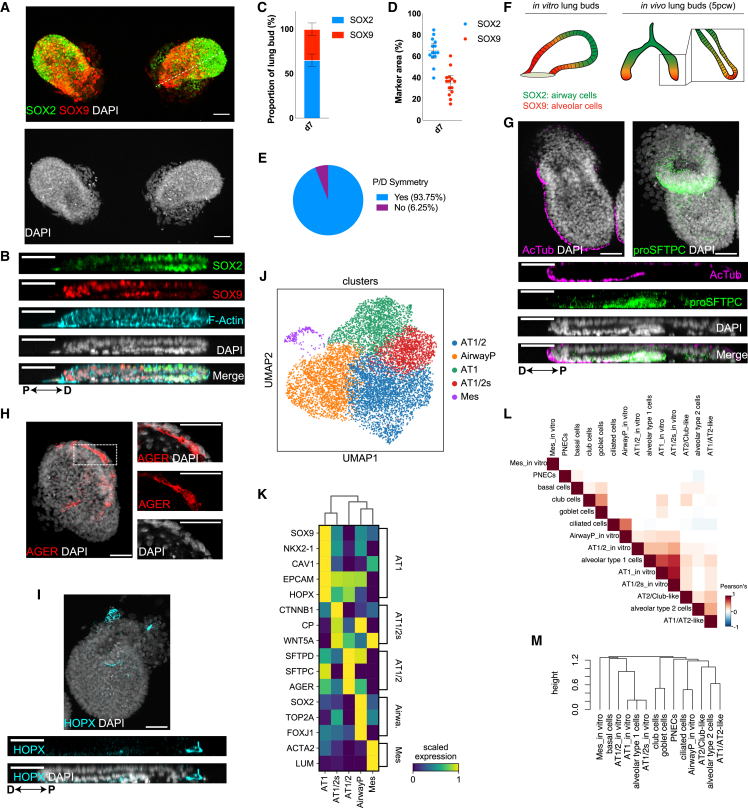
Proximodistal coordination of airway and alveolar tissue differentiation in human lung buds (A and B) Top (A) and side (B) views of lung buds with proximodistal segregation of SOX2+ airway and SOX9+ alveolar progenitors. (C) Proportion (C) and percentage area (D) of SOX9 and SOX2 expression domains in lung buds (N = 3 independent experiments, data represent mean values ± SD). (E) Percentages of lung buds displaying proximodistal organization of airway and alveolar tissue (N = 3 independent experiments). (F) Diagram of lung buds on micropatterns *in vitro* (C) or in human embryos at post-conception week (pcw) 5 *in vivo*. (G–I) Top view and side view of lung buds and identification of multi-ciliated (AcTub+, G), type 2 pneumocytes (proSFTPC+, G), type ½ pneumocytes (HOPX+, I), and type 1 pneumocytes (AGER+, H). (J) UMAP plot and identification of five major cell clusters in lung buds by single-cell RNA-sequencing. (K) Heatmap of scaled gene expression levels in each of the identified cell types of cell type-specific lung markers of alveolar AT1 (CAV1+), AT1/2 (AGER+,SFTPD+), and AT1/2s (CTNNB1+), airway progenitors (SOX2+, FOXJ1+, TOP2A+) and mesenchymal cells (ACTA2+, LUM+). (L and M) Cluster-level gene expression Pearson’s correlation analysis and hierarchical clustering of cell types identified in *in vitro*-derived lung buds and in adult lung tissue. Scale bars, 50 μm. D, distal; Mes, mesenchyme; P, proximal; proSFTPC, pro-surfactant protein C; SOX2+ Prog, SOX2+ progenitors; SOX9+ Prog, SOX9+ progenitors.

Upon induction of the lung primordium, epithelial buds are further patterned along their proximodistal axis, which leads to a coordinated segregation of proximal SOX2+ airway and distal NKX2.1+/SOX9+ alveolar progenitors (Figures 2A, 2B, and 2F) (Morrisey and Hogan, 2010; Nikolić et al., 2017, 2018; Rawlins et al., 2009; Zepp and Morrisey, 2019). This initial patterning event is critical for the coordination of region-specific tissue morphogenesis and the differentiation of airway and alveolar cells that will ensue. These features are not faithfully and robustly recapitulated in current lung organoid protocols in a reproducible manner (Nikolić et al., 2018). Strikingly, lung progenitors grown on small, confined geometries (225-μm diameter) display proximodistal coordination of progenitor differentiation with SOX9+ alveolar-like and SOX2+ airway-like cells located in non-overlapping tissue domains (Figures 2A and 2B), modeling human embryonic fetal lung bud development around week 5 of gestation (Morrisey and Hogan, 2010; Nikolić et al., 2018). SOX9+ alveolar-like progenitor cells are positioned proximal to the micropattern surface, whereas SOX2+ airway-like progenitor cells are located more distally forming a continuous 3D epithelial structure (Figures 2A and 2B). The proximodistal patterning and *in vivo*-like segregation of airway and alveolar progenitors was observed in 94% of micropatterned colonies with highly reproducible marker expression domains (Figures 2C–2E). These human lung buds also display early hallmarks of cellular differentiation and express markers of airway multi-ciliated cells (AcTub+; Figure 2G) and basal stem cells (Figure S1L), as well as alveolar type 1 (AGER+, HOPX+, Figure 2H-1) and type 2 pneumocytes (proSFTPC+, HOPX+, Figures 2G and 2I). Type 1 and type 2-like pneumocytes are located proximal to the micropattern surface and airway multi-ciliated cells are in the EPCAM+ epithelium forming in the distal tip of the lung bud (Figures 2G–2I and S1K–S1M). In 500-μm lung progenitor colonies, we also detected airway basal stem cells (P63+) and mucus-producing goblet cells (MUC5AC+) in epithelial cords that were less organized compared with 225-μm micropatterns, presumably due to size-dependent tissue morphogenesis imposed by geometric confinement (Figures S1N–S1R). Thus, lung progenitor cells cultured on confined geometries self-organize into fetal-like human lung buds with proximal-distal coordination of alveolar and airway tissue differentiation.

### Molecular signature of standardized human lungs

To further characterize cell types present in self-organizing human organotypic lungs, we performed single-cell RNA-sequencing (scRNA-seq) analysis at day 7 of lung differentiation in 225-μm circular micropattern colonies. Using cell type-specific markers of lung cells, previously identified in scRNA-seq analysis of primary human lungs (Travaglini et al., 2020; Wang et al., 2020), five major cell subpopulations were identified. These consist of (1) SOX2+ airway progenitors (AirwayP), (2) alveolar progenitor cells (AT1/2), (3) cycling alveolar progenitor cells (AT1/2s), (4) early type 1 pneumocytes (AT1), and (5) mesenchymal (Mes) cells (Figures 2J, 2K, and S2A–S2E). SOX2+ airway cycling progenitors can be identified based on the enriched expression of SOX2 and FOXJ1 and TOP2A, a cell cycle marker (Figures 2K, S2D, and S2E). Early progenitors AT1/2 cells co-expressed the AT1 marker AGER as well as surfactant proteins SFTPD, SFTPC, and SFTPB (Figures 2K, S2E, S2G, and S2H). AT1 cells display high levels of SOX9 as well as the AT1 markers CAV1 and HOPX (Figures 2K, S2G, and S2H). AT1/2s display high levels of the canonical AT2 marker ETV5, as well as CTNNB1, CP, WNT5A, and TCF7L2 (Figures 2K, S2G, and S2H), which are marker genes of progenitor-like alveolar cells (Travaglini et al., 2020). Compared with other alveolar cell subpopulations, AT1/2s cells express high levels of the cell cycle marker TOP2A, suggesting that these cells correspond to stem cell-like cycling alveolar cells (Figures S2G and S2H). The identity of mesenchymal-like cells (Mes) is based on the expression of lumican (LUM), collagen (COL3A1, COL1A2), and ACTA2, a marker of lung mesenchyme (Figures 2K, S2D, and S2E) (Danopoulos et al., 2018, 2020).

We hypothesize that alveolar cells present in lung buds correspond to a fetal immature state. Although the expression of key markers of alveolar cells, such as SFTPC, SFTPB, HOPX, PDPN, AGER, SPOCL2, and ETV5, is detected in these cells (Figures S2F–S2H), markers of mature alveolar cells, such as ABCA3 (critical for lamellar body formation), LAMP3, and NAPSA, are not expressed at levels detected by scRNA-seq. Airway cells likely correspond to multi-ciliated cells as they expressed key markers of this cell type including SOX2, FOXJ1, and TPPP3 (Figures 2K and S2D–S2F). We did not detect high expression levels of intestinal (CDX2), liver (HHEX), or thyroid (PAX8) markers in lung buds based on our scRNA-seq analysis, suggesting that lung bud cells are not hepatic, thyroid, or intestinal-like (Figure S2F).

To ascertain whether lung buds display conserved gene expression with *in vivo* counterparts, we performed canonical correlation analysis and data integration (Stuart et al., 2019) to align our dataset to a published dataset of fetal (30 weeks), juvenile (3 years), and adult human lungs (30 years) (Wang et al., 2020). Cluster-level correlation analysis, based on the average expression value for each gene in each cell type, unveiled high correlation between alveolar-like cells AT1/2, AT1/2s, and AT1 in *in vitro* lung buds with AT1 of primary human lung tissue (Figure 2L), with the highest correlation observed between AT1 cells of primary human lung tissue and lung buds (Figure 2L). These cells also displayed positive correlation with AT2/Club-like and AT1/AT2-like cells *in vivo,* possibly due to their progenitor-like phenotypic signature (Wang et al., 2020). Moreover, AirwayP cells in lung buds displayed high correlation with ciliated cells in primary human lung tissue (Figure 2L). We observed positive correlation (correlation coefficient >0.05) with primary alveolar and airway across all developmental stages analyzed with no clear stage-specific correlation signature (Figures S2I–S2K). Hierarchical clustering based on cluster-level correlations between *in vitro* and *in vivo* datasets further identified two clusters containing both *in vitro*-derived and *in vivo* lung tissue: one with AT1 and AT1/2 alveolar cells and one with airway ciliated cells (Figure 2M). Thus, lung buds display conserved cell types and convergent gene expression with human lung tissue *in vivo*.

### Tracking viral infection and transmission in human lung buds

As these human lung buds contain cellular targets of SARS-CoV-2 and endemic coronaviruses, we sought to determine whether we can track SARS-CoV-2 infection and pathology in these tissues. We hypothesized that organotypic human lung buds could be infected by various coronaviruses as we detected the expression of coronavirus receptors ACE2 and ANPEP as well as associated proteases FURIN and TMPRSS2 in multiple cell types (Figures S3A–S3C). To test lung bud susceptibility, we infected the lung buds at day 7 with the betacoronavirus HCoV-OC43 and alphacoronaviruses, HCoV-229E and HCoV-NL63. High levels of infection were observed for the endemic coronaviruses HCoV-229E, HCoV-OC43, and, to a lower extent, HCoV-NL63, suggesting that *in vitro* stem cell-derived human lung buds are a tractable model to study respiratory infection by human coronaviruses (Figures S3D–S3G).

**Figure 3:**
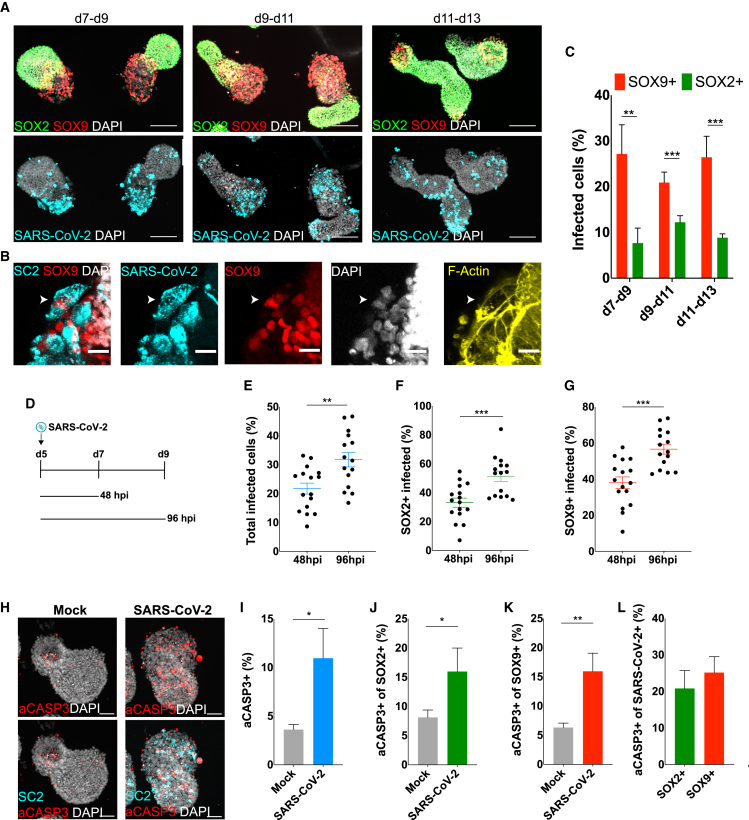
SARS-CoV-2 infect alveolar and airway tissue in human lung buds (A) Lung buds infected with SARS-CoV-2 at day 7, 9, or 11 of lung bud formation and collected 48 h post-infection (hpi) (scale bar, 100 μm). (B) SARS-CoV-2 infection in SOX9+ alveolar cells (scale bar, 50 μm). (C) Percentage of infected cells in SOX9+ alveolar and SOX2+ airway cells (N = 9 independent experiments, data represent mean values ± SD). (D) Experimental scheme to track SARS-CoV-2 transmission in lung buds. (E–G) Percentage of infected cells of total, SOX2+ and SOX9+ cells 48 hpi and 96 hpi (N = 9 independent experiments, data represent mean values ± SD). (H–L) Detection and quantification of active CASP3+ apoptotic cells in mock and SARS-CoV-2 infected lung buds (scale bar, 50 μm; N = 9 independent experiments, data represent mean values ± SD). ^∗^p < 0.05, ^∗∗^p < 0.01,^∗∗∗^p < 0.001, ^∗∗∗∗^p < 0.0001, Dunnett’s multiple comparison test. aCASP3, active Caspase-3; D, distal; P, proximal; SC2: SARS-CoV-2.

To determine if this platform recapitulates COVID-19 pathology, we infected lung buds at different stages of lung bud formation with a patient-derived isolate of SARS-CoV-2 (USA-WA1/2020; Figures 3A–3C and S3H–S3J). We detected viral infection in both SOX2+ airway and SOX9+ alveolar cells (Figures 3A–3C and S3H–S3J). In contrast to experiments with primary lung tissue cultured *in vitro* (Hou et al., 2020), we identified an increased susceptibility to infection in alveolar cells compared with airway cells (Figures 3C and S3H–S3J). Consistent with these results, we detected higher expression of the receptor ACE2 as well as proteases TMPRSS2 and FURIN, which regulate SARS-CoV-2 entry into target cell types (Shang et al., 2020; Stuart et al., 2019; Walls et al., 2020), in alveolar cell types compared with airway cells (Figures S3A–S3C).

To assess whether the virus could spread to uninfected lung bud cells, infection was tracked for 48 and 96 h post-inoculation (Figure 3D). Notably, there was a marked increase in the number of infected cells over time in a cell type-independent manner (Figures 3E–3G), suggestive of viral spread in human lung buds. We also sought to determine whether lung buds recapitulate cytopathic effects upon infection. Apoptosis is recognized as an important host antiviral defense mechanism that controls viral infection and regulates inflammatory responses (Ren et al., 2020). Upon infection by SARS-CoV-2, cellular apoptosis is induced by both host antiviral response and accessory viral proteins such as ORF3a (Ren et al., 2020). Infected cells displayed high, yet similar, levels of apoptosis in both alveolar and airway tissues compared with mock lung buds mimicking virus-induced pathology (Figures 3H–3L). This platform recapitulates the complete viral life cycle while also enabling tracking of cell type-dependent susceptibilities to infection, intercellular transmission, and cytopathic effects in lung buds.

**Figure 4:**
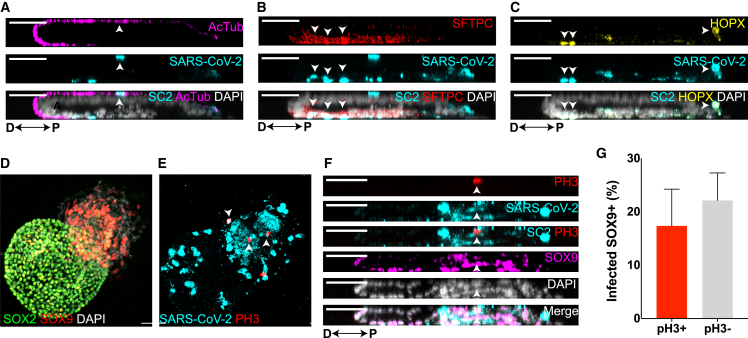
Cycling alveolar stem cells are targets of SARS-CoV-2 (A–C) Detection of SARS-CoV-2 infection in multi-ciliated (AcTub+; A), type ½ pneumocytes (proSFTPC+, HOPX+; B and C). Arrows denote infected cells expressing cell type-specific markers (scale bar, 50 μm). (D–G) Detection and quantification of SARS-CoV-2-infected pH3+ cycling SOX9+ alveolar cells. Arrows denote infected pH3+ cycling cells (N = 9 independent experiments, data represent mean values ± SD). Scale bar, 50 μm. D, distal; P, proximal; PH3: phospho-Histone H3; SC2: SARS-CoV-2.

Intriguingly, inspection of SARS-CoV-2-infected alveolar and airway tissue identified SFPTC+ alveolar cells and to a lower extent AcTub+ multi-ciliated cells as targets of SARS-CoV-2 in lung buds (Figures 4A and 4B), corroborating *in vivo* analyses of infected tissues from patients (Hou et al., 2020). The data also identify type 1/2 pneumocytes (HOPX+) as additional targets of SARS-CoV-2 (Figure 4C), consistent with their high levels of expression of ACE2 receptor and SARS-CoV-2-activating proteases (Figures S3A–S3C), as in adult human tissue (Ziegler et al., 2020). Surprisingly, SARS-CoV-2 infection was also observed in phospho-Histone3 (pH3)-positive cycling SOX9+ alveolar progenitor cells, which correspond to cycling AT1/2s (Figures 4D–4F). These cells also display high expression of ACE2 and virus-activating proteases (Figures S3A–S3C). However, there were no significant differences in the levels of infection between dividing and non-dividing SOX9+ alveolar cells (Figure 4G), suggesting that cycling stem cell-like alveolar cells are highly susceptible to infection at levels similar to non-dividing alveolar cell types.

### Human lung buds for comparative therapeutic screens

**Figure 5:**
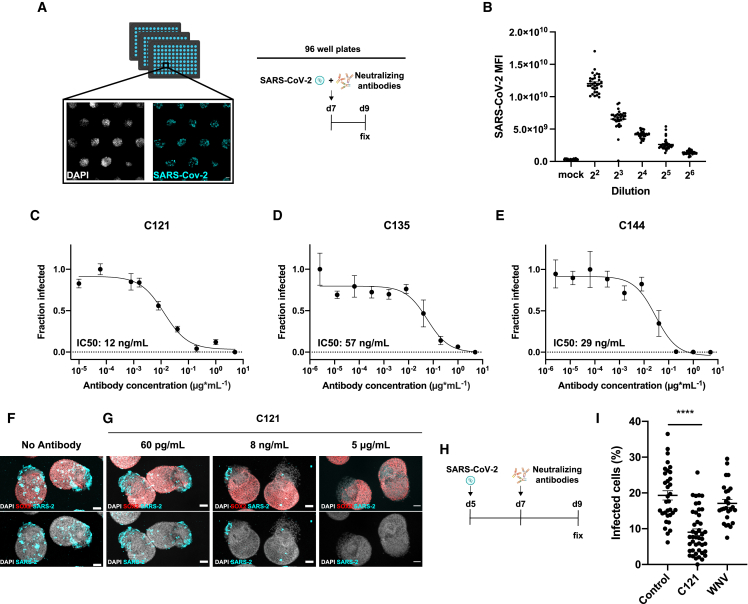
Evaluation of antibodies that neutralize SARS-CoV-2 infection (A) Experimental scheme for the high-throughput-based testing of SARS-CoV-2-neutralizing antibodies in 96-well plates. (B) Quantification of dose-dependent SARS-CoV-2 infection in the high-throughput micropattern lung bud platform (N = 16 independent experiments, data represent mean values ± SD). (C–E) Neutralization curves of SARS-CoV-2 infection in lung buds at increasing doses of antibodies. Data normalized to maximum (1) and minimum (0) infection levels (N = 3 independent experiments, data represent mean values ± SD). (F and G) Representative images of lungs infected with SARS-CoV-2 and treated with varying doses of C121 human antibody (scale bar, 50 μm). (H) Experimental scheme to test the effect on neutralizing antibodies on intercellular transmission. Lung buds were infected with SARS-CoV-2 at day 5 of lung bud formation and treated with neutralizing antibodies 48 h later. (I) Proportion of infected cells in human lungs treated with C121 or a West Nile virus (WNV)-specific antibody from day 7 to day 9 (N = 3 independent experiments, data represent mean values ± SD). ^∗∗∗∗^p < 0.0001, Dunnett’s multiple comparison test. MFI, mean fluorescence intensity.

As this platform mimics *in vivo* features of lung tissue and is scalable, we next tested for the ability to screen therapeutics that were shown to limit SARS-CoV-2 infection through binding assays and in cell lines (Robbiani et al., 2020). We developed a 96- and 384-well plate-based assay for high-throughput-based characterization and screening of COVID-19 therapeutics (Figure 5A). Using this high-throughput assay, we were able to assess dose-dependent infection in a highly robust and quantitative manner (Figure 5B). Human antibodies isolated from convalescent plasma of patients (Robbiani et al., 2020) were tested for their efficiency in neutralizing SARS-CoV-2 and preventing infection (Figure 5A). Each neutralizing antibody (nAb) was tested at a range of dilutions (Figures 5C–5E). Each nAb potently inhibited SARS-CoV-2 infection (Figures 5C–5E) at half maximal inhibitory concentrations similar to those found for cell lines (Robbiani et al., 2020). As expected, the nAbs broadly inhibit SARS-CoV-2 infection, rather than in a cell type-specific manner (Figures 5F and 5G). Although the initial assay shows that nAbs can inhibit infection when incubated with the virus prior to infection, we next wanted to mimic more pragmatic therapeutic approaches, whereby the nAb is administered once the patient has already been infected by the virus to inhibit viral spread (Figure 5H). We find that nAbs can inhibit SARS-CoV-2 spread throughout the organoid, when compared with an nAb control (Goo et al., 2019), highlighting the utility of this system to mimic *in vivo* situations (Figure 5I) and to test candidate COVID-19 therapeutics in a high-throughput manner.

### SARS-CoV-2 infection-associated lung-specific gene expression signatures

**Figure 6:**
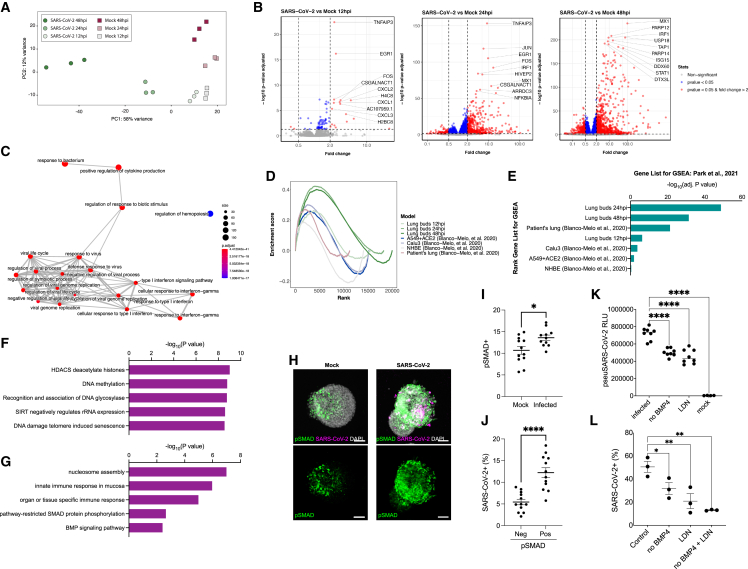
BMP signaling is induced in infected lung tissue and regulates SARS-CoV-2 infection (A) Volcano plots of DEG genes of SARS-CoV-2 infection compared with mock lung buds 12, 24, and 48 hpi. (B) PCA analysis of gene expression of SARS-CoV-2-infected and mock lung buds 12, 24, and 48 hpi. (C) Network plot of the top 20 enriched gene ontology terms based on the similarity of the genes they contain. Gene ontology terms were identified from GSEA analysis of genes ranked based on their contribution to PC1. (D and E) GSEA enrichment scores (D) and adjusted p values for the enrichment (E) of patient-associated COVID-19 gene expression signatures (Park et al., 2022) in lung buds and other *in vitro* models. (F and G) Overrepresentation analysis of lung-specific SARS-CoV-2-associated upregulated genes from 9D) based on gene ontology biological process (F) or Molecular Signatures Database (MSigDB) C2 categories (G). (H) pSMAD1/5 expression in SARS-CoV-2-infected and mock lung buds 48 hpi. (I) Proportion of pSMAD1/5+ cells in SARS-CoV-2-infected and mock lung buds 48 hpi (N = 9 independent experiments, data represent mean values ± SD). Scale bar, 100 μm. (J) Proportion of SARS-CoV-2-infected cells in pSMAD-negative or positive cells 48 hpi (N = 9 independent experiments, data represent mean values ± SD). (K) Quantification of viral entry by pseudotyped SARS-CoV-2 upon modulation of BMP pathway 48 hpi (N = 9 independent experiments, data represent mean values ± SD). (L) Proportion of SARS-CoV-2+ infected cells upon BMP pathway modulation 48 hpi (N = 3 independent experiments, data represent mean values ± SD). ^∗^p < 0.05, ^∗∗^p < 0.01, ^∗∗∗∗^p < 0.0001, Dunnett’s multiple comparison test. RLU, relative luciferase units.

To further identify cellular responses to infection, we explored gene expression programs associated with SARS-CoV-2 infection. Time course RNA-seq analysis of infected lung buds was performed at 12, 24, and 48 hours post-infection (hpi) (Figures 6A and 6B). Principal-component analysis (PCA) of RNA-seq data of infected lung buds collected at multiple stages post-infection showed a time-dependent association of gene expression along the first principal component (PC1) with 58% of the variance explained (Figure 6A). For each time point, differentially expressed genes (DEGs) were compared with mock stage-matched tissue (Figure 6B). As observed in patient samples, there was marked increase in genes representative of pro-inflammatory and type I interferon response pathways, such as TNFAIP3, NFKBIA, STAT1, and IRF1, among others, as early as 12 hpi, and increasing at 48 hpi (Figure 6B). Consistently, further gene set enrichment analysis (GSEA) of genes identified an enrichment of gene ontology terms related to regulation of viral process, cellular responses to viral infection, as well as cytokine production and cellular responses to type 1 interferons (Figure 6C).

Since a large fraction of the gene expression changes observed have been associated with responses to viral infection, we sought to identify SARS-CoV-2-specific gene expression programs. To do that, we collected RNA-seq data of lung buds infected with endemic coronaviruses OC43 or 229E (Figures S4A–S4C). We observed a divergent antiviral gene expression response to infection by OC43 and 229E compared with SARS-CoV-2 based on PCA (Figure S4A). Among DEG genes (log2 fold change >2 and adjusted p value <0.05), we found that 1,678 genes are specific to SARS-CoV-2 infection (Figures S4B and S4C). Our analysis highlights both divergent and convergent cellular responses to infection by coronaviruses in lung tissues.

Organotypic self-organizing lung buds provide a paradigm to model physiological responses to SARS-CoV-2 infection. GSEA analysis of our dataset with gene sets previously associated with SARS-CoV-2 infection in cancer cell lines, bronchial epithelial cells, and tissues from COVID-19 patients (Blanco-Melo et al., 2020), highlighted convergent gene expression programs across these models based on enrichment analysis (Figure S4D). To further identify gene expression responses to SARS-CoV-2 infection that are specific to infected lung tissue, we compared our dataset with a published dataset of postmortem tissues from COVID-19 patients (Park et al., 2022). DEGs (adjusted p value <0.05) were identified for SARS-CoV-2-infected lung buds (this study), lung postmortem tissue (Park et al., 2022), and the cancer cell lines A549 and Calu3 (Blanco-Melo et al., 2020). GSEA rank analysis of DEGs (adjusted p value <0.05) in COVID-19 lung postmortem tissue identified a significant enrichment of COVID-19-associated gene signatures in infected lung buds (Figures 6D and 6E). We further detected an increased enrichment of the patient-associated gene signatures in lung buds compared with other *in vitro* models, including cancer cell lines A549 and Calu3 (Figures 6D and 6E). Moreover, we identified 145 genes that were differentially expressed in infected lung buds and postmortem lung tissue, but not in the cell lines (Figure S4E), suggesting that these genes are lung-specific. Among those genes, we found an enrichment of pathways related to DNA methylation, DNA damage-induced senescence, immune response, and importantly, BMP signaling (Figures 6F and 6G). This analysis highlights the utility of human lung buds to identify lung-specific gene expression responses to infection by SARS-CoV-2.

### BMP signaling tunes SARS-CoV-2 infection

BMP signaling plays a salient role in tissue remodeling upon lung injury (Cassandras et al., 2020), yet whether it regulates tissue responses to viral infection is unknown. Interestingly, multiple factors involved in the BMP pathway were identified as essential pan-coronavirus host factors in a CRISPR-Cas9 screen that included SARS-CoV-2 (Schneider et al., 2021). As this pathway is also a lung-specific SARS-CoV-2-associated pathway (Figure 6G), we hypothesized it would play an important role in dictating susceptibility to SARS-CoV-2 infection in lung tissue. To test this, we stained SARS-CoV-2-infected lung buds for pSMAD1/5, a marker of active BMP signaling. Interestingly, infected lung buds display an increase in the proportion of cells expressing pSMAD1/5 (Figures 6H and 6I). Furthermore, in these SARS-CoV-2-infected lung buds, pSMAD1/5-positive cells were 2.2-fold more likely to be infected by SARS-CoV-2 (Figure 6J), suggesting that active BMP renders lung cells more susceptible to infection by SARS-CoV-2. Next, to functionally test whether BMP signaling regulates infection, we performed infection experiments with pseudoviruses or native SARS-CoV-2 in the absence of BMP4 and/or with the BMP inhibitor, LDN193189. To do that, we removed BMP or added LDN at day 5 of lung bud formation concomitantly with virus infection and collected infected tissues 48 h post-infection. Intriguingly, we found that pharmacological inhibition of BMP or absence of pathway stimulation by BMP4 leads to a reduction in SARS-CoV-2 infection (Figures 6K and 6L). We did not observe differences in the proportion of alveolar and airway cells nor in the epithelial area of lung buds upon inhibition of BMP by LDN and/or removal of BMP during the treatment window (Figures S5A–S5D), which suggests that the effect of BMP on SARS-CoV-2 infection is independent from its effects on differentiation. As the antiviral effect of BMP signaling inhibition was observed in a pseudovirus assay that measures viral entry, our experiments suggest that BMP signaling plays a role in SARS-CoV-2 entry in lung cells. In agreement with this hypothesis, we observed a decreased expression of ACE2 in lung buds upon inhibition or removal of BMP (Figures S5E and S5F). Thus, BMP activity in lung cells may influence susceptibility to SARS-CoV-2 infection by regulating ACE2 expression.

## Discussion

This work highlights the self-organizing capabilities of lung progenitors when cultured on confined geometries. The human lung bud model recapitulates the earliest events in fetal lung development, which involves the proximodistal coordination of alveolar and airway cellular differentiation and region-specific tissue morphogenetic events (Morrisey and Hogan, 2010; Nikolić et al., 2017, 2018; Rawlins et al., 2009; Zepp and Morrisey, 2019). It also offers key advantages to current *in vitro* models, which includes the standardization of *in vivo*-like tissue organization and complexity, and inexhaustible access to disease-relevant lung tissue. This platform will allow for the interrogation of the molecular and genetic mechanisms orchestrating early human lung differentiation and morphogenesis, in both normal and disease states in which these processes may go awry, such as lung cancer and pulmonary diseases. This platform also provides fast and scalable access to lung tissue for regenerative medicine and modeling lung diseases.

Compared with other lung organoid protocols that display high inter-organoid and batch-to-batch variability (Nikolić et al., 2018), this platform allows for the individual tracking of many lung buds at a time in a highly standardized manner. These lung buds reproducibly self-organize into epithelial structures that display morphogenetic similarities to developing lungs in human embryos (Morrisey and Hogan, 2010; Nikolić et al., 2017, 2018; Rawlins et al., 2009; Zepp and Morrisey, 2019). In particular, lung buds display proximodistal patterning of alveolar and airway tissue reminiscent of fetal counterparts *in vivo* at around week 5 of gestation (Morrisey and Hogan, 2010; Nikolić et al., 2017, 2018; Rawlins et al., 2009; Zepp and Morrisey, 2019). We have demonstrated the utility of lung buds to define a molecular logic during early lung development, including the synergy of multiple signaling pathways for the differentiation and self-organization of multipotent lung progenitors. In particular, we define a salient role of KGF for the self-organization of developing epithelial structures in fetal-like human lung buds, as observed in *in vivo* models (Yano et al., 2000). Although other organoid protocols allow for the generation of tissues that resemble lungs *in vivo*, they lack a controlled self-organization of lung progenitors (Jacob et al., 2017; McCauley et al., 2017; Dye et al., 2015; Miller et al., 2019; Green et al., 2011; Huang et al., 2014, 2015; Chen et al., 2017). Moreover, inter-organoid phenotypic variability and batch-to-batch differences limit their compatibility for the study of genetic determinants of cell fates and how they choreograph tissue morphogenetic events. Thus, human lung buds offer a new paradigm to study early human lung development and human-specific lung biology that would otherwise be impossible to scrutinize.

We highlight the potential of using lung buds to identify novel therapeutics that block infection by SARS-CoV-2, endemic coronaviruses, as well as other respiratory viruses. Compared with 3D organoid models in which tracking virus-induced cellular responses is challenging (Han et al., 2020), this platform allows for the individual tracking of many genetically matched reproducible organoids at a time to gain a quantitative understanding of SARS-CoV-2-induced lung pathology. We have established a highly quantitative platform to assess differential cell type-dependent susceptibilities to infection and cytopathology, as well as to identify key target cell types that are not accessible for experimentation *in vivo*. Notably, we have also identified cycling alveolar cells as targets of SARS-CoV-2, which display tropism for this virus at levels that are similar to non-dividing alveolar cell types. These findings have important clinical implications for COVID-19 as stem cell-like AT2 cells, which display shared key markers with AT1/2s in lung buds, are proposed to be signaling hubs for tissue homeostasis and regeneration in alveoli (Travaglini et al., 2020), and tissue damage in COVID-19 patients may result from the loss of these cells. As intrinsic immunity dictates viral tropism and susceptibility to infection of stem cells (Wu et al., 2018), we highlight potentially novel mechanisms of viral susceptibility in lung tissue. Whether this lung model recapitulates infection in fetal and adult tissue *in vivo* warrants further investigation.

Finally, this platform will help gain an understanding of the cellular mechanisms that direct SARS-CoV-2-induced pathogenesis. Importantly, as this platform is amenable for high-throughput genetic analysis, it will also allow for the discovery of cellular factors that control lung infection, which will identify novel therapeutic targets for COVID-19. Of note, we have demonstrated the utility of lung buds to identify lung-specific gene expression hallmarks of SARS-CoV-2 infection, which led to the identification of BMP signaling as a critical regulator of infection in the lung. Further evidence for the role of BMP signaling in entry is due to the identification of BMP as a host factor in pooled screens that naturally bias toward factors related to viral entry (Schneider et al., 2021). Future studies may use this platform for rapid and scalable genome-wide screening for essential host factors (Hoffmann et al., 2021; Schneider et al., 2021) and small molecule screens. Finally, this model will also help delineate how patient mutations may modify disease progression in respiratory lung diseases such as COVID-19 (Zhang et al., 2020), and provides the hope to have a transformative influence in the elucidation of the cellular and molecular basis of respiratory infections and lung diseases for which there are currently no therapies.

## Experimental procedures

### Resource availability

#### Corresponding authors

Ali H. Brivanlou (brvnlou@rockefeller.edu) and Charles M. Rice (ricec@rockefeller.edu).

#### Materials availability

The materials included in the current study are available from the corresponding authors on reasonable request.

### Maintenance of hESCs

RUES2 (NIHhESC-09-0013), RUES2-GLR (Figure S1) (Martyn et al., 2018), and HUES8 iCas9 (NIHhESC-09-0021; Figure S3) hESC lines were used in this study and maintained in HUESM (DMEM supplemented with 20% knockout serum replacement, 1×B27 supplement without vitamin A, 0.1 mM non-essential amino acids, 2 mM GlutaMax, and 0.1 mM 2-mercaptoethanol) conditioned by mouse embryonic fibroblasts (MEF-CM) and supplemented with 20 ng^∗^mL^−1^ basic fibroblast growth factor. The cells were grown at 37°C and 5% CO2 on tissue culture dishes that were coated with Geltrex (Life Technologies) solution.

### Generation of lung buds in micropatterns

hESCs were first differentiated into definitive endoderm using the STEMDiff Definitive Endoderm Kit (Stem Cell Technologies), with 1 day addition of supplement CJ and MR and 2 days addition of supplement CJ only. Upon endoderm induction, cells were washed once with PBS^−/−^ (Gibco) and dissociated with Accutase (Stem Cell Technologies) for 7 min. Cells were then dissociated with a pipette to ensure a single-cell suspension and diluted four times in complete serum-free differentiation medium (cSFDM) containing DMEM/F12 (Gibco) with B27 Supplement with RA (Invitrogen, Waltham, MA), N2 Supplement (Invitrogen), 0.1% bovine serum albumin Fraction V (Invitrogen), β-mercaptoethanol (Sigma), Glutamax (ThermoFisher), 50 μg^∗^mL^−1^ ascorbic acid (Sigma), and normocin with supplements of 10 μm SB431542 (“SB”; Tocris), 2 μm Dorsomorphin (“DS”; Stemgent) and 10 μm ROCK inhibitor (Y-27632; Abcam). Cells were further diluted with the same medium and 5 × 10^5^ cells in 3.0 mL of medium were placed over a laminin-coated micropattern glass coverslips (CYTOOCHIPS Arena A, Arena 500A, Arena EMB A, Arena 225A) in a 35-mm tissue culture dish, left untouched for 10 min and then incubated at 37° C. After 3 h, the micropattern was washed once with PBS^+/+^, which was then replaced with cSFDM with 10 μm SB and 2 μm DM. Two days later, the media were replaced with cSFDM with 10 μm SB and 2 μm DM. The next day, micropattern cultures were fed in lung induction medium (LIM), which contains cSFDM supplemented with 50 ng^∗^mL^−1^ KGF (or indicated in each experiment; R&D systems), 10 ng^∗^mL^−1^ BMP4 (R&D systems), 100 nM retinoic acid (“RA”; Sigma-Aldrich), and 3 μM CHIR9902 (EMD Millipore). Micropattern cultures were fed every other day in LIM for 7 days or until tissues were collected for analysis.

To generate lung buds on 96-well plates, 4 × 10^4^ definitive endoderm cells were seeded on laminin-coated 96-well plates (CYTOOPlates, 200A), left untouched for 1 h, and then incubated at 37° C for 3 h in cSFDM with 10 μm SB, 2 μm DM, and 10 μm ROCK inhibitor. After this point, the protocol described above to generate lung buds on coverslips was followed.

### Imaging

All confocal images were acquired on a Zeiss Inverted LSM 780 laser scanning confocal microscope with a ×10, ×20, or ×25 oil-immersion objective; 96-well plates were imaged on an ImageXpress Micro with a ×10 objective. Three-dimensional visualization and image processing was performed in ImageJ.

### Imaging analysis

Analysis of confocal Z-stacks was performed using CellProfiler v4.0.4 (https://cellprofiler.org/). DAPI staining was used to identify nuclei and cytoplasm. Cells were classified as positive for a given marker based on the identification of fluorescence signals in either nuclear (for nuclear markers such as SOX9 and SOX2) or cytoplasmic area (SARS-CoV-2 and aCASP3) to estimate the percentage of marker-positive across Z-stacks.

Ninety-six-well plates image analysis was performed using ImageJ. Briefly, nuclei were identified and set to a mask. The regions of interest were then expanded, creating a new mask to interrogate cytoplasmic signals of virus infection in the SARS-CoV-2 images.

### SARS-CoV-2 infection and transmission analysis

SARS-CoV-2 (strain: USA-WA1/2020) and HCoV-NL63 were obtained from BEI Resources (NR-52281 and NR-470). HCoV-OC43 was obtained from ZeptoMetrix (cat. #0810024CF) and HCoV-229E was generously provided by Volker Thiel (University of Bern). All viruses were amplified at 33°C in Huh-7.5 cells to generate a P1 stock. To generate working stocks, Huh-7.5 cells were infected at a multiplicity of infection (MOI) of 0.01 plaque-forming units (PFU)/cell (SARS-CoV-2, HCoV-NL63, HCoV-OC43) and 0.1 PFU/cell (HCoV-229E) and incubated at 33°C until virus-induced CPE was observed. Supernatants were subsequently harvested, clarified by centrifugation (3,000 × *g* × 10 min) at 4 dpi (HCoV-229E), 6 dpi (SARS-CoV-2, HCoV-OC43), and 10 dpi (HCoV-NL63), and aliquots stored at −80°C.

Viral titers were measured on Huh-7.5 cells by standard plaque assay. Briefly, 500 μL of serial 10-fold virus dilutions in Opti-MEM were used to infect 4 × 10^5^ cells seeded the day prior into wells of a six-well plate. After 90-min adsorption, the virus inoculum was removed, and cells were overlaid with DMEM containing 10% FBS with 1.2% microcrystalline cellulose (Avicel). Cells were incubated for 4 days (HCoV-229E), 5 days (SARS-CoV-2, HCoV-OC43), and 6 days (HCoV-NL63) at 33°C, followed by fixation with 7% formaldehyde and crystal violet staining for plaque enumeration. All SARS-CoV-2 experiments were performed in a biosafety level 3 laboratory.

SARS-CoV-2 pseudotyped viruses were generated as described before using a C-terminally truncated SARS-CoV-2 S protein co-transfected with pNL4-3ΔEnv-nanoluc reporter (Robbiani et al., 2020). Infection by SARS-CoV pseudotyped viruses was quantified 48 h post-infection by measuring Nanoluc Luciferase activity in lysates using the Nano-Glo Luciferase Assay System (Promega) with Synergy *Neo*2 Multi-mode Microplate Reader (BioTek).

### Antibody treatments

Neutralization assays were performed as previously described (Robbiani et al., 2020). Briefly, antibodies were serially diluted in LIM, mixed with a constant amount of SARS-CoV-2 (2 × 10^5^ PFU for the larger chips, and 5 × 10^4^ PFU in the 96-well plate assays) and incubated for 60 min at 37°C. The antibody-virus mix was then added to the 96-well plates or microchip containing human lung buds.

### Statistical analysis

Statistical analysis was performed using unpaired two-sided t test and one-way ANOVA multiple comparison tests, unless stated otherwise. For all the experiments included in this study, three or more independent experiments were included using stage-matched controls as a reference. No statistical analysis was used to predetermine sample size and no data were excluded.

Detailed experimental procedures for immunostaining, scRNA-seq, and bulk RNA-seq analysis are included in the supplemental experimental procedures.

## Author contributions

E.A.R. and A.H.B. conceived and designed the lung bud platform. E.A.R., B.R., H.H., C.M.R., and A.H.B. designed experiments with SARS-CoV-2. E.A.R. and B.R. executed and analyzed the experiments. E.A.R. and R.D.S. performed the single-cell gene expression analysis. Z.S. contributed to imaging and data analysis. J.L.P., D.B., T.S.C., and J.P. performed bioinformatic analysis of bulk RNA-seq data. E.A.R., B.R., and J.T.P. contributed to the identification and genetic analysis of BMP signaling in infected lung tissue. E.A.R., B.R., C.M.R., and A.H.B. wrote the manuscript with input from all authors.

## Data Availability

The datasets generated during and/or analyzed during the current study are available from the corresponding authors on reasonable request. RNA-seq data are available through the NCBI GEO accession numbers GSE163698 and GSE225564.

## Supplemental information

Document S1. Figures S1–S5 and supplemental experimental procedures

Document S2. Article plus supplemental information
