# Supplementary material for: Organotypic human lung bud microarrays identify BMP-dependent SARS-CoV-2 infection in lung cells
Source: Stem Cell Reports. 2023 Apr 20;18(5):1107–22. doi: 10.1016/j.stemcr.2023.03.015 (PMC10116630; doi:10.1016/j.stemcr.2023.03.015)
Supplement: Document S1. Figures S1–S5 and supplemental experimental procedures [file mmc1.pdf]

**Stem Cell Reports, Volume *18***

# Supplemental Information

# Organotypic human lung bud microarrays identify BMP-dependent SARS-CoV-2 infection in lung cells

**E.A. Rosado-Olivieri, B. Razooky, J. Le Pen, R. De Santis, D. Barrows, Z. Sabry, H.-H. Hoffmann, J. Park, T.S. Carroll, J.T. Poirier, C.M. Rice, and A.H. Brivanlou**

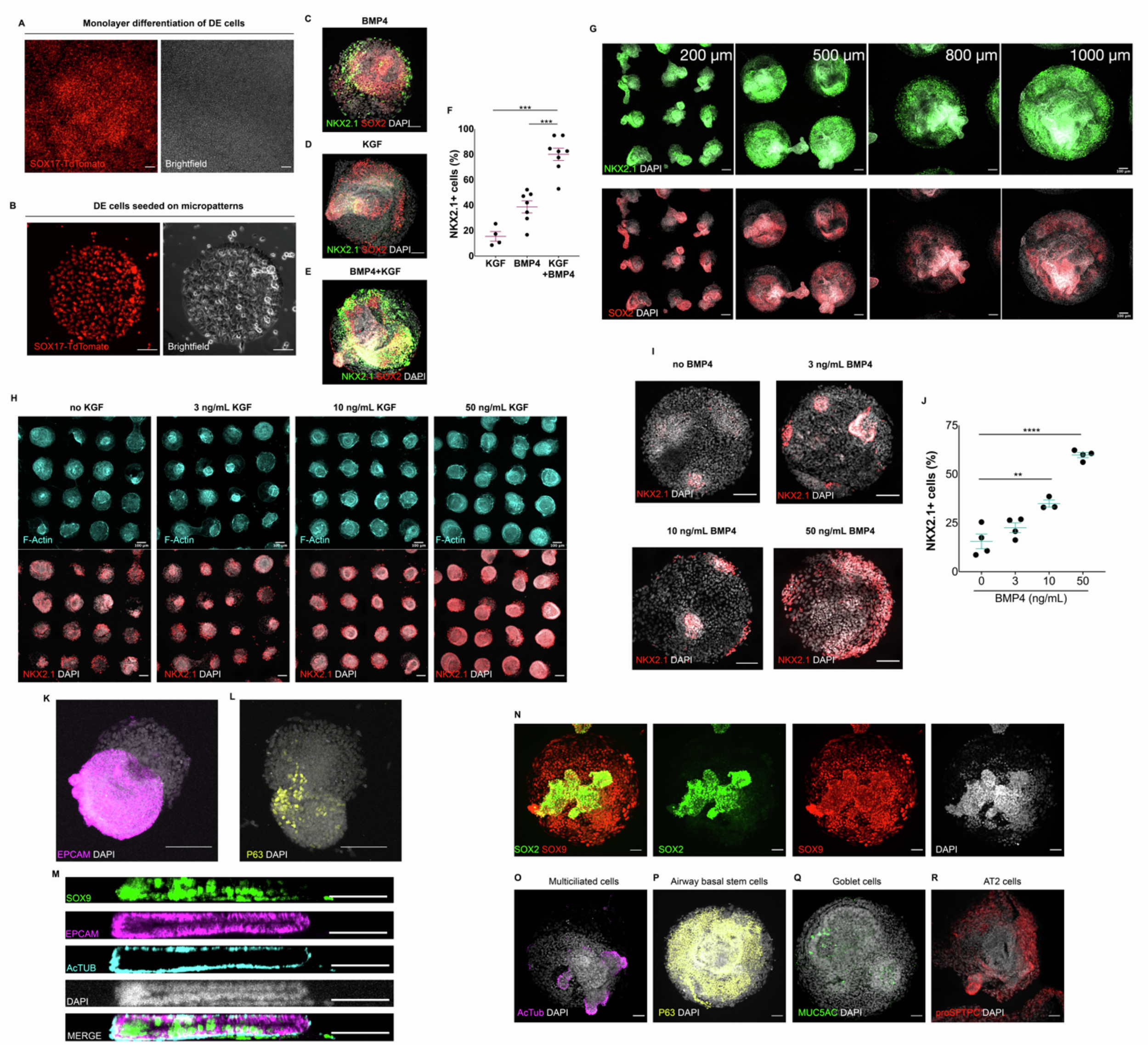


**Supplementary Figure 1: Induction of NKX2.1+ lung progenitors on confined geometries, related to Figure 1.** A) Monolayer differentiation of SOX17+ endoderm progenitors pre-seeding. B) SOX17+ endoderm progenitors on confined geometry 3 hours post-seeding. C-F) Induction of NKX2.1+ multipotent lung and SOX2+ airway progenitors upon modulation of BMP4, KGF or KGF+BMP4. (scale bar: 50 µm). Experiments in A-E were performed using the RUES2-GLR cell lines (N=4 independent experiments). G) Efficient induction of NKX2.1+ multipotent lung and SOX2+ airway progenitors in colonies of varying sizes. (scale bar: 100 µm). H) Low magnification images of epithelial buds containing NKX2.1+ lung progenitors grown on confined geometries of 225 µm diameter at varying doses of KGF. Epithelial structures can be identified with F-actin staining. I-J) Induction of NKX2.1+ lung progenitors on confined geometries of 500 µm diameter at varying doses of BMP4. K-M) Top and side-view of lung buds expressing EPCAM (K), P63 (L), SOX9 (M) and AcTub (M) along proximo-distal axis. N-R) Identification of SOX2+ and SOX9+ progenitors as well as AcTub+ multiciliated cells (O), P63+ airway basal stem cells (P), MUC5AC+ goblet cells (Q) and proSFTPC+ type 2 pneumocytes (R) in 500 µm colonies. (scale bar: 50µm)

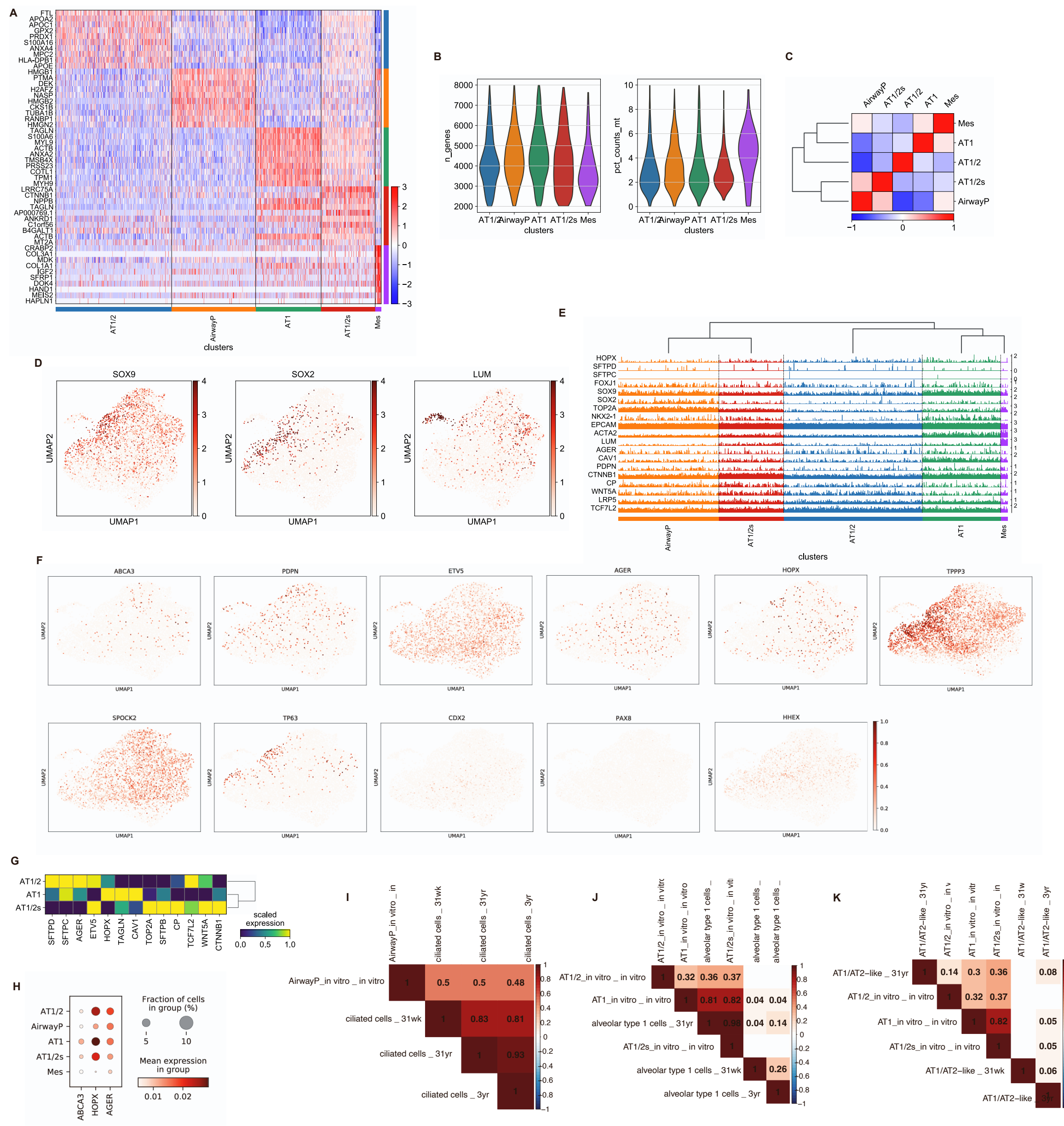


**Supplementary Figure 2: Single-cell gene expression analysis of lung buds, related to Figure 2.** A) Heatmap of top 10 differentially expressed genes for each cluster identified in synthetic lung buds. z-score normalized expression values are shown. B) Violin plots of the number of genes (n_genes) and percentage of mitochondrial genes (pct_counts_mt) for each cluster identified in synthetic lung buds. C) Cluster-level gene expression Pearson correlation analysis of clusters identified in synthetic lung buds. z-score normalized correlation values are shown. D) UMAP expression plots of SOX9, SOX2 and lumican (LUM). E) Gene expression trackplots of cell type-specific markers. Each peak represents a single cell and its height denotes the expression level of each gene. F) UMAP expression plots of alveolar, airway, hepatic, thymic and intestinal markers. G-H) Heatmap and dotplot of scaled gene expression levels of alveolar markers. I-K) Cluster-level stage-dependent gene expression Pearson's correlation analysis of airway (I) and alveolar cell types (J-K) identified in *in vitro*-derived lung buds and in adult lung tissue at different developmental stages.

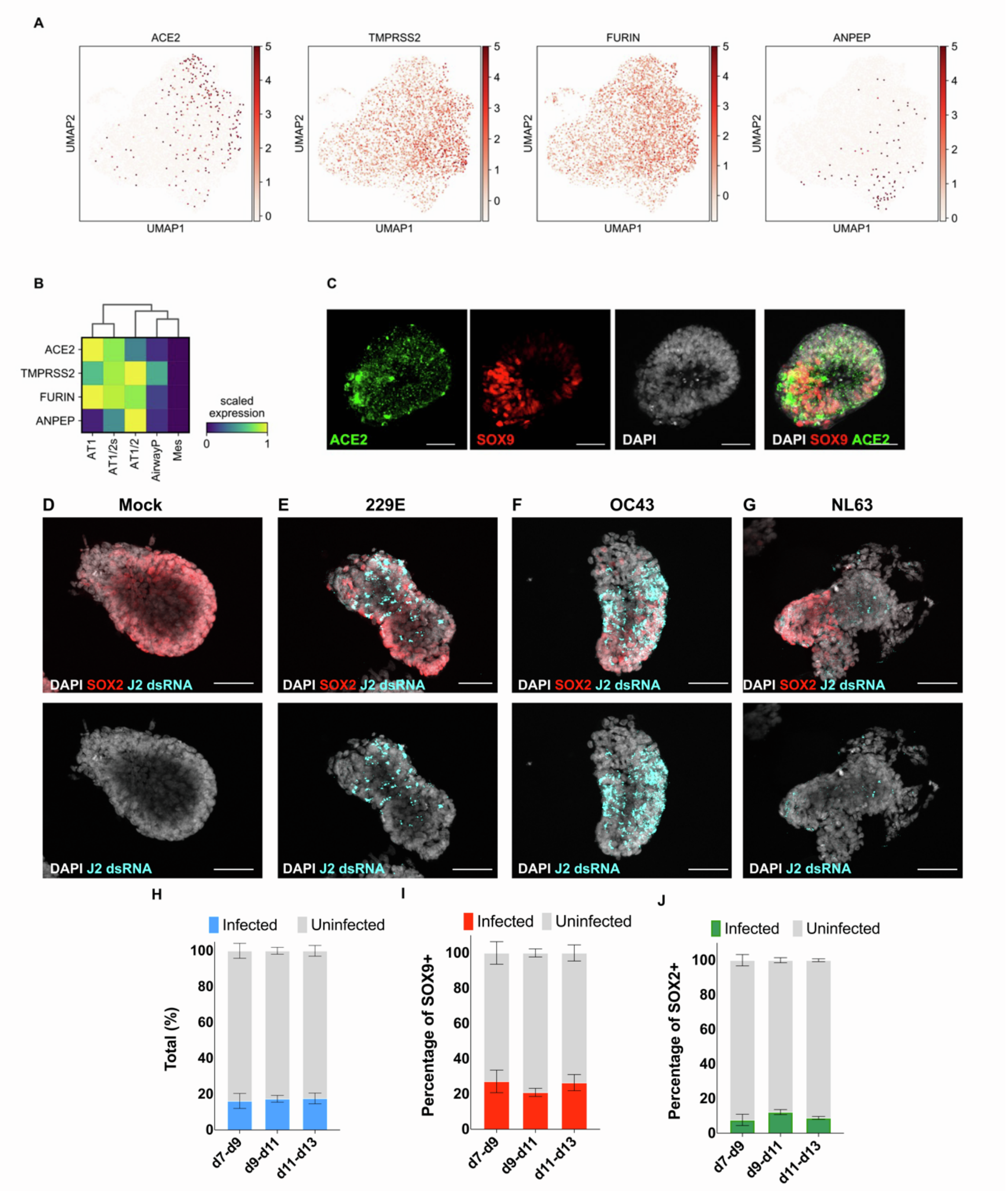


**Supplementary Figure 3: Expression of coronaviruses-associated genes and infection by endemic coronaviruses, related to Figure 3.** A) UMAP expression plots of ACE2, TMPRSS2, FURIN and ANPEP. B) Scaled expression of SARS-CoV-2 entry factors for each of the identified clusters in synthetic human lung buds. C) Expression of the ACE2 receptor in *in vitro*-derived lung buds. D-G) Synthetic lung buds infected with endemic coronaviruses HCoV-229E (E), HCoV-OC43 (F) and HCoV-NL63 (G). Infected cells were identified by staining with J2 antibody detecting dsRNA. H-J) Percentage of total (H), SOX9+ (I) and SOX2+ (J) cells infected by SARS-CoV-2 at multiple stages of lung bud formation (N=9 independent experiments). (scale bar: 50μm)

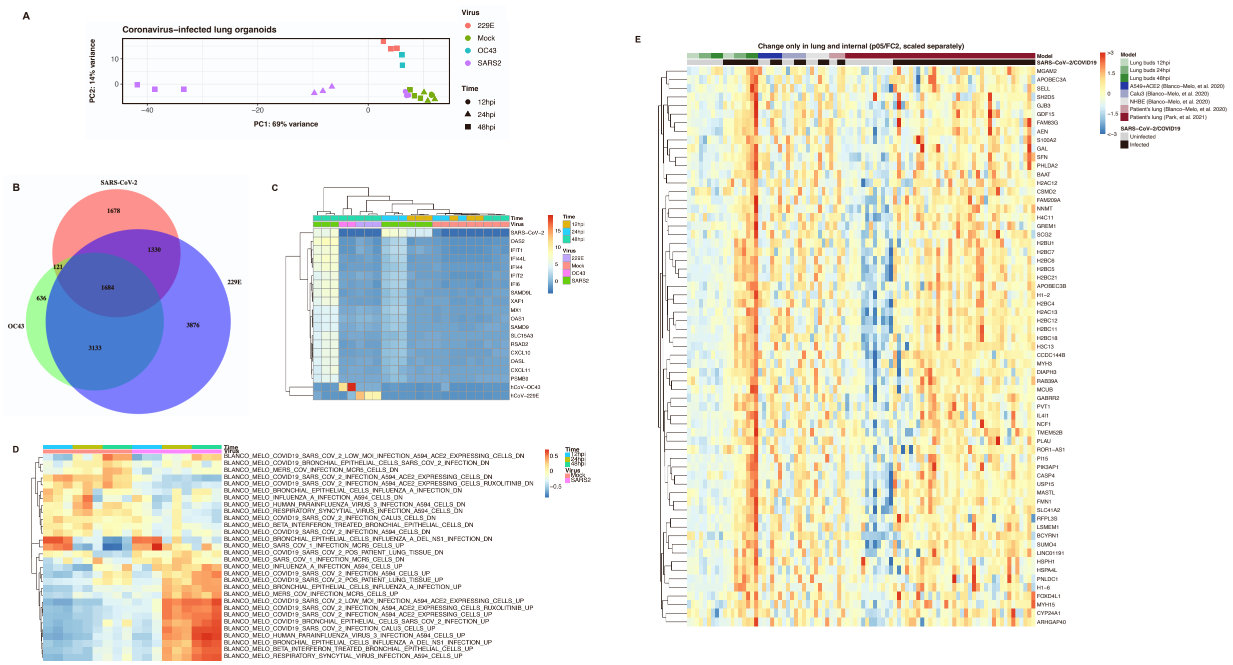


**Supplementary Figure 4: Gene expression analysis of infected synthetic lung buds, related to Figure 6.** A) PCA analysis of gene expression of infected and mock synthetic lung buds 12, 24, and 48 hpi. B) Venn diagram of DEG in synthetic lung buds collected 48 hpi after infection by SARS-CoV-2, OC43 or 229E. C) Heatmap of the expression of top 20 SARS-CoV-2-associated DEG. D) Heatmap of enrichment scores of COVID-19-associated gene sets in each sample from Blanco-Melo et al., 2020[38]. E) Heatmap of genes identified to be up-regulated in SARS-CoV-2-infected synthetic lung buds and postmortem lung tissue but not cancer cell lines.

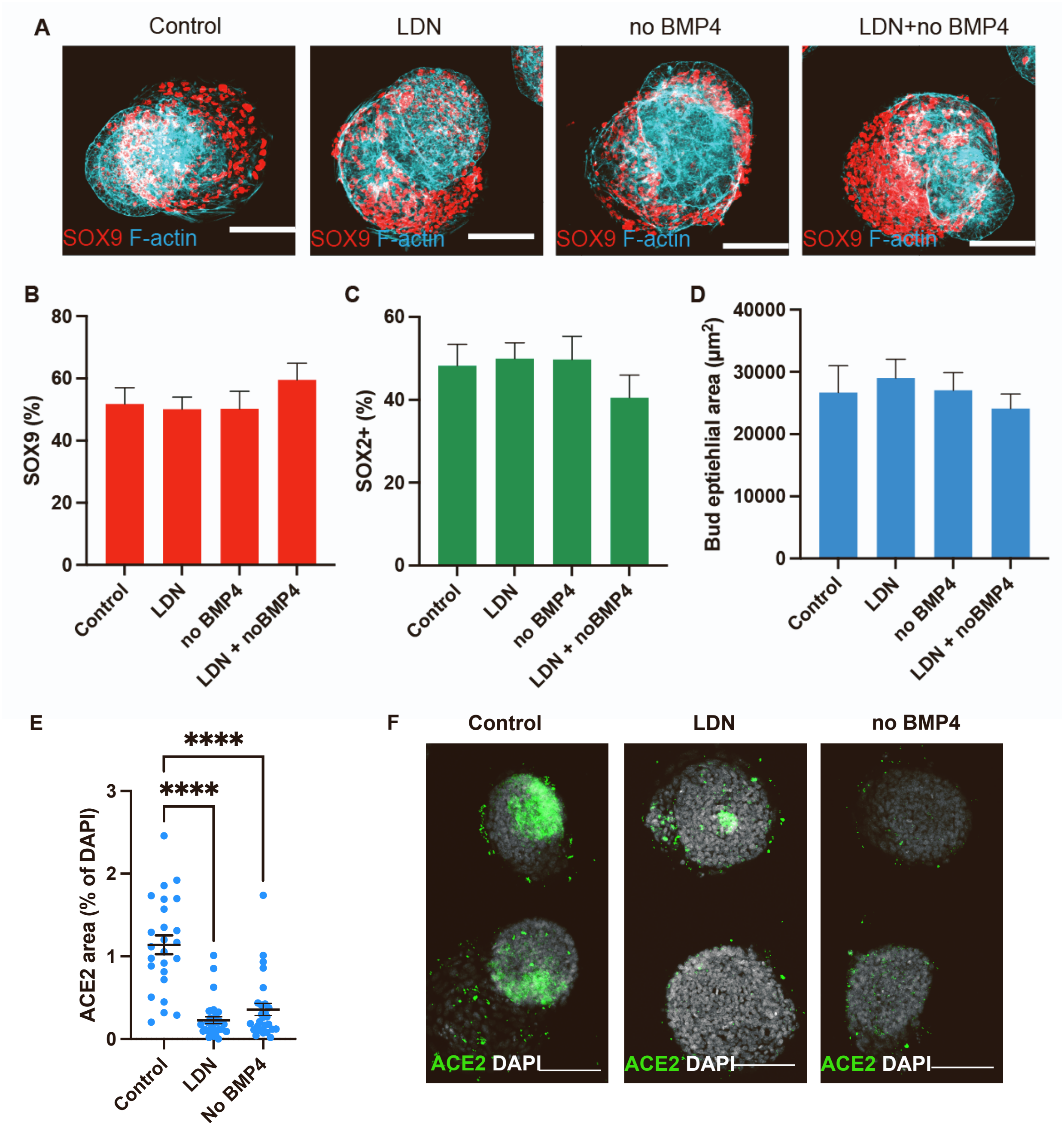


**Supplementary Figure 5: Effect of BMP inhibition on lung buds and ACE2 expression.** A) Expression of SOX9 and F-actin in lung buds treated with LDN and/or no BMP4 (scale bar: 50µm). B-C) Quantification of SOX9+ alveolar and SOX2+ airway cells. D) Quantification of epithelial area. E-F) Expression of ACE2 in lung buds upon BMP inhibition or removal. (N=3 independent experiments) (scale bar: 100µm)

## Supplementary Experimental Procedures

### Immunostaining

Micropattern coverslips were fixed with 4% paraformaldehyde (Electron Microscopy Sciences 15713) in warm medium for 30 min, rinsed three times with $PBS^{-/-}$, and then blocked and permeabilized with 3% normal donkey serum (Jackson Immunoresearch 017-000-121) with 0.5% Triton X-100 (Sigma 93443) in $PBS^{-/-}$ for 30 min. Micropatterns were incubated with primary antibodies for 1.5 h, washed three times in $PBS^{-/-}$ for 5 min each, incubated with secondary antibodies conjugated with Alexa 488, Alexa 555, Alexa 594 or Alexa 647 (1:1,000 dilution, Molecular Probes), fluorescently-conjugated phalloidin (1:400; Life Technologies) and 10 $ng*ml^{-1}$ of DAPI (Thermo Fisher Scientific D1306) for 30 min and then washed three times with $PBS^{-/-}$. Coverslips were mounted on slides using ProLong Gold antifade mounting medium (Molecular Probes P36934).

The primary antibodies used were as follows: rabbit anti-SOX9 (Millipore; AB5535; 1:250), goat anti-SOX2 (R&D Systems; AF2018; 1:250), rabbit anti-NKX2.1 (Abcam; ab76013; 1:200), mouse anti-Acetylated Tubulin (Sigma; T7451; 1:1000), goat anti-TP63 (R&D Systems; BAF1916; 1:250), rabbit anti-proSPC (Seven Hills; WRAB-9337; 1:500), mouse anti-HOPX (Santa Cruz; sc-398703; 1:250), rabbit anti-nucleocapsid SARS-CoV-2 (GeneTex; GTX135357; 1;1000); rabbit anti-Active Caspase-3 (R&D Systems; AF835; 1:250); mouse anti-Mucin 5AC (Abcam; ab3649; 1:250); human anti-Spike SARS-CoV-2 (1:1,000) (Robbianit et al., 2020); anti-HNF-3BETA/FOXA2 (Neuromics; GT15186; 1:200); mouse J2 dsRNA (SCICONS; 1;1,000), phospho Histone H3 (Cell Signaling; 9706S; 1:200), goat anti-SOX17 (R&D Systems; AF1924; 1:200), rabbit anti-pSMAD1/5 (Cell Signaling; 9516; 1:200) and goat anti-AGER (R&D Systems; AF1145; 1:200). To detect infected cells for HCoV-229E, HCoV-OC43 and HCoV-NL63, a mouse monoclonal anti-dsRNA antibody (Scicons: catalog no. 10010500) was used under similar conditions.

### Single cell RNA-sequencing analysis

Micropatterned coverslips with 225-µm diameter synthetic lung buds at day 7 of lung induction were dissociated with TrypLE Express (Gibco) for 10 min at 37 °C. After dissociation, the cells

were washed three times in PBS$^{-/-}$ (Gibco) with 0.04% BSA and strained through a Flowmi tip 40 µm strainer. Cell count and viability were determined on a Countess II Automated Cell Counter. Samples were loaded for capture with the Chromium System using the Single Cell 3′ v3 reagents (10X Genomics). Following cell capture and lysis, cDNA was synthesized and amplified according to the manufacturer's instructions (10X Genomics). The resulting libraries were sequenced on the NovaSeq platform. The Cell Ranger (v.2.0.2) software pipeline was used to create FASTQ files which were aligned to the hg19 genome using default parameters. These data are available through the NCBI GEO accession number GSE163698.

Filtered gene expression matrices were generated using Cell Ranger and subsequently used for downstream analyses using Scanpy (v.1.6.0) (https://pypi.org/project/scanpy/). Data was filtered to have a minimum of 2000 and a maximum of 8000 detected genes per cell and genes were filtered to be expressed in at least 3 cells. Cells with over 10% mitochondrial genes were discarded. Clustering of cells was performed using the Leiden algorithm (resolution=0.4) and visualized using UMAP plots (n.neighbors = 50, PCA components= 50). Differential gene expression analysis was performed using the Wilcoxon rank-sum (Mann-Whitney-U) test to identify cluster markers.

To compute correlations with human lung tissue, we performed data integration of our dataset with a published single cell dataset of lung tissue from a 30wk, 3 yr and 30 yr old donors (Wang et al. 2020). We integrated these datasets using canonical correlation analysis (Seurat v4.0) (Stuart et al. 2019) with FindIntegrationAnchors() and IntegrateData() using the first 30 principal components. After integration, we estimated the average expression value for each gene in each cell type and computed Pearson's correlations for all possible pairs of cell types with rcorr(). Significant correlation values (adjusted p-value < 0.05) were plotted with corrplot(). Hierarchical clustering based on correlation coefficients was performed with hclust().

**RNA-sequencing analysis**

Synthetic lung buds were collected 12, 24 and 48 hpi for total RNA extraction. The quality of RNA samples was determined using an Agilent 2100 Bioanalyzer, and all samples for sequencing had

RNA integrity (RIN) numbers of more than 8. Poly(A) selection and library preparation using an Illumina TrueSeq mRNA sample preparation kit, and sequencing on a NovaSeq SP 2x50 platform.

Reads were trimmed and quality checked with Trim Galore! v0.6.6 powered by Cutadapt v1.18 (Kechin et al. 2017); command: trim_galore -q 20 --fastqc -e 0.1 --length 20 --paired --path_to_cutadapt cutadapt <read1> <read2>. Next, reads were mapped on a combined human genome (GRCh38.p13), HCoV-229E genome (GenBank: NC_002645.1) (Thiel et al. 2001), HCoV-OC43 genome (GenBank: NC_006213) (St-Jean et al. 2004), and SARS-CoV-2 genome (GenBank: MN985325.1) (Harcourt et al. 2020) with STAR v2.7.6a (Dobin et al. 2013); command: STAR --readFilesIn <read1> <read2> --outFileNamePrefix <prefix> --genomeDir <combined_genome> --readFilesCommand zcat --runThreadN 12 --chimScoreJunctionNonGTAG -1 --outSAMtype BAM SortedByCoordinate --chimOutType Junctions SeparateSAMold --alignSJDBoverhangMin 5 --outFilterMultimapScoreRange 1 --outFilterMultimapNmax 5 --outMultimapperOrder Random --outSAMattributes NH HI AS nM NM XS ch --chimSegmentMin 10 --chimJunctionOverhangMin 10 --chimScoreMin 1 --chimScoreDropMax 30 --chimScoreSeparation 7 --chimSegmentReadGapMax 3 --chimFilter None --twopassMode None --alignSJstitchMismatchNmax 5 -1 5 5 --chimMainSegmentMultNmax 10. Resulting bam files were tagged with read groups and merged by sample using Picard tools v2.23.6 (http://broadinstitute.github.io/picard/), and indexed with samtools v1.3.1 (Li et al. 2009). Genes were counted using features_count powered by Rsubread v2.2.6 in R v4.0.2 (Liao et al. 2019), using recommended parameters for stranded paired-end RNAseq and a combined human/HCoV-229E/HCoV-OC43/SARS-CoV-2 annotation GTF file. Estimation of variance–mean dependence from the count data and principal component analysis was performed with DESeq2 v1.28.1 (Love et al. 2014) using R v4.0.2, using the constructor function DESeqDataSetFromMatrix(design = ~virus + time). All the result tables were built using the DESeq2 results() function.

Sequence and transcript coordinates for mouse mm10 UCSC genome and gene models were retrieved from the BSgenome.Hsapiens.UCSC.hg38 Bioconductor package (version 1.4.1) and TxDb.Mmusculus.UCSC.hg38.knownGene (version 3.4.0) Bioconductor libraries, respectively. Transcript expressions were calculated using the Salmon quantification software (Patro et al. 2017)

and gene expression levels as TPMs and counts retrieved using Tximport[52] (version 1.8.0) (Love et al. 2015). Normalization and rlog transformation of raw read counts in genes were performed using DESeq2 (version 1.20.0) (Love et al. 2016). Gene set enrichment analysis (GSEA), over representation analysis, and visualization of the enriched gene ontology/pathway terms as network plots were performed with the clusterProfiler R Bioconductor package (version 3.18.1) (Yu et al. 2012, Subramanian et al. 2005). Gene set variation analysis (GSVA) was done on the RNAseq counts matrix (normalized to sequencing depth) using the GSVA R Bioconductor package (version 1.38.2) (Hänzelmann et al. 2013). Published COVID19 gene lists for the GSVA analysis were extracted from the Molecular Signatures Database (MSigDB) using the msigdbr R package (version 7.2.1). Heatmaps (for both GSVA and gene expression) were generated using the pheatmap R package (Kolde et al. 2019). These data are available through the NCBI GEO accession number GSE225564.

## References


Dobin et al. (2013) STAR: ultrafast universal RNA-seq aligner. Bioinformatics 29(1):15-21.

Hänzelmann S, Castelo R, Guinney J (2013). "GSVA: gene set variation analysis for microarray and RNA-Seq data." BMC Bioinformatics, 14, 7. doi: 10.1186/1471-2105-14-7, http://www.biomedcentral.com/1471-2105/14/7.

Harcourt et al. (2020) Severe Acute Respiratory Syndrome Coronavirus 2 from Patient with Coronavirus Disease, United States. Emerg Infect Dis. 26(6):1266-1273.

Kechin et al. (2017) cutPrimers: A New Tool for Accurate Cutting of Primers from Reads of Targeted Next Generation Sequencing. J. Comput. Biol. 24(11):1138-1143.

Kolde, R. pheatmap: Pretty Heatmaps. R package version 1.0.12 (2019).

Li et al. (2009) The Sequence Alignment/Map format and SAMtools. Bioinformatics 25(16):2078-9.

Liao et al. (2019) The R package Rsubread is easier, faster, cheaper and better for alignment and quantification of RNA sequencing reads. Nucleic Acids Res 47(8):e47.

Love MI, Hogenesch J, Irizarry R. (2016) Modeling of RNA-seq fragment sequence bias reduces systematic errors in transcript abundance estimation. Nat Biotechnol. 34(12):1287-1291

Love, MI et al. (2014) Moderated estimation of fold change and dispersion for RNA-seq data with DESeq2. Genome Biol 15(12):550.

Patro R, Duggal G, Love MI, Irizarry RA, Kingsford C. (2017) Salmon provides fast and bias-aware quantification of transcript expression. Nat Methods. 14(4):417-419.

Robbiani et al. (2020) Convergent antibody responses to SARS-CoV-2 in convalescent individuals. Nature 584, 437–442.

St-Jean et al. (2004) Human respiratory coronavirus OC43: genetic stability and neuroinvasion. J Virol. 78(16):8824-34.

Subramanian, Tamayo, et al. Gene set enrichment analysis: A knowledge-based approach for interpreting genome-wide expression profiles 2005, PNAS 102, 15545-15550

Thiel et al. (2001) Infectious RNA transcribed in vitro from a cDNA copy of the human coronavirus genome cloned in vaccinia virus. J Gen Virol. 82, 1273-1281.

Yu G, Wang L, Han Y, He Q (2012). "clusterProfiler: an R package for comparing biological themes among gene clusters." OMICS: A Journal of Integrative Biology, 16(5), 284-287.
