# Supplementary material for: Organotypic human lung bud microarrays identify BMP-dependent SARS-CoV-2 infection in lung cells
Source: Stem Cell Reports. 2023 Apr 20;18(5):1107–22. doi: 10.1016/j.stemcr.2023.03.015 (PMC10116630; doi:10.1016/j.stemcr.2023.03.015)
Supplement: Document S2. Article plus supplemental information [file mmc2.pdf]

Stem Cell Reports
Article
OPEN ACCESS

Organotypic human lung bud microarrays identify BMP-dependent SARS-CoV-2 infection in lung cells

E.A. Rosado-Olivieri,[1,5] B. Razooky,[2,5] J. Le Pen,[2] R. De Santis,[1] D. Barrows,[3] Z. Sabry,[1] H.-H. Hoffmann,[2] J. Park,[2] T.S. Carroll,[3] J.T. Poirier,[4] C.M. Rice,[2,*] and A.H. Brivanlou[1,*]
[1]Laboratory of Synthetic Embryology, the Rockefeller University, New York, NY, USA
[2]Laboratory of Virology and Infectious Diseases, the Rockefeller University, New York, NY, USA
[3]Bioinformatics Resource Center, the Rockefeller University, New York, NY, USA
[4]Laura and Isaac Perlmutter Cancer Center, New York University Grossman School of Medicine, NYU Langone Health, New York, NY, USA
[5]These authors contributed equally
*Correspondence: ricec@rockefeller.edu (C.M.R.), brvnlou@rockefeller.edu (A.H.B.)
https://doi.org/10.1016/j.stemcr.2023.03.015

SUMMARY

Although lung disease is the primary clinical outcome in COVID-19 patients, how SARS-CoV-2 induces lung pathology remains elusive. Here we describe a high-throughput platform to generate self-organizing and commensurate human lung buds derived from hESCs cultured on micropatterned substrates. Lung buds resemble human fetal lungs and display proximodistal patterning of alveolar and airway tissue directed by KGF. These lung buds are susceptible to infection by SARS-CoV-2 and endemic coronaviruses and can be used to track cell type-specific cytopathic effects in hundreds of lung buds in parallel. Transcriptomic comparisons of infected lung buds and postmortem tissue of COVID-19 patients identified an induction of BMP signaling pathway. BMP activity renders lung cells more susceptible to SARS-CoV-2 infection and its pharmacological inhibition impairs infection by this virus. These data highlight the rapid and scalable access to disease-relevant tissue using lung buds that recapitulate key features of human lung morphogenesis and viral infection biology.

INTRODUCTION

The emergence of SARS-CoV-2 in late 2019 sparked an explosive global pandemic of COVID-19 disease, with >665 million confirmed cases and >6.7 million deaths to date (Zhou et al., 2020; Zhu et al., 2020; World Health Organization, 2023). The rational design of COVID-19 therapies will require an understanding of the life cycle of the virus during infection in human cells. Key infection routes of SARS-CoV-2 involve the nasal passages, lung airways, and alveoli (Zhou et al., 2020; Zhu et al., 2020; Hou et al., 2020). In particular, the lung is the one of the most vulnerable target organs for SARS-CoV-2, as acute lung injury and pneumonia-associated complications are primary clinical outcomes in severe cases of COVID-19 (Zhou et al., 2020; Zhu et al., 2020; Hou et al., 2020). In this organ, airway multi-ciliated cells, alveolar type 2 (AT2) pneumocytes, and club cells are the primary targets of the virus (Hou et al., 2020; Muus et al., 2020; Sungnak et al., 2020; Ziegler et al., 2020). However, how SARS-CoV-2 induces local tissue damage and pathology in the lungs is not completely understood (Zhou et al., 2020; Zhu et al., 2020; Hou et al., 2020).

Current models of SARS-CoV-2 lung infection rely on *in vitro* cultures of human primary lung tissue (Hou et al., 2020; Katsura et al., 2020; Youk et al., 2020; Salahudeen et al., 2020; Tindle et al., 2021; Lamers et al., 2021), which remains challenging due to its irregular nature as well as its high inter-donor phenotypic and genetic variability. These features are of particular importance as genetic heterogeneity plays a large role in SARS-CoV-2 replication and outcome (Williamson et al., 2020; Zhang et al., 2020). To circumvent these limitations, protocols have been developed to differentiate human embryonic stem cells (hESCs) into lung airway and alveolar cells as an alternate source of tissue to study lung biology and disease (Jacob et al., 2017; McCauley et al., 2017; Dye et al., 2015; Miller et al., 2019; Green et al., 2011; Huang et al. 2014, 2015; Chen et al., 2017). hESCs-derived lung tissues have been used to study cellular responses upon SARS-CoV-2 infection and to identify small molecules that halt SARS-CoV-2 infection (Han et al., 2020; Huang et al., 2020). This highlights the potential of using hESC-based platforms for the study of SARS-CoV-2-associated lung pathology and for high-throughput identification of therapeutics for COVID-19. A caveat of stem cell-based models of lung tissue is that, despite providing an inexhaustible supply of human lung cells, they lack the controlled tissue organization observed in developing and adult lung tissue such as the coordinated segregation of alveolar and airway tissues (Morrisey and Hogan, 2010; Nikolić et al., 2018). Moreover, current protocols take 1 to 3 months to differentiate lung cells from hESCs, underscoring a need to develop fast and scalable platforms to generate these cells *in vitro*. Finally, many of these organoid systems suffer from inter-organoid variability and reproducibility (Nikolić et al., 2018), which limits their compatibility with chemical and genetic screening at a high-throughput scale. In this article, we

This is an open access article under the CC BY license (http://creativecommons.org/licenses/by/4.0/).

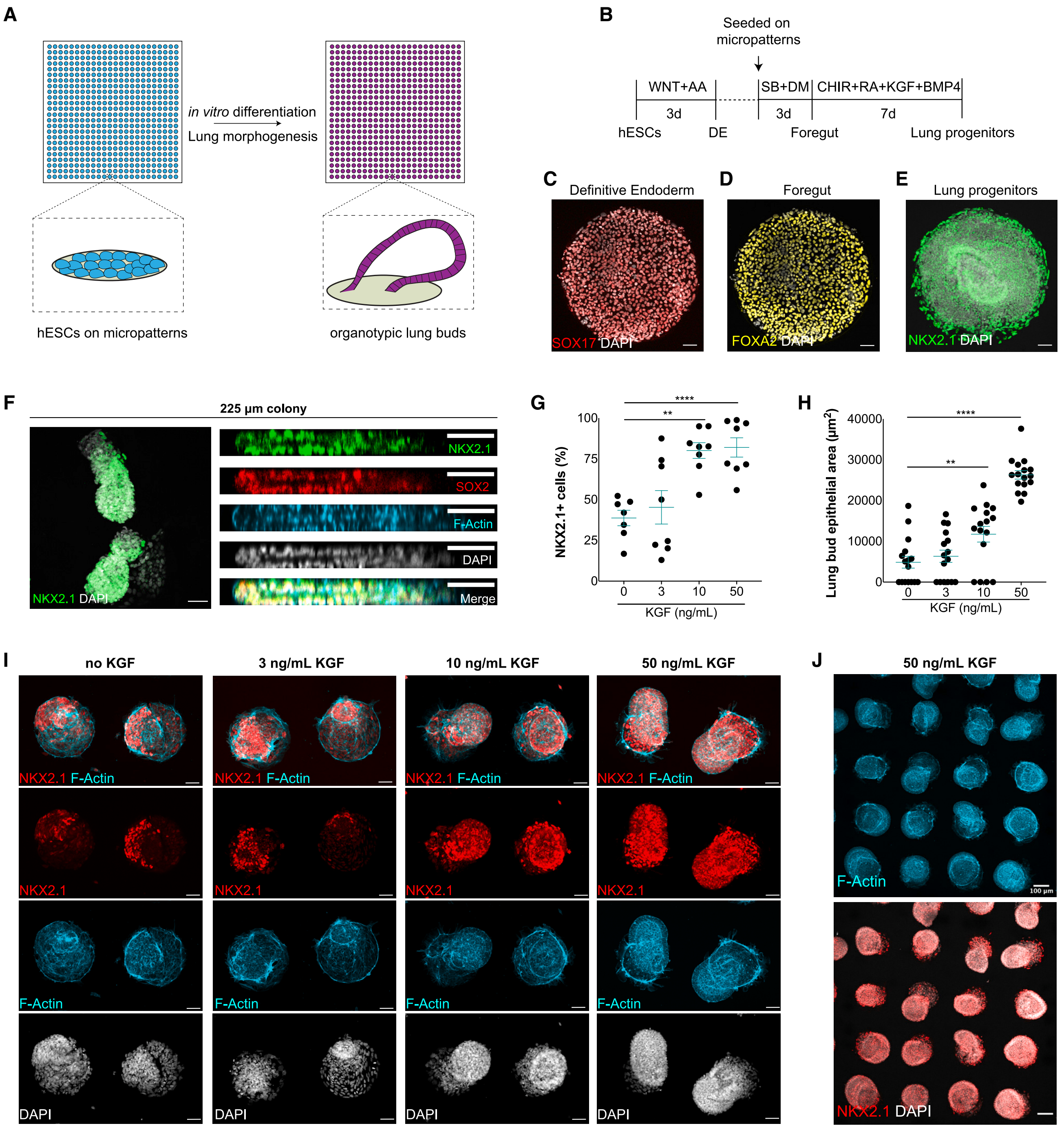

Figure 1. Generation of self-organized epithelial lung buds on confined geometries

(A) Experimental paradigm to generate self-organized lung tissue from hESCs on micropatterned colonies.

(B) Protocol for the generation of lung buds on confined geometries in micropatterns.

(C–E) Generation of SOX17+ endoderm cells (C), FOXA2+ anterior endoderm cells (D), and NKX2.1+ multipotent lung progenitors (E) at the end of definitive endoderm (DE), foregut, and lung progenitor induction stages, respectively.

(F) Top and side views of 3D epithelial buds containing NKX2.1+ multipotent lung progenitors in 225-μm colonies.

(G) Proportion of NKX2.1+ progenitor cells at increasing doses of KGF (N = 8 independent experiments, data represent mean values ± SD).

(legend continued on next page)

describe a novel lung organoid technology platform to generate thousands of nearly identical lung buds from human embryonic stem cells (hESCs). The process allows for rapid and scalable access to lung tissue, resembling human lungs in cell type and tissue complexity. The reproducible and scalable nature of these lung buds allow quantitative analysis of infection by SARS-CoV-2 and endemic coronaviruses, but also interrogate cell type-specific cytopathogenic and intercellular transmission events.

RESULTS

Reconstituting human lung development on confined geometries

Micropattern cell culture technology allows the robust and scalable generation of human organotypic tissues that model the *in vivo* embryonic counterparts in glass chips. hESCs grown in confined geometry self-organize to generate patterns of differentiated cells, modeling the early development of human organs (Warmflash et al., 2014; Haremaki et al., 2019). To overcome current limitations of existing stem cell-based models of lung development and respiratory infections, we sought to develop a micropattern-based platform to generate organotypic embryonic tissues that model fetal human lungs (Figure 1A). The generation of self-organized lung tissues relies on the stepwise modulation of signaling pathways that direct lung development *in vivo* (Figure 1B) (Jacob et al., 2017; McCauley et al., 2017; Dye et al., 2015; Miller et al., 2019; Green et al., 2011; Huang et al., 2014, 2015; Chen et al., 2017). As lung progenitor cells are derived from anterior endodermal progenitors in the embryo, we first induce hESCs to differentiate into SOX17+ definitive endoderm in standard monolayer cultures, by applying WNT and ACTIVIN stimulation for 3 days (Figures 1C, S1A, and S1B). Endodermal cells were then seeded on micropatterned substrates and exposed to the transforming growth factor β inhibitor SB431542 (SB) and BMP inhibitor Dorsomorphin (DM) for another 3 days to induce FOXA2+ anterior endoderm (foregut; Figure 1D) (Green et al., 2011; Huang et al., 2014, 2015). Finally, cells were exposed to WNT-activation, KGF, BMP4, and retinoic acid (RA) stimulation for 7 days, to promote the differentiation of NKX2.1+ multipotent lung progenitors (Figure 1E). These progenitors give rise to both airway and alveolar cell types (Morrisey and Hogan, 2010; Nikolić et al., 2017, 2018; Rawlins et al., 2009; Zepp and Morrisey, 2019). We find that compared with standard monolayer differentiation protocols where KGF is dispensable (Jacob et al., 2017; McCauley et al., 2017), a combined induction by KGF and BMP4 together, rather than each alone, led to a significant increase in the proportion of NKX2.1+ cells on lung progenitor micropatterned colonies (Figures S1C–S1F). Specification of lung progenitor fate is dependent on the levels of KGF and BMP4 signaling, as we detected a dose-dependent increase in the proportion of NKX2.1+ progenitors in micropatterned cultures (Figures 1G–1J, S1C–S1F, and S1H–S1J). This platform allows us to generate thousands of self-organizing lung progenitor colonies of defined sizes in a single micropattern chip (Figures S1G and S1H).

During early human development *in vivo*, fetal lung progenitors arise in epithelial buds that form from an outpouching of the anterior endoderm, co-expressing NKX2.1 and SOX2, around week 4 of gestation (Morrisey and Hogan, 2010; Nikolić et al., 2017, 2018; Rawlins et al., 2009; Zepp and Morrisey, 2019). Later, NKX2.1 expression becomes highly enriched in the alveoli, while SOX2 expression specifically demarcates the airway (Morrisey and Hogan, 2010; Nikolić et al., 2017, 2018; Rawlins et al., 2009; Zepp and Morrisey, 2019). Interestingly, we find that upon induction, multipotent lung progenitors self-organize into three-dimensional (3D) epithelial chords containing NKX2.1+/SOX2+ progenitors (Figures 1F and S1G), reminiscent of fetal lung buds *in vivo*. The number of individual buds increased with colony size and was restricted to a single individual bud in 225-μm-diameter colonies (Figures 1F and S1G). We find a dose-dependent increase in the epithelial area of lung buds upon KGF modulation (Figures 1G–1I and S1H). High doses of KGF (50 ng/mL) robustly induce epithelial lung buds that are commensurate in size and morphology (Figures 1J and S1H). This model highlights the self-organizing capabilities of stem cell-derived lung progenitors on confined circular geometries with *in vivo*-like morphogenetic features.

Proximodistal patterning of fetal-like human lung buds

Upon induction of the lung primordium, epithelial buds are further patterned along their proximodistal axis, which leads to a coordinated segregation of proximal SOX2+ airway and distal NKX2.1+/SOX9+ alveolar progenitors (Figures 2A, 2B, and 2F) (Morrisey and Hogan, 2010; Nikolić et al., 2017, 2018; Rawlins et al., 2009; Zepp and

(H) Quantification of lung bud epithelial area in micropattern colonies at varying doses of KGF (N = 14 independent experiments, data represent mean values ± SD).
(I) Micropattern colonies containing NKX2.1+ epithelial buds at increasing doses of KGF.
(J) Low-magnification images of epithelial buds containing NK×2.1+ lung progenitors grown on confined geometries of 225-μm diameter at high doses of KGF (50 ng/mL) (**$p < 0.01$,***$p < 0.001$, ****$p < 0.0001$, Dunnett's multiple comparison test; scale bar, 50 μm).

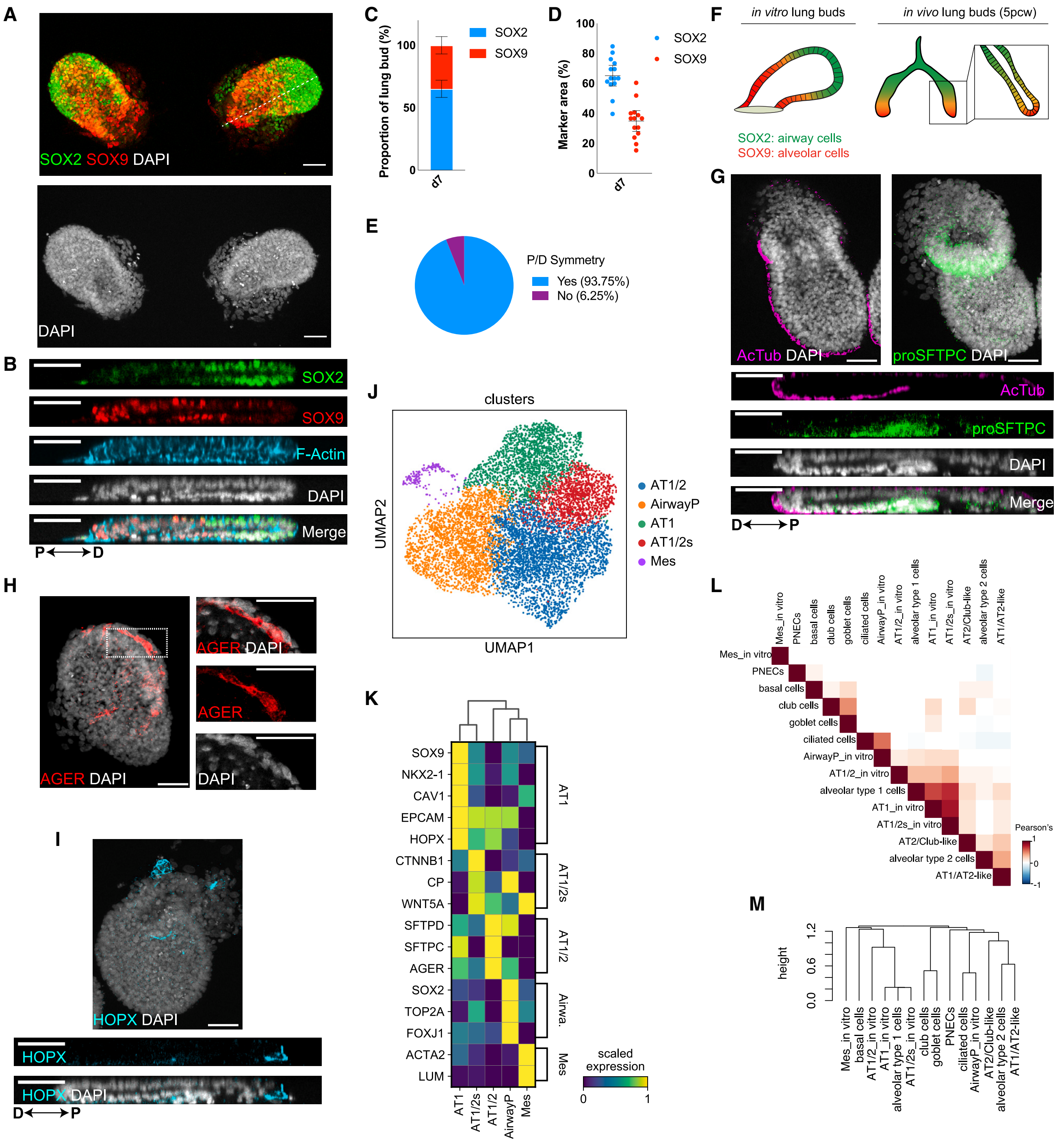

Figure 2. Proximodistal coordination of airway and alveolar tissue differentiation in human lung buds

(A and B) Top (A) and side (B) views of lung buds with proximodistal segregation of SOX2+ airway and SOX9+ alveolar progenitors.
(C) Proportion (C) and percentage area (D) of SOX9 and SOX2 expression domains in lung buds (N = 3 independent experiments, data represent mean values ± SD).
(E) Percentages of lung buds displaying proximodistal organization of airway and alveolar tissue (N = 3 independent experiments).
(F) Diagram of lung buds on micropatterns *in vitro* (C) or in human embryos at post-conception week (pcw) 5 *in vivo*.
(G–I) Top view and side view of lung buds and identification of multi-ciliated (AcTub+, G), type 2 pneumocytes (proSFTPC+, G), type $^{1}/_{2}$ pneumocytes (HOPX+, I), and type 1 pneumocytes (AGER+, H).

(legend continued on next page)

Morrisey, 2019). This initial patterning event is critical for the coordination of region-specific tissue morphogenesis and the differentiation of airway and alveolar cells that will ensue. These features are not faithfully and robustly recapitulated in current lung organoid protocols in a reproducible manner (Nikolić et al., 2018). Strikingly, lung progenitors grown on small, confined geometries (225-μm diameter) display proximodistal coordination of progenitor differentiation with SOX9+ alveolar-like and SOX2+ airway-like cells located in non-overlapping tissue domains (Figures 2A and 2B), modeling human embryonic fetal lung bud development around week 5 of gestation (Morrisey and Hogan, 2010; Nikolić et al., 2018). SOX9+ alveolar-like progenitor cells are positioned proximal to the micropattern surface, whereas SOX2+ airway-like progenitor cells are located more distally forming a continuous 3D epithelial structure (Figures 2A and 2B). The proximodistal patterning and *in vivo*-like segregation of airway and alveolar progenitors was observed in 94% of micropatterned colonies with highly reproducible marker expression domains (Figures 2C–2E). These human lung buds also display early hallmarks of cellular differentiation and express markers of airway multi-ciliated cells (AcTub+; Figure 2G) and basal stem cells (Figure S1L), as well as alveolar type 1 (AGER+, HOPX+, Figure 2H-1) and type 2 pneumocytes (proSFTPC+, HOPX+, Figures 2G and 2I). Type 1 and type 2-like pneumocytes are located proximal to the micropattern surface and airway multi-ciliated cells are in the EPCAM+ epithelium forming in the distal tip of the lung bud (Figures 2G–2I and S1K–S1M). In 500-μm lung progenitor colonies, we also detected airway basal stem cells (P63+) and mucus-producing goblet cells (MUC5AC+) in epithelial cords that were less organized compared with 225-μm micropatterns, presumably due to size-dependent tissue morphogenesis imposed by geometric confinement (Figures S1N–S1R). Thus, lung progenitor cells cultured on confined geometries self-organize into fetal-like human lung buds with proximal-distal coordination of alveolar and airway tissue differentiation.

Molecular signature of standardized human lungs

To further characterize cell types present in self-organizing human organotypic lungs, we performed single-cell RNA-sequencing (scRNA-seq) analysis at day 7 of lung differentiation in 225-μm circular micropattern colonies. Using cell type-specific markers of lung cells, previously identified in scRNA-seq analysis of primary human lungs (Travaglini et al., 2020; Wang et al., 2020), five major cell subpopulations were identified. These consist of (1) SOX2+ airway progenitors (AirwayP), (2) alveolar progenitor cells (AT1/2), (3) cycling alveolar progenitor cells (AT1/2s), (4) early type 1 pneumocytes (AT1), and (5) mesenchymal (Mes) cells (Figures 2J, 2K, and S2A–S2E). SOX2+ airway cycling progenitors can be identified based on the enriched expression of SOX2 and FOXJ1 and TOP2A, a cell cycle marker (Figures 2K, S2D, and S2E). Early progenitors AT1/2 cells co-expressed the AT1 marker AGER as well as surfactant proteins SFTPD, SFTPC, and SFTPB (Figures 2K, S2E, S2G, and S2H). AT1 cells display high levels of SOX9 as well as the AT1 markers CAV1 and HOPX (Figures 2K, S2G, and S2H). AT1/2s display high levels of the canonical AT2 marker ETV5, as well as CTNNB1, CP, WNT5A, and TCF7L2 (Figures 2K, S2G, and S2H), which are marker genes of progenitor-like alveolar cells (Travaglini et al., 2020). Compared with other alveolar cell subpopulations, AT1/2s cells express high levels of the cell cycle marker TOP2A, suggesting that these cells correspond to stem cell-like cycling alveolar cells (Figures S2G and S2H). The identity of mesenchymal-like cells (Mes) is based on the expression of lumican (LUM), collagen (COL3A1, COL1A2), and ACTA2, a marker of lung mesenchyme (Figures 2K, S2D, and S2E) (Danopoulos et al., 2018, 2020).

We hypothesize that alveolar cells present in lung buds correspond to a fetal immature state. Although the expression of key markers of alveolar cells, such as SFTPC, SFTPB, HOPX, PDPN, AGER, SPOCL2, and ETV5, is detected in these cells (Figures S2F–S2H), markers of mature alveolar cells, such as ABCA3 (critical for lamellar body formation), LAMP3, and NAPSA, are not expressed at levels detected by scRNA-seq. Airway cells likely correspond to multi-ciliated cells as they expressed key markers of this cell type including SOX2, FOXJ1, and TPPP3 (Figures 2K and S2D–S2F). We did not detect high expression levels of intestinal (CDX2), liver (HHEX), or thyroid (PAX8) markers in lung buds based on our scRNA-seq analysis, suggesting that lung bud cells are not hepatic, thyroid, or intestinal-like (Figure S2F).

To ascertain whether lung buds display conserved gene expression with *in vivo* counterparts, we performed canonical correlation analysis and data integration (Stuart et al., 2019) to align our dataset to a published dataset of fetal (30 weeks), juvenile (3 years), and adult human lungs (30

(J) UMAP plot and identification of five major cell clusters in lung buds by single-cell RNA-sequencing.
(K) Heatmap of scaled gene expression levels in each of the identified cell types of cell type-specific lung markers of alveolar AT1 (CAV1+), AT1/2 (AGER+,SFTPD+), and AT1/2s (CTNNB1+), airway progenitors (SOX2+, FOXJ1+, TOP2A+) and mesenchymal cells (ACTA2+, LUM+).
(L and M) Cluster-level gene expression Pearson's correlation analysis and hierarchical clustering of cell types identified in *in vitro*-derived lung buds and in adult lung tissue. Scale bars, 50 μm. D, distal; Mes, mesenchyme; P, proximal; proSFTPC, pro-surfactant protein C; SOX2+ Prog, SOX2+ progenitors; SOX9+ Prog, SOX9+ progenitors.

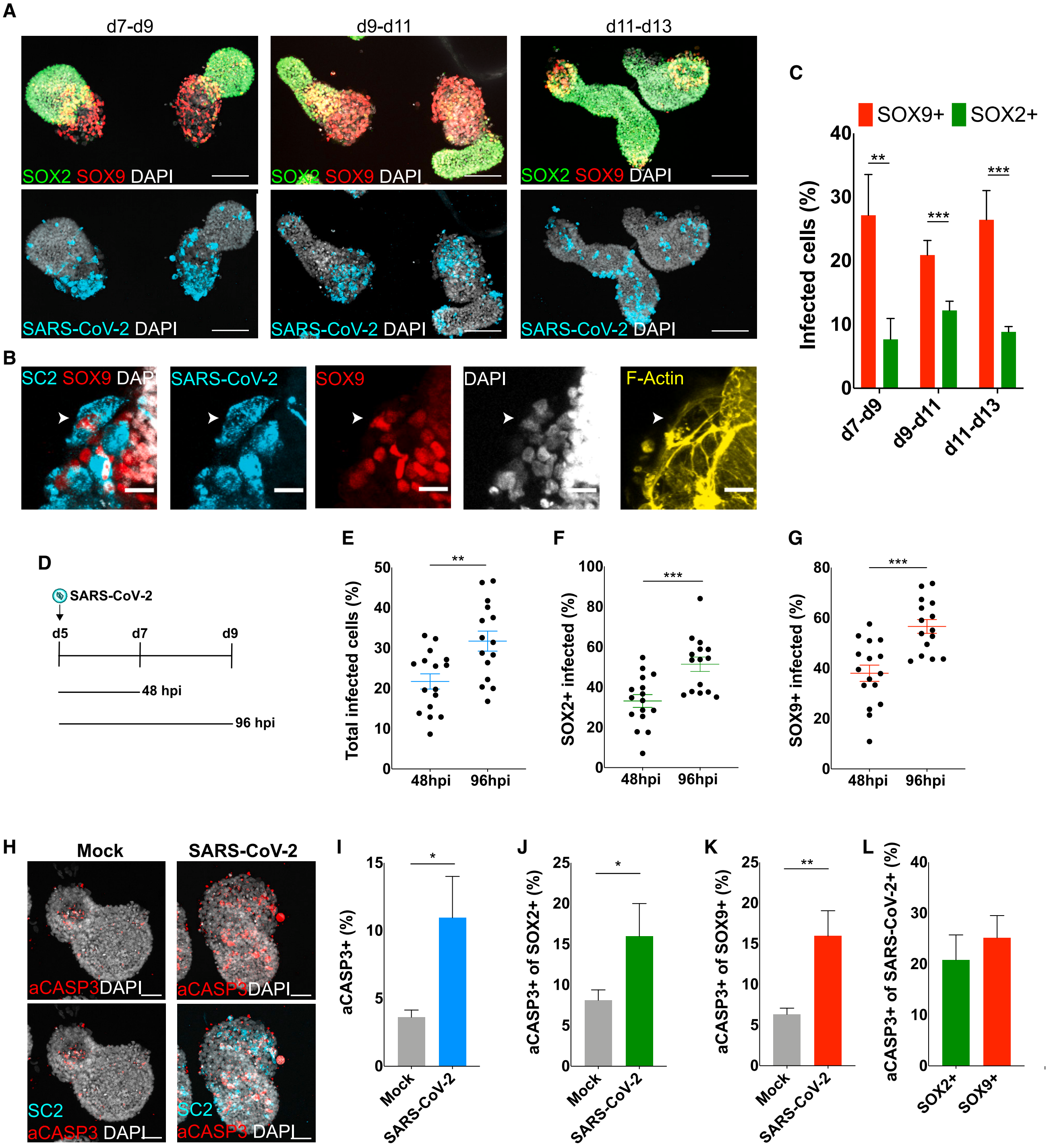

Figure 3. SARS-CoV-2 infect alveolar and airway tissue in human lung buds

(A) Lung buds infected with SARS-CoV-2 at day 7, 9, or 11 of lung bud formation and collected 48 h post-infection (hpi) (scale bar, 100 μm).

(B) SARS-CoV-2 infection in SOX9+ alveolar cells (scale bar, 50 μm).

(C) Percentage of infected cells in SOX9+ alveolar and SOX2+ airway cells (N = 9 independent experiments, data represent mean values ± SD).

(D) Experimental scheme to track SARS-CoV-2 transmission in lung buds.

(legend continued on next page)

years) (Wang et al., 2020). Cluster-level correlation analysis, based on the average expression value for each gene in each cell type, unveiled high correlation between alveolar-like cells AT1/2, AT1/2s, and AT1 in *in vitro* lung buds with AT1 of primary human lung tissue (Figure 2L), with the highest correlation observed between AT1 cells of primary human lung tissue and lung buds (Figure 2L). These cells also displayed positive correlation with AT2/Club-like and AT1/AT2-like cells *in vivo,* possibly due to their progenitor-like phenotypic signature (Wang et al., 2020). Moreover, AirwayP cells in lung buds displayed high correlation with ciliated cells in primary human lung tissue (Figure 2L). We observed positive correlation (correlation coefficient >0.05) with primary alveolar and airway across all developmental stages analyzed with no clear stage-specific correlation signature (Figures S2I–S2K). Hierarchical clustering based on cluster-level correlations between *in vitro* and *in vivo* datasets further identified two clusters containing both *in vitro*-derived and *in vivo* lung tissue: one with AT1 and AT1/2 alveolar cells and one with airway ciliated cells (Figure 2M). Thus, lung buds display conserved cell types and convergent gene expression with human lung tissue *in vivo*.

Tracking viral infection and transmission in human lung buds

As these human lung buds contain cellular targets of SARS-CoV-2 and endemic coronaviruses, we sought to determine whether we can track SARS-CoV-2 infection and pathology in these tissues. We hypothesized that organotypic human lung buds could be infected by various coronaviruses as we detected the expression of coronavirus receptors ACE2 and ANPEP as well as associated proteases FURIN and TMPRSS2 in multiple cell types (Figures S3A–S3C). To test lung bud susceptibility, we infected the lung buds at day 7 with the betacoronavirus HCoV-OC43 and alphacoronaviruses, HCoV-229E and HCoV-NL63. High levels of infection were observed for the endemic coronaviruses HCoV-229E, HCoV-OC43, and, to a lower extent, HCoV-NL63, suggesting that *in vitro* stem cell-derived human lung buds are a tractable model to study respiratory infection by human coronaviruses (Figures S3D–S3G).

To determine if this platform recapitulates COVID-19 pathology, we infected lung buds at different stages of lung bud formation with a patient-derived isolate of SARS-CoV-2 (USA-WA1/2020; Figures 3A–3C and S3H–S3J). We detected viral infection in both SOX2+ airway and SOX9+ alveolar cells (Figures 3A–3C and S3H–S3J). In contrast to experiments with primary lung tissue cultured *in vitro* (Hou et al., 2020), we identified an increased susceptibility to infection in alveolar cells compared with airway cells (Figures 3C and S3H–S3J). Consistent with these results, we detected higher expression of the receptor ACE2 as well as proteases TMPRSS2 and FURIN, which regulate SARS-CoV-2 entry into target cell types (Shang et al., 2020; Stuart et al., 2019; Walls et al., 2020), in alveolar cell types compared with airway cells (Figures S3A–S3C).

To assess whether the virus could spread to uninfected lung bud cells, infection was tracked for 48 and 96 h post-inoculation (Figure 3D). Notably, there was a marked increase in the number of infected cells over time in a cell type-independent manner (Figures 3E–3G), suggestive of viral spread in human lung buds. We also sought to determine whether lung buds recapitulate cytopathic effects upon infection. Apoptosis is recognized as an important host antiviral defense mechanism that controls viral infection and regulates inflammatory responses (Ren et al., 2020). Upon infection by SARS-CoV-2, cellular apoptosis is induced by both host antiviral response and accessory viral proteins such as ORF3a (Ren et al., 2020). Infected cells displayed high, yet similar, levels of apoptosis in both alveolar and airway tissues compared with mock lung buds mimicking virus-induced pathology (Figures 3H–3L). This platform recapitulates the complete viral life cycle while also enabling tracking of cell type-dependent susceptibilities to infection, intercellular transmission, and cytopathic effects in lung buds.

Intriguingly, inspection of SARS-CoV-2-infected alveolar and airway tissue identified SFPTC+ alveolar cells and to a lower extent AcTub+ multi-ciliated cells as targets of SARS-CoV-2 in lung buds (Figures 4A and 4B), corroborating *in vivo* analyses of infected tissues from patients (Hou et al., 2020). The data also identify type 1/2 pneumocytes (HOPX+) as additional targets of SARS-CoV-2 (Figure 4C), consistent with their high levels of expression of ACE2 receptor and SARS-CoV-2-activating proteases (Figures S3A–S3C), as in adult human tissue (Ziegler et al., 2020). Surprisingly, SARS-CoV-2 infection was also observed in phospho-Histone3 (pH3)-positive cycling SOX9+ alveolar progenitor cells, which correspond to cycling AT1/2s (Figures 4D–4F). These cells also display high expression of ACE2 and virus-activating proteases (Figures S3A–S3C). However, there were no significant differences in the levels of infection between dividing and non-dividing SOX9+ alveolar cells

(E–G) Percentage of infected cells of total, SOX2+ and SOX9+ cells 48 hpi and 96 hpi (N = 9 independent experiments, data represent mean values ± SD).
(H–L) Detection and quantification of active CASP3+ apoptotic cells in mock and SARS-CoV-2 infected lung buds (scale bar, 50 μm; N = 9 independent experiments, data represent mean values ± SD). *p < 0.05, **p < 0.01,***p < 0.001, ****p < 0.0001, Dunnett's multiple comparison test. aCASP3, active Caspase-3; D, distal; P, proximal; SC2: SARS-CoV-2.

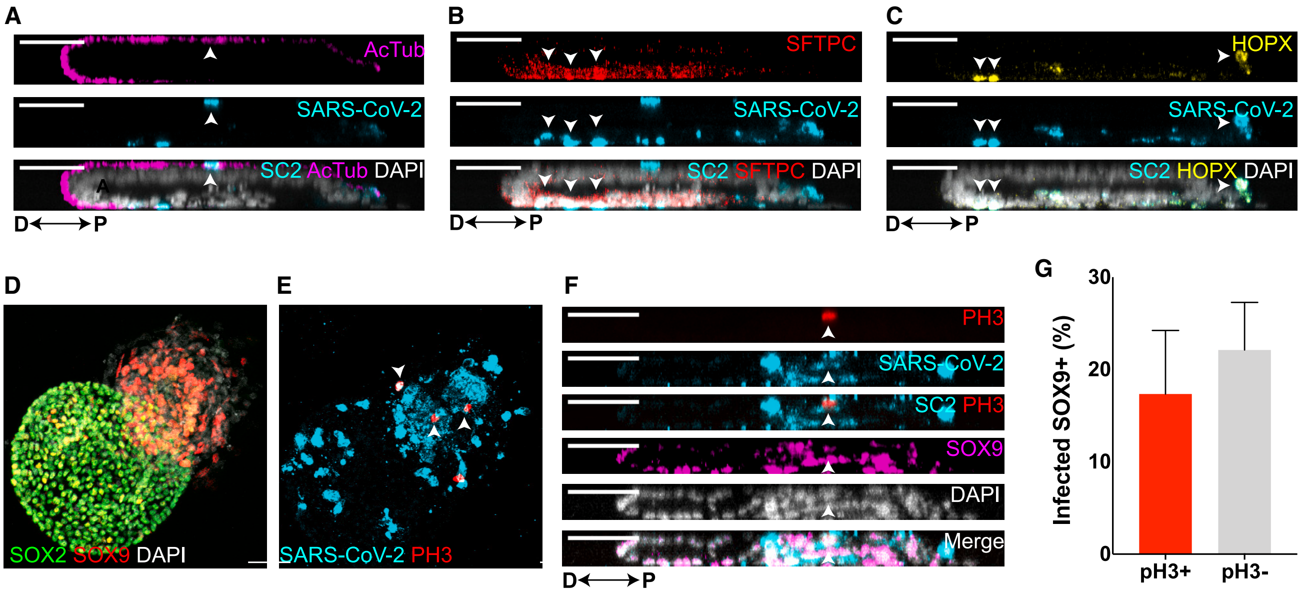

Figure 4. Cycling alveolar stem cells are targets of SARS-CoV-2

(A–C) Detection of SARS-CoV-2 infection in multi-ciliated (AcTub+; A), type 1/2 pneumocytes (proSFTPC+, HOPX+; B and C). Arrows denote infected cells expressing cell type-specific markers (scale bar, 50 μm).

(D–G) Detection and quantification of SARS-CoV-2-infected pH3+ cycling SOX9+ alveolar cells. Arrows denote infected pH3+ cycling cells (N = 9 independent experiments, data represent mean values ± SD). Scale bar, 50 μm. D, distal; P, proximal; PH3: phospho-Histone H3; SC2: SARS-CoV-2.

(Figure 4G), suggesting that cycling stem cell-like alveolar cells are highly susceptible to infection at levels similar to non-dividing alveolar cell types.

Human lung buds for comparative therapeutic screens

As this platform mimics *in vivo* features of lung tissue and is scalable, we next tested for the ability to screen therapeutics that were shown to limit SARS-CoV-2 infection through binding assays and in cell lines (Robbiani et al., 2020). We developed a 96- and 384-well plate-based assay for high-throughput-based characterization and screening of COVID-19 therapeutics (Figure 5A). Using this high-throughput assay, we were able to assess dose-dependent infection in a highly robust and quantitative manner (Figure 5B). Human antibodies isolated from convalescent plasma of patients (Robbiani et al., 2020) were tested for their efficiency in neutralizing SARS-CoV-2 and preventing infection (Figure 5A). Each neutralizing antibody (nAb) was tested at a range of dilutions (Figures 5C–5E). Each nAb potently inhibited SARS-CoV-2 infection (Figures 5C–5E) at half maximal inhibitory concentrations similar to those found for cell lines (Robbiani et al., 2020). As expected, the nAbs broadly inhibit SARS-CoV-2 infection, rather than in a cell type-specific manner (Figures 5F and 5G). Although the initial assay shows that nAbs can inhibit infection when incubated with the virus prior to infection, we next wanted to mimic more pragmatic therapeutic approaches, whereby the nAb is administered once the patient has already been infected by the virus to inhibit viral spread (Figure 5H). We find that nAbs can inhibit SARS-CoV-2 spread throughout the organoid, when compared with an nAb control (Goo et al., 2019), highlighting the utility of this system to mimic *in vivo* situations (Figure 5I) and to test candidate COVID-19 therapeutics in a high-throughput manner.

SARS-CoV-2 infection-associated lung-specific gene expression signatures

To further identify cellular responses to infection, we explored gene expression programs associated with SARS-CoV-2 infection. Time course RNA-seq analysis of infected lung buds was performed at 12, 24, and 48 hours post-infection (hpi) (Figures 6A and 6B). Principal-component analysis (PCA) of RNA-seq data of infected lung buds collected at multiple stages post-infection showed a time-dependent association of gene expression along the first principal component (PC1) with 58% of the variance explained (Figure 6A). For each time point, differentially expressed genes (DEGs) were compared with mock stage-matched tissue (Figure 6B). As observed in patient samples, there was marked increase in genes representative of pro-inflammatory and type I interferon response pathways, such as TNFAIP3, NFKBIA, STAT1, and IRF1, among others, as early as 12 hpi, and increasing at 48 hpi (Figure 6B). Consistently, further gene set enrichment analysis (GSEA)

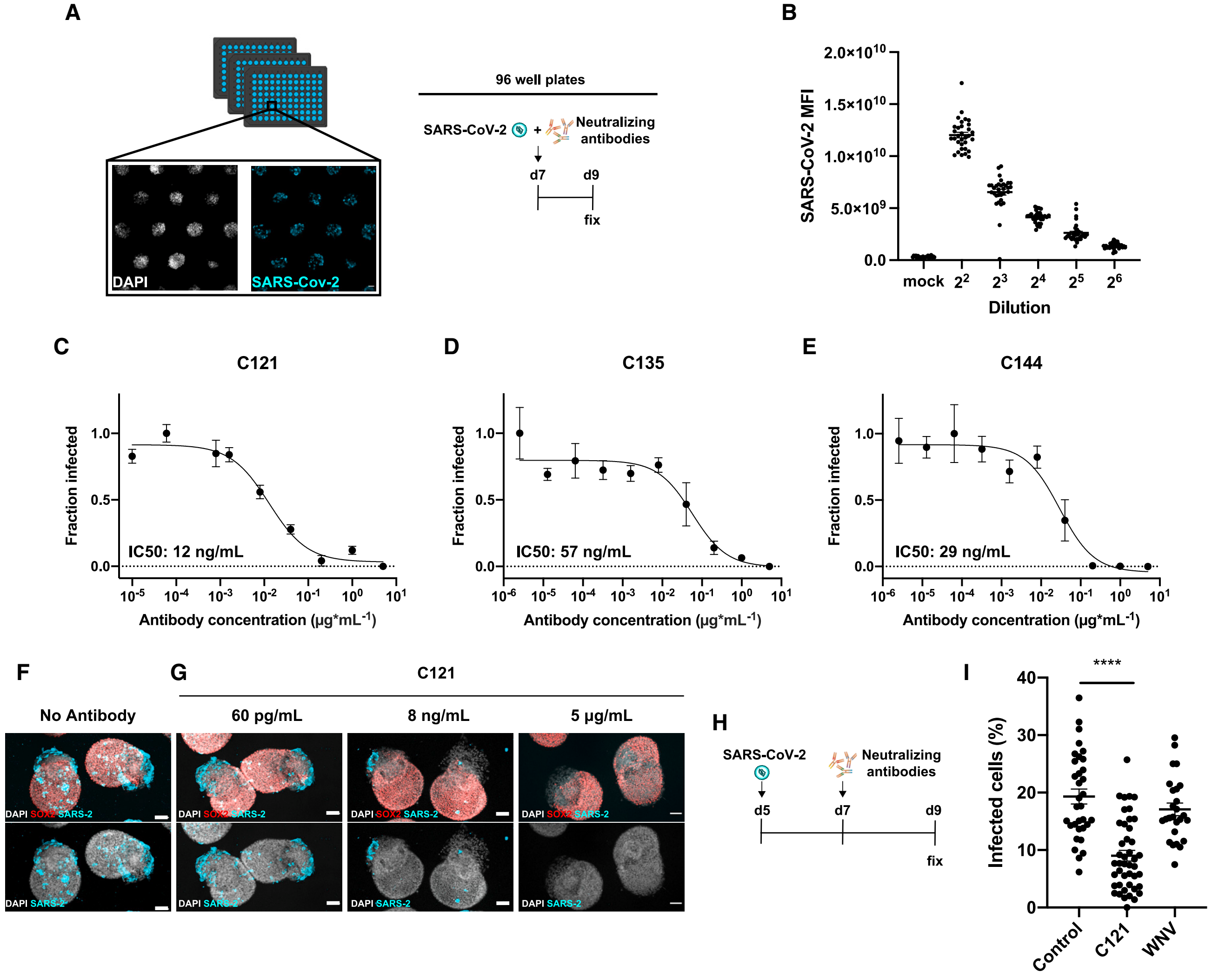

Figure 5. Evaluation of antibodies that neutralize SARS-CoV-2 infection

(A) Experimental scheme for the high-throughput-based testing of SARS-CoV-2-neutralizing antibodies in 96-well plates.

(B) Quantification of dose-dependent SARS-CoV-2 infection in the high-throughput micropattern lung bud platform (N = 16 independent experiments, data represent mean values ± SD).

(C–E) Neutralization curves of SARS-CoV-2 infection in lung buds at increasing doses of antibodies. Data normalized to maximum (1) and minimum (0) infection levels (N = 3 independent experiments, data represent mean values ± SD).

(F and G) Representative images of lungs infected with SARS-CoV-2 and treated with varying doses of C121 human antibody (scale bar, 50 μm).

(H) Experimental scheme to test the effect on neutralizing antibodies on intercellular transmission. Lung buds were infected with SARS-CoV-2 at day 5 of lung bud formation and treated with neutralizing antibodies 48 h later.

(I) Proportion of infected cells in human lungs treated with C121 or a West Nile virus (WNV)-specific antibody from day 7 to day 9 (N = 3 independent experiments, data represent mean values ± SD). ****$p < 0.0001$, Dunnett's multiple comparison test. MFI, mean fluorescence intensity.

of genes identified an enrichment of gene ontology terms related to regulation of viral process, cellular responses to viral infection, as well as cytokine production and cellular responses to type 1 interferons (Figure 6C).

Since a large fraction of the gene expression changes observed have been associated with responses to viral infection, we sought to identify SARS-CoV-2-specific gene expression programs. To do that, we collected RNA-seq data of lung buds infected with endemic coronaviruses OC43 or 229E (Figures S4A–S4C). We observed a divergent antiviral gene expression response to infection by OC43 and 229E compared

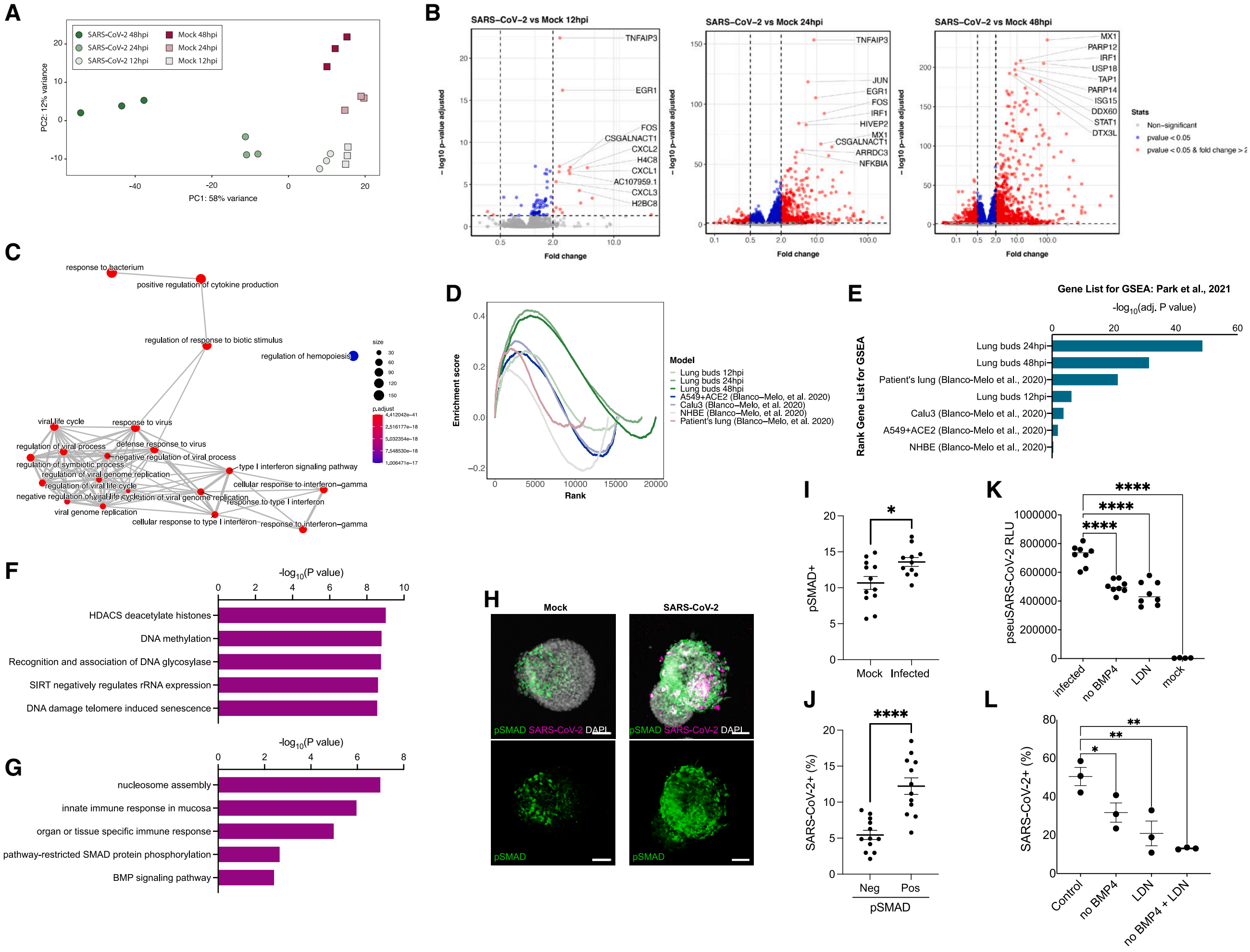

Figure 6. BMP signaling is induced in infected lung tissue and regulates SARS-CoV-2 infection

(A) Volcano plots of DEG genes of SARS-CoV-2 infection compared with mock lung buds 12, 24, and 48 hpi.

(B) PCA analysis of gene expression of SARS-CoV-2-infected and mock lung buds 12, 24, and 48 hpi.

(C) Network plot of the top 20 enriched gene ontology terms based on the similarity of the genes they contain. Gene ontology terms were identified from GSEA analysis of genes ranked based on their contribution to PC1.

(D and E) GSEA enrichment scores (D) and adjusted p values for the enrichment (E) of patient-associated COVID-19 gene expression signatures (Park et al., 2022) in lung buds and other *in vitro* models.

(F and G) Overrepresentation analysis of lung-specific SARS-CoV-2-associated upregulated genes from 9D) based on gene ontology biological process (F) or Molecular Signatures Database (MSigDB) C2 categories (G).

(H) pSMAD1/5 expression in SARS-CoV-2-infected and mock lung buds 48 hpi.

(I) Proportion of pSMAD1/5+ cells in SARS-CoV-2-infected and mock lung buds 48 hpi (N = 9 independent experiments, data represent mean values ± SD). Scale bar, 100 μm.

(J) Proportion of SARS-CoV-2-infected cells in pSMAD-negative or positive cells 48 hpi (N = 9 independent experiments, data represent mean values ± SD).

(K) Quantification of viral entry by pseudotyped SARS-CoV-2 upon modulation of BMP pathway 48 hpi (N = 9 independent experiments, data represent mean values ± SD).

(L) Proportion of SARS-CoV-2+ infected cells upon BMP pathway modulation 48 hpi (N = 3 independent experiments, data represent mean values ± SD). *p < 0.05, **p < 0.01, ****p < 0.0001, Dunnett's multiple comparison test. RLU, relative luciferase units.

with SARS-CoV-2 based on PCA (Figure S4A). Among DEG genes (log2 fold change >2 and adjusted p value <0.05), we found that 1,678 genes are specific to SARS-CoV-2 infection (Figures S4B and S4C). Our analysis highlights both divergent and convergent cellular responses to infection by coronaviruses in lung tissues.

Organotypic self-organizing lung buds provide a paradigm to model physiological responses to SARS-CoV-2 infection. GSEA analysis of our dataset with gene sets previously associated with SARS-CoV-2 infection in cancer cell lines, bronchial epithelial cells, and tissues from COVID-19 patients (Blanco-Melo et al., 2020), highlighted convergent gene expression programs across these models based on enrichment analysis (Figure S4D). To further identify gene expression responses to SARS-CoV-2 infection that are specific to infected lung tissue, we compared our dataset with a published dataset of postmortem tissues from COVID-19 patients (Park et al., 2022). DEGs (adjusted p value <0.05) were identified for SARS-CoV-2-infected lung buds (this study), lung postmortem tissue (Park et al., 2022), and the cancer cell lines A549 and Calu3 (Blanco-Melo et al., 2020). GSEA rank analysis of DEGs (adjusted p value <0.05) in COVID-19 lung postmortem tissue identified a significant enrichment of COVID-19-associated gene signatures in infected lung buds (Figures 6D and 6E). We further detected an increased enrichment of the patient-associated gene signatures in lung buds compared with other *in vitro* models, including cancer cell lines A549 and Calu3 (Figures 6D and 6E). Moreover, we identified 145 genes that were differentially expressed in infected lung buds and postmortem lung tissue, but not in the cell lines (Figure S4E), suggesting that these genes are lung-specific. Among those genes, we found an enrichment of pathways related to DNA methylation, DNA damage-induced senescence, immune response, and importantly, BMP signaling (Figures 6F and 6G). This analysis highlights the utility of human lung buds to identify lung-specific gene expression responses to infection by SARS-CoV-2.

BMP signaling tunes SARS-CoV-2 infection

BMP signaling plays a salient role in tissue remodeling upon lung injury (Cassandras et al., 2020), yet whether it regulates tissue responses to viral infection is unknown. Interestingly, multiple factors involved in the BMP pathway were identified as essential pan-coronavirus host factors in a CRISPR-Cas9 screen that included SARS-CoV-2 (Schneider et al., 2021). As this pathway is also a lung-specific SARS-CoV-2-associated pathway (Figure 6G), we hypothesized it would play an important role in dictating susceptibility to SARS-CoV-2 infection in lung tissue. To test this, we stained SARS-CoV-2-infected lung buds for pSMAD1/5, a marker of active BMP signaling. Interestingly, infected lung buds display an increase in the proportion of cells expressing pSMAD1/5 (Figures 6H and 6I). Furthermore, in these SARS-CoV-2-infected lung buds, pSMAD1/5-positive cells were 2.2-fold more likely to be infected by SARS-CoV-2 (Figure 6J), suggesting that active BMP renders lung cells more susceptible to infection by SARS-CoV-2. Next, to functionally test whether BMP signaling regulates infection, we performed infection experiments with pseudoviruses or native SARS-CoV-2 in the absence of BMP4 and/or with the BMP inhibitor, LDN193189. To do that, we removed BMP or added LDN at day 5 of lung bud formation concomitantly with virus infection and collected infected tissues 48 h post-infection. Intriguingly, we found that pharmacological inhibition of BMP or absence of pathway stimulation by BMP4 leads to a reduction in SARS-CoV-2 infection (Figures 6K and 6L). We did not observe differences in the proportion of alveolar and airway cells nor in the epithelial area of lung buds upon inhibition of BMP by LDN and/or removal of BMP during the treatment window (Figures S5A–S5D), which suggests that the effect of BMP on SARS-CoV-2 infection is independent from its effects on differentiation. As the antiviral effect of BMP signaling inhibition was observed in a pseudovirus assay that measures viral entry, our experiments suggest that BMP signaling plays a role in SARS-CoV-2 entry in lung cells. In agreement with this hypothesis, we observed a decreased expression of ACE2 in lung buds upon inhibition or removal of BMP (Figures S5E and S5F). Thus, BMP activity in lung cells may influence susceptibility to SARS-CoV-2 infection by regulating ACE2 expression.

DISCUSSION

This work highlights the self-organizing capabilities of lung progenitors when cultured on confined geometries. The human lung bud model recapitulates the earliest events in fetal lung development, which involves the proximodistal coordination of alveolar and airway cellular differentiation and region-specific tissue morphogenetic events (Morrisey and Hogan, 2010; Nikolić et al., 2017, 2018; Rawlins et al., 2009; Zepp and Morrisey, 2019). It also offers key advantages to current *in vitro* models, which includes the standardization of *in vivo*-like tissue organization and complexity, and inexhaustible access to disease-relevant lung tissue. This platform will allow for the interrogation of the molecular and genetic mechanisms orchestrating early human lung differentiation and morphogenesis, in both normal and disease states in which these processes may go awry, such as lung cancer and pulmonary diseases. This platform also provides fast and scalable access to lung tissue for regenerative medicine and modeling lung diseases.

Compared with other lung organoid protocols that display high inter-organoid and batch-to-batch variability (Nikolić et al., 2018), this platform allows for the individual tracking of many lung buds at a time in a highly standardized manner. These lung buds reproducibly self-organize

into epithelial structures that display morphogenetic similarities to developing lungs in human embryos (Morrisey and Hogan, 2010; Nikolić et al., 2017, 2018; Rawlins et al., 2009; Zepp and Morrisey, 2019). In particular, lung buds display proximodistal patterning of alveolar and airway tissue reminiscent of fetal counterparts *in vivo* at around week 5 of gestation (Morrisey and Hogan, 2010; Nikolić et al., 2017, 2018; Rawlins et al., 2009; Zepp and Morrisey, 2019). We have demonstrated the utility of lung buds to define a molecular logic during early lung development, including the synergy of multiple signaling pathways for the differentiation and self-organization of multipotent lung progenitors. In particular, we define a salient role of KGF for the self-organization of developing epithelial structures in fetal-like human lung buds, as observed in *in vivo* models (Yano et al., 2000). Although other organoid protocols allow for the generation of tissues that resemble lungs *in vivo*, they lack a controlled self-organization of lung progenitors (Jacob et al., 2017; McCauley et al., 2017; Dye et al., 2015; Miller et al., 2019; Green et al., 2011; Huang et al., 2014, 2015; Chen et al., 2017). Moreover, inter-organoid phenotypic variability and batch-to-batch differences limit their compatibility for the study of genetic determinants of cell fates and how they choreograph tissue morphogenetic events. Thus, human lung buds offer a new paradigm to study early human lung development and human-specific lung biology that would otherwise be impossible to scrutinize.

We highlight the potential of using lung buds to identify novel therapeutics that block infection by SARS-CoV-2, endemic coronaviruses, as well as other respiratory viruses. Compared with 3D organoid models in which tracking virus-induced cellular responses is challenging (Han et al., 2020), this platform allows for the individual tracking of many genetically matched reproducible organoids at a time to gain a quantitative understanding of SARS-CoV-2-induced lung pathology. We have established a highly quantitative platform to assess differential cell type-dependent susceptibilities to infection and cytopathology, as well as to identify key target cell types that are not accessible for experimentation *in vivo*. Notably, we have also identified cycling alveolar cells as targets of SARS-CoV-2, which display tropism for this virus at levels that are similar to non-dividing alveolar cell types. These findings have important clinical implications for COVID-19 as stem cell-like AT2 cells, which display shared key markers with AT1/2s in lung buds, are proposed to be signaling hubs for tissue homeostasis and regeneration in alveoli (Travaglini et al., 2020), and tissue damage in COVID-19 patients may result from the loss of these cells. As intrinsic immunity dictates viral tropism and susceptibility to infection of stem cells (Wu et al., 2018), we highlight potentially novel mechanisms of viral susceptibility in lung tissue. Whether this lung model recapitulates infection in fetal and adult tissue *in vivo* warrants further investigation.

Finally, this platform will help gain an understanding of the cellular mechanisms that direct SARS-CoV-2-induced pathogenesis. Importantly, as this platform is amenable for high-throughput genetic analysis, it will also allow for the discovery of cellular factors that control lung infection, which will identify novel therapeutic targets for COVID-19. Of note, we have demonstrated the utility of lung buds to identify lung-specific gene expression hallmarks of SARS-CoV-2 infection, which led to the identification of BMP signaling as a critical regulator of infection in the lung. Further evidence for the role of BMP signaling in entry is due to the identification of BMP as a host factor in pooled screens that naturally bias toward factors related to viral entry (Schneider et al., 2021). Future studies may use this platform for rapid and scalable genome-wide screening for essential host factors (Hoffmann et al., 2021; Schneider et al., 2021) and small molecule screens. Finally, this model will also help delineate how patient mutations may modify disease progression in respiratory lung diseases such as COVID-19 (Zhang et al., 2020), and provides the hope to have a transformative influence in the elucidation of the cellular and molecular basis of respiratory infections and lung diseases for which there are currently no therapies.

EXPERIMENTAL PROCEDURES

Resource availability

Corresponding authors

Ali H. Brivanlou (brvnlou@rockefeller.edu) and Charles M. Rice (ricec@rockefeller.edu).

Materials availability

The materials included in the current study are available from the corresponding authors on reasonable request.

Data and code availability

The datasets generated during and/or analyzed during the current study are available from the corresponding authors on reasonable request. RNA-seq data are available through the NCBI GEO accession numbers GSE163698 and GSE225564.

Maintenance of hESCs

RUES2 (NIHhESC-09-0013), RUES2-GLR (Figure S1) (Martyn et al., 2018), and HUES8 iCas9 (NIHhESC-09-0021; Figure S3) hESC lines were used in this study and maintained in HUESM (DMEM supplemented with 20% knockout serum replacement, 1×B27 supplement without vitamin A, 0.1 mM non-essential amino acids, 2 mM GlutaMax, and 0.1 mM 2-mercaptoethanol) conditioned by mouse embryonic fibroblasts (MEF-CM) and supplemented with 20 ng*mL^{-1} basic fibroblast growth factor. The cells were grown at 37°C and 5% CO2 on tissue culture dishes that were coated with Geltrex (Life Technologies) solution.

Generation of lung buds in micropatterns

hESCs were first differentiated into definitive endoderm using the STEMDiff Definitive Endoderm Kit (Stem Cell Technologies), with 1 day addition of supplement CJ and MR and 2 days addition of supplement CJ only. Upon endoderm induction, cells were washed once with PBS$^{-/-}$ (Gibco) and dissociated with Accutase (Stem Cell Technologies) for 7 min. Cells were then dissociated with a pipette to ensure a single-cell suspension and diluted four times in complete serum-free differentiation medium (cSFDM) containing DMEM/F12 (Gibco) with B27 Supplement with RA (Invitrogen, Waltham, MA), N2 Supplement (Invitrogen), 0.1% bovine serum albumin Fraction V (Invitrogen), β-mercaptoethanol (Sigma), Glutamax (ThermoFisher), 50 μg*mL^{-1} ascorbic acid (Sigma), and normocin with supplements of 10 μm SB431542 ("SB"; Tocris), 2 μm Dorsomorphin ("DS"; Stemgent) and 10 μm ROCK inhibitor (Y-27632; Abcam). Cells were further diluted with the same medium and 5 × 10^5 cells in 3.0 mL of medium were placed over a laminin-coated micropattern glass coverslips (CYTOOCHIPS Arena A, Arena 500A, Arena EMB A, Arena 225A) in a 35-mm tissue culture dish, left untouched for 10 min and then incubated at 37° C. After 3 h, the micropattern was washed once with PBS$^{+/+}$, which was then replaced with cSFDM with 10 μm SB and 2 μm DM. Two days later, the media were replaced with cSFDM with 10 μm SB and 2 μm DM. The next day, micropattern cultures were fed in lung induction medium (LIM), which contains cSFDM supplemented with 50 ng*mL^{-1} KGF (or indicated in each experiment; R&D systems), 10 ng*mL^{-1} BMP4 (R&D systems), 100 nM retinoic acid ("RA"; Sigma-Aldrich), and 3 μM CHIR9902 (EMD Millipore). Micropattern cultures were fed every other day in LIM for 7 days or until tissues were collected for analysis.

To generate lung buds on 96-well plates, 4 × 10^4 definitive endoderm cells were seeded on laminin-coated 96-well plates (CYTOOPlates, 200A), left untouched for 1 h, and then incubated at 37° C for 3 h in cSFDM with 10 μm SB, 2 μm DM, and 10 μm ROCK inhibitor. After this point, the protocol described above to generate lung buds on coverslips was followed.

Imaging

All confocal images were acquired on a Zeiss Inverted LSM 780 laser scanning confocal microscope with a ×10, ×20, or ×25 oil-immersion objective; 96-well plates were imaged on an ImageXpress Micro with a ×10 objective. Three-dimensional visualization and image processing was performed in ImageJ.

Imaging analysis

Analysis of confocal Z-stacks was performed using CellProfiler v4.0.4 (https://cellprofiler.org/). DAPI staining was used to identify nuclei and cytoplasm. Cells were classified as positive for a given marker based on the identification of fluorescence signals in either nuclear (for nuclear markers such as SOX9 and SOX2) or cytoplasmic area (SARS-CoV-2 and aCASP3) to estimate the percentage of marker-positive across Z-stacks.

Ninety-six-well plates image analysis was performed using ImageJ. Briefly, nuclei were identified and set to a mask. The regions of interest were then expanded, creating a new mask to interrogate cytoplasmic signals of virus infection in the SARS-CoV-2 images.

SARS-CoV-2 infection and transmission analysis

SARS-CoV-2 (strain: USA-WA1/2020) and HCoV-NL63 were obtained from BEI Resources (NR-52281 and NR-470). HCoV-OC43 was obtained from ZeptoMetrix (cat. #0810024CF) and HCoV-229E was generously provided by Volker Thiel (University of Bern). All viruses were amplified at 33°C in Huh-7.5 cells to generate a P1 stock. To generate working stocks, Huh-7.5 cells were infected at a multiplicity of infection (MOI) of 0.01 plaque-forming units (PFU)/cell (SARS-CoV-2, HCoV-NL63, HCoV-OC43) and 0.1 PFU/cell (HCoV-229E) and incubated at 33°C until virus-induced CPE was observed. Supernatants were subsequently harvested, clarified by centrifugation (3,000 × g × 10 min) at 4 dpi (HCoV-229E), 6 dpi (SARS-CoV-2, HCoV-OC43), and 10 dpi (HCoV-NL63), and aliquots stored at −80°C.

Viral titers were measured on Huh-7.5 cells by standard plaque assay. Briefly, 500 μL of serial 10-fold virus dilutions in Opti-MEM were used to infect 4 × 10^5 cells seeded the day prior into wells of a six-well plate. After 90-min adsorption, the virus inoculum was removed, and cells were overlaid with DMEM containing 10% FBS with 1.2% microcrystalline cellulose (Avicel). Cells were incubated for 4 days (HCoV-229E), 5 days (SARS-CoV-2, HCoV-OC43), and 6 days (HCoV-NL63) at 33°C, followed by fixation with 7% formaldehyde and crystal violet staining for plaque enumeration. All SARS-CoV-2 experiments were performed in a biosafety level 3 laboratory.

SARS-CoV-2 pseudotyped viruses were generated as described before using a C-terminally truncated SARS-CoV-2 S protein co-transfected with pNL4-3ΔEnv-nanoluc reporter (Robbiani et al., 2020). Infection by SARS-CoV pseudotyped viruses was quantified 48 h post-infection by measuring Nanoluc Luciferase activity in lysates using the Nano-Glo Luciferase Assay System (Promega) with Synergy *Neo*2 Multi-mode Microplate Reader (BioTek).

Antibody treatments

Neutralization assays were performed as previously described (Robbiani et al., 2020). Briefly, antibodies were serially diluted in LIM, mixed with a constant amount of SARS-CoV-2 (2 × 10^5 PFU for the larger chips, and 5 × 10^4 PFU in the 96-well plate assays) and incubated for 60 min at 37°C. The antibody-virus mix was then added to the 96-well plates or microchip containing human lung buds.

Statistical analysis

Statistical analysis was performed using unpaired two-sided t test and one-way ANOVA multiple comparison tests, unless stated otherwise. For all the experiments included in this study, three or more independent experiments were included using stage-matched controls as a reference. No statistical analysis was used to predetermine sample size and no data were excluded.

Detailed experimental procedures for immunostaining, scRNA-seq, and bulk RNA-seq analysis are included in the supplemental experimental procedures.

SUPPLEMENTAL INFORMATION

Supplemental information can be found online at https://doi.org/10.1016/j.stemcr.2023.03.015.

AUTHOR CONTRIBUTIONS

E.A.R. and A.H.B. conceived and designed the lung bud platform. E.A.R., B.R., H.H., C.M.R., and A.H.B. designed experiments with SARS-CoV-2. E.A.R. and B.R. executed and analyzed the experiments. E.A.R. and R.D.S. performed the single-cell gene expression analysis. Z.S. contributed to imaging and data analysis. J.L.P., D.B., T.S.C., and J.P. performed bioinformatic analysis of bulk RNA-seq data. E.A.R., B.R., and J.T.P. contributed to the identification and genetic analysis of BMP signaling in infected lung tissue. E.A.R., B.R., C.M.R., and A.H.B. wrote the manuscript with input from all authors.

ACKNOWLEDGMENTS

We thank C. Zhao and the Genomics Resource Center at the Rockefeller University for help and advice regarding single-cell RNA sequencing as well as A. North and the Bio-Imaging Resource Center for advice regarding imaging analysis. We thank M.R. Menezes and the High Throughput and Spectroscopy Center for critical advice in high-throughput model development and optimization. We thank members of the Brivanlou and Rice laboratories for critical discussions and comments on the manuscript. We thank F. Etoc, T. Haremaki, and Rumi Scientific Inc. for critical discussions and sharing reagents. This work was supported by the Pershing Square Foundation; NIH grants P01AI138398-S1, 2U19AI111825, and R01AI091707-10S1; a George Mason University Fast Grant; the BAWD Foundation; the G. Harold and Leila Y. Mathers Charitable Foundation; and private funding from the Rockefeller University.

CONFLICT OF INTERESTS

A.H.B. is a co-founder of startup companies RUMI Viro Inc., RUMI Scientific Inc., and OvaNova Laboratories, LLC, and serves on their scientific advisory boards. Both A.H.B. and E.A.R. are shareholders of RUMI Viro Inc. and RUMI Scientific Inc. C.M.R. is a founder of Apath LLC; a Scientific Advisory Board member of Imvaq Therapeutics, Vir Biotechnology, and Arbutus Biopharma; and an advisor for Regulus Therapeutics and Pfizer.

Received: August 24, 2022
Revised: March 23, 2023
Accepted: March 24, 2023
Published: April 20, 2023

REFERENCES

Blanco-Melo, D., Nilsson-Payant, B.E., Liu, W.C., Uhl, S., Hoagland, D., Møller, R., Jordan, T.X., Oishi, K., Panis, M., Sachs, D., et al. (2020). Imbalanced host response to SARS-CoV-2 drives development of COVID-19. Cell *181*, 1036–1045.e9.

Cassandras, M., Wang, C., Kathiriya, J., Tsukui, T., Matatia, P., Matthay, M., Wolters, P., Molofsky, A., Sheppard, D., Chapman, H., and Peng, T. (2020). Gli1+ mesenchymal stromal cells form a pathological niche to promote airway progenitor metaplasia in the fibrotic lung. Nat. Cell Biol. *22*, 1295–1306.

Chen, Y.W., Huang, S.X., de Carvalho, A.L.R.T., Ho, S.H., Islam, M.N., Volpi, S., Notarangelo, L.D., Ciancanelli, M., Casanova, J.L., Bhattacharya, J., et al. (2017). A three-dimensional model of human lung development and disease from pluripotent stem cells. Nat. Cell Biol. *19*, 542–549.

Danopoulos, S., Alonso, I., Thornton, M.E., Grubbs, B.H., Bellusci, S., Warburton, D., and Al Alam, D. (2018). Human lung branching morphogenesis is orchestrated by the spatiotemporal distribution of ACTA2, SOX2, and SOX9. Am. J. Physiol. Lung Cell Mol. Physiol. *314*, L144–L149.

Danopoulos, S., Bhattacharya, S., Mariani, T.J., and Al Alam, D. (2020). Transcriptional characterisation of human lung cells identifies novel mesenchymal lineage markers. Eur. Respir. J. *55*, 1900746.

Dye, B.R., Hill, D.R., Ferguson, M.A.H., Tsai, Y.H., Nagy, M.S., Dyal, R., Wells, J.M., Mayhew, C.N., Nattiv, R., Klein, O.D., et al. (2015). In vitro generation of human pluripotent stem cell derived lung organoids. Elife *4*, e05098.

Goo, L., Debbink, K., Kose, N., Sapparapu, G., Doyle, M.P., Wessel, A.W., Richner, J.M., Burgomaster, K.E., Larman, B.C., Dowd, K.A., et al. (2019). A protective human monoclonal antibody targeting the West Nile virus E protein preferentially recognizes mature virions. Nat. Microbiol. *4*, 71–77.

Green, M.D., Chen, A., Nostro, M.C., d'Souza, S.L., Schaniel, C., Lemischka, I.R., Gouon-Evans, V., Keller, G., and Snoeck, H.W. (2011). Generation of anterior foregut endoderm from human embryonic and induced pluripotent stem cells. Nat. Biotechnol. *29*, 267–272.

Han, Y., Duan, X., Yang, L., Nilsson-Payant, B.E., Wang, P., Duan, F., Tang, X., Yaron, T.M., Zhang, T., Uhl, S., et al. (2020). Identification of SARS-CoV-2 inhibitors using lung and colonic organoids. Nature *589*, 270–275. https://doi.org/10.1038/s41586-020-2901-9.

Haremaki, T., Metzger, J.J., Rito, T., Ozair, M.Z., Etoc, F., and Brivanlou, A.H. (2019). Self-organizing neuruloids model developmental aspects of Huntington's disease in the ectodermal compartment. Nat. Biotechnol. *37*, 1198–1208.

Hoffmann, H.H., Sánchez-Rivera, F.J., Schneider, W.M., Luna, J.M., Soto-Feliciano, Y.M., Ashbrook, A.W., Le Pen, J., Leal, A.A., Ricardo-Lax, I., Michailidis, E., et al. (2021). Functional interrogation of a SARS-CoV-2 host protein interactome identifies unique and shared coronavirus host factors. Cell Host Microbe *29*, 267–280.e5. https://doi.org/10.1016/j.chom.2020.12.009.

Hou, Y.J., Okuda, K., Edwards, C.E., Martinez, D.R., Asakura, T., Dinnon, K.H., 3rd, Kato, T., Lee, R.E., Yount, B.L., Mascenik, T.M., et al. (2020). SARS-CoV-2 reverse genetics reveals a variable infection gradient in the respiratory tract. Cell *182*, 429–446.e14.

Huang, J., Hume, A.J., Abo, K.M., Werder, R.B., Villacorta-Martin, C., Alysandratos, K.D., Beermann, M.L., Simone-Roach, C., Lindstrom-Vautrin, J., Olejnik, J., et al. (2020). SARS-CoV-2 infection of pluripotent stem cell-derived human lung alveolar type 2 cells elicits a rapid epithelial-intrinsic inflammatory response. Cell Stem Cell *27*, 962–973.e7. https://doi.org/10.1016/j.stem.2020.09.013.

Huang, S.X.L., Green, M.D., de Carvalho, A.T., Mumau, M., Chen, Y.W., D'Souza, S.L., and Snoeck, H.W. (2015). Protocol of for the

generation of lung and airway epithelial cells from human pluripotent stem cells. Nat. Protoc. *10*, 413–425.

Huang, S.X.L., Islam, M.N., O'Neill, J., Hu, Z., Yang, Y.G., Chen, Y.W., Mumau, M., Green, M.D., Vunjak-Novakovic, G., Bhattacharya, J., and Snoeck, H.W. (2014). Highly efficient generation of lung and airway epithelial cells from human pluripotent stem cells. Nat. Biotechnol. *32*, 84–91.

Jacob, A., Morley, M., Hawkins, F., McCauley, K.B., Jean, J.C., Heins, H., Na, C.L., Weaver, T.E., Vedaie, M., Hurley, K., et al. (2017). Differentiation of human pluripotent stem cells into functional lung alveolar epithelial cells. Cell Stem Cell *21*, 472–488.e10.

Katsura, H., Sontake, V., Tata, A., Kobayashi, Y., Edwards, C.E., Heaton, B.E., Konkimalla, A., Asakura, T., Mikami, Y., Fritch, E.J., et al. (2020). Human lung stem cell-based alveolospheres provide insights into SARS-CoV-2-mediated interferon responses and pneumocyte dysfunction. Cell Stem Cell *27*, 890–904.e8. https://doi.org/10.1016/j.stem.2020.10.005.

Lamers, M.M., van der Vaart, J., Knoops, K., Riesebosch, S., Breugem, T.I., Mykytyn, A.Z., Beumer, J., Schipper, D., Bezstarosti, K., Koopman, C.D., et al. (2021). An organoid-derived bronchioalveolar model for SARS-CoV-2 infection of human alveolar type II-like cells. EMBO J. *40*, e105912. https://doi.org/10.15252/embj.2020105912.

Martyn, I., Kanno, T.Y., Ruzo, A., Siggia, E.D., and Brivanlou, A.H. (2018). Self-organization of a human organizer by combined Wnt and nodal signalling. Nature *558*, 132–135.

McCauley, K.B., Hawkins, F., Serra, M., Thomas, D.C., Jacob, A., and Kotton, D.N. (2017). Efficient derivation of functional human airway epithelium from pluripotent stem cells via temporal regulation of Wnt signaling. Cell Stem Cell *20*, 844–857.e6.

Miller, A.J., Dye, B.R., Ferrer-Torres, D., Hill, D.R., Overeem, A.W., Shea, L.D., and Spence, J.R. (2019). Generation of lung organoids from human pluripotent stem cells in vitro. Nat. Protoc. *14*, 518–540.

Morrisey, E.E., and Hogan, B.L.M. (2010). Preparing for the first breath: genetic and cellular mechanisms in lung development. Dev. Cell *18*, 8–23.

Muus, C., Luecken, M.D., Eraslan, G., Waghray, A., Heimberg, G., Sikkema, L., Kobayashi, Y., Vaishnav, E.D., Subramanian, A., Smilie, C., et al. (2020). Integrated analyses of single-cell atlases reveal age, gender, and smoking status associations with cell type-specific expression of mediators of SARS-CoV-2 viral entry and highlights inflammatory programs in putative target cells. Preprint at bioRxiv. https://doi.org/10.1101/2020.04.19.049254.

Nikolić, M.Z., Caritg, O., Jeng, Q., Johnson, J.A., Sun, D., Howell, K.J., Brady, J.L., Laresgoiti, U., Allen, G., Butler, R., et al. (2017). Human embryonic lung epithelial tips are multipotent progenitors that can be expanded in vitro as long-term self-renewing organoids. Elife *6*, e26575.

Nikolić, M.Z., Sun, D., and Rawlins, E.L. (2018). Human lung development: recent progress and new challenges. Development *145*, dev163485.

Park, J., Foox, J., Hether, T., Danko, D.C., Warren, S., Kim, Y., Reeves, J., Butler, D.J., Mozsary, C., Rosiene, J., et al. (2022). System-wide transcriptome damage and tissue identity loss in COVID-19 patients. Cell Rep. Med. *3*, 100522.

Rawlins, E.L., Clark, C.P., Xue, Y., and Hogan, B.L.M. (2009). The Id2+ distal tip lung epithelium contains individual multipotent embryonic progenitor cells. Development *136*, 3741–3745.

Ren, Y., Shu, T., Wu, D., Mu, J., Wang, C., Huang, M., Han, Y., Zhang, X.Y., Zhou, W., Qiu, Y., and Zhou, X. (2020). The ORF3a protein of SARS-CoV-2 induces apoptosis in cells. Cell. Mol. Immunol. *17*, 881–883. https://doi.org/10.1038/s41423-020-0485-9.

Robbiani, D.F., Gaebler, C., Muecksch, F., Lorenzi, J.C.C., Wang, Z., Cho, A., Agudelo, M., Barnes, C.O., Gazumyan, A., Finkin, S., et al. (2020). Convergent antibody responses to SARS-CoV-2 in convalescent individuals. Nature *584*, 437–442.

Salahudeen, A.A., Choi, S.S., Rustagi, A., Zhu, J., van Unen, V., de la O, S.M., Flynn, R.A., Margalef-Català, M., Santos, A.J.M., Ju, J., et al. (2020). Progenitor identification and SARS-CoV-2 infection in human distal lung organoids. Nature *588*, 670–675. https://doi.org/10.1038/s41586-020-3014-1.

Schneider, W.M., Luna, J.M., Hoffmann, H.H., Sánchez-Rivera, F.J., Leal, A.A., Ashbrook, A.W., Le Pen, J., Ricardo-Lax, I., Michailidis, E., Peace, A., et al. (2021). Genome-scale identification of SARS-CoV-2 and pan-coronavirus host factor networks. Cell *184*, 120–132.e14. https://doi.org/10.1016/j.cell.2020.12.006.

Shang, J., Wan, Y., Luo, C., Ye, G., Geng, Q., Auerbach, A., and Li, F. (2020). Cell entry mechanisms of SARS-CoV-2. Proc. Natl. Acad. Sci. USA *117*, 11727–11734.

Stuart, T., Butler, A., Hoffman, P., Hafemeister, C., Papalexi, E., Mauck, W.M., 3rd, Hao, Y., Stoeckius, M., Smibert, P., and Satija, R. (2019). Comprehensive integration of single-cell data. Cell *177*, 1888–1902.e21.

Sungnak, W., Huang, N., Bécavin, C., Berg, M., Queen, R., Litvinukova, M., Talavera-López, C., Maatz, H., Reichart, D., Sampaziotis, F., et al. (2020). SARS-CoV-2 entry factors are highly expressed in nasal epithelial cells together with innate immune genes. Nat. Med. *26*, 681–687.

Tindle, C., Fuller, M., Fonseca, A., Taheri, S., Ibeawuchi, S.R., Beutler, N., Katkar, G.D., Claire, A., Castillo, V., Hernandez, M., et al. (2021). Adult stem cell-derived complete lung organoid models emulate lung disease in COVID-19. Elife *10*, e66417. https://doi.org/10.7554/eLife.66417.

Travaglini, K.J., Nabhan, A.N., Penland, L., Sinha, R., Gillich, A., Sit, R.V., Chang, S., Conley, S.D., Mori, Y., Seita, J., et al. (2020). A molecular cell atlas of the human lung from single-cell RNA sequencing. Nature *587*, 619–625.

Walls, A.C., Park, Y.J., Tortorici, M.A., Wall, A., McGuire, A.T., and Veesler, D. (2020). Structure, function, and antigenicity of the SARS-CoV-2 spike glycoprotein. Cell *181*, 281–292.e6.

Wang, A., Chiou, J., Poirion, O.B., Buchanan, J., Valdez, M.J., Verheyden, J.M., Hou, X., Kudtarkar, P., Narendra, S., Newsome, J.M., et al. (2020). Single-cell multiomic profiling of human lungs reveals cell-type-specific and age-dynamic control of SARS-CoV2 host genes. Elife *9*, e62522.

Warmflash, A., Sorre, B., Etoc, F., Siggia, E.D., and Brivanlou, A.H. (2014). A method to recapitulate early embryonic spatial

patterning in human embryonic stem cells. Nat. Methods *11*, 847–854.

Williamson, E.J., Walker, A.J., Bhaskaran, K., Bacon, S., Bates, C., Morton, C.E., Curtis, H.J., Mehrkar, A., Evans, D., Inglesby, P., et al. (2020). Factors associated with COVID-19-related death using OpenSAFELY. Nature *584*, 430–436.

World Health Organization (2023). WHO Coronavirus (COVID-19) Dashboard.

Wu, X., Dao Thi, V.L., Huang, Y., Billerbeck, E., Saha, D., Hoffmann, H.H., Wang, Y., Silva, L.A.V., Sarbanes, S., Sun, T., et al. (2018). Intrinsic immunity shapes viral resistance of stem cells. Cell *172*, 423–438.e25.

Yano, T., Mason, R.J., Pan, T., Deterding, R.R., Nielsen, L.D., and Shannon, J.M. (2000). KGF regulates pulmonary epithelial proliferation and surfactant protein gene expression in adult rat lung. Am. J. Physiol. Lung Cell Mol. Physiol. *279*, L1146–L1158.

Youk, J., Kim, T., Evans, K.V., Jeong, Y.I., Hur, Y., Hong, S.P., Kim, J.H., Yi, K., Kim, S.Y., Na, K.J., et al. (2020). Three-dimensional human alveolar stem cell culture models reveal infection response to SARS-CoV-2. Cell Stem Cell *27*, 905–919.e10. https://doi.org/10.1016/j.stem.2020.10.004.

Zepp, J.A., and Morrisey, E.E. (2019). Cellular crosstalk in the development and regeneration of the respiratory system. Nat. Rev. *20*, 551–566.

Zhang, Q., Bastard, P., Liu, Z., Le Pen, J., Moncada-Velez, M., Chen, J., Ogishi, M., Sabli, I.K.D., Hodeib, S., Korol, C., et al. (2020). Inborn errors of type I IFN immunity in patients with life-threatening COVID-19. Science *370*, eabd4570.

Zhou, P., Yang, X.L., Wang, X.G., Hu, B., Zhang, L., Zhang, W., Si, H.R., Zhu, Y., Li, B., Huang, C.L., et al. (2020). A pneumonia outbreak associated with a new coronavirus of probable bat origin. Nature *579*, 270–273.

Zhu, N., Zhang, D., Wang, W., Li, X., Yang, B., Song, J., Zhao, X., Huang, B., Shi, W., Lu, R., et al. (2020). A novel coronavirus from patients with pneumonia in China. NEJM *382*, 727–733.

Ziegler, C.G.K., Allon, S.J., Nyquist, S.K., Mbano, I.M., Miao, V.N., Tzouanas, C.N., Cao, Y., Yousif, A.S., Bals, J., Hauser, B.M., et al. (2020). SARS-CoV-2 receptor ACE2 is an interferon-stimulated gene in human airway epithelial cells and is detected in specific cell subsets across tissues. Cell *181*, 1016–1035.e19.

Stem Cell Reports, Volume *18*

Supplemental Information

Organotypic human lung bud microarrays identify BMP-dependent SARS-CoV-2 infection in lung cells

E.A. Rosado-Olivieri, B. Razooky, J. Le Pen, R. De Santis, D. Barrows, Z. Sabry, H.-H. Hoffmann, J. Park, T.S. Carroll, J.T. Poirier, C.M. Rice, and A.H. Brivanlou

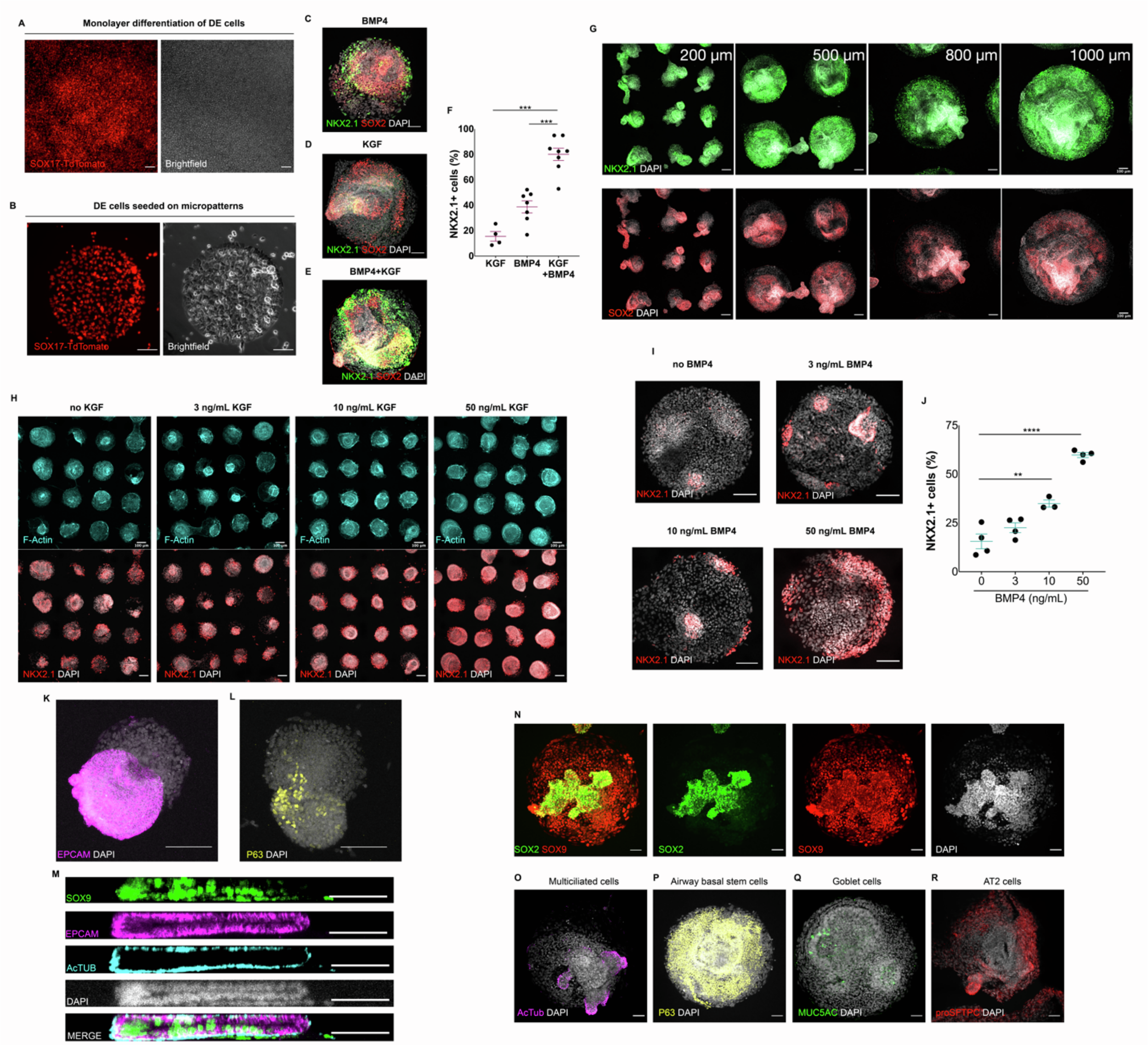

Supplementary Figure 1: Induction of NKX2.1+ lung progenitors on confined geometries, related to Figure 1. A) Monolayer differentiation of SOX17+ endoderm progenitors pre-seeding. B) SOX17+ endoderm progenitors on confined geometry 3 hours post-seeding. C-F) Induction of NKX2.1+ multipotent lung and SOX2+ airway progenitors upon modulation of BMP4, KGF or KGF+BMP4. (scale bar: 50 µm). Experiments in A-E were performed using the RUES2-GLR cell lines (N=4 independent experiments). G) Efficient induction of NKX2.1+ multipotent lung and SOX2+ airway progenitors in colonies of varying sizes. (scale bar: 100 µm). H) Low magnification images of epithelial buds containing NKX2.1+ lung progenitors grown on confined geometries of 225 µm diameter at varying doses of KGF. Epithelial structures can be identified with F-actin staining. I-J) Induction of NKX2.1+ lung progenitors on confined geometries of 500 µm diameter at varying doses of BMP4. K-M) Top and side-view of lung buds expressing EPCAM (K), P63 (L), SOX9 (M) and AcTub (M) along proximo-distal axis. N-R) Identification of SOX2+ and SOX9+ progenitors as well as AcTub+ multiciliated cells (O), P63+ airway basal stem cells (P), MUC5AC+ goblet cells (Q) and proSFTPC+ type 2 pneumocytes (R) in 500 µm colonies. (scale bar: 50µm)

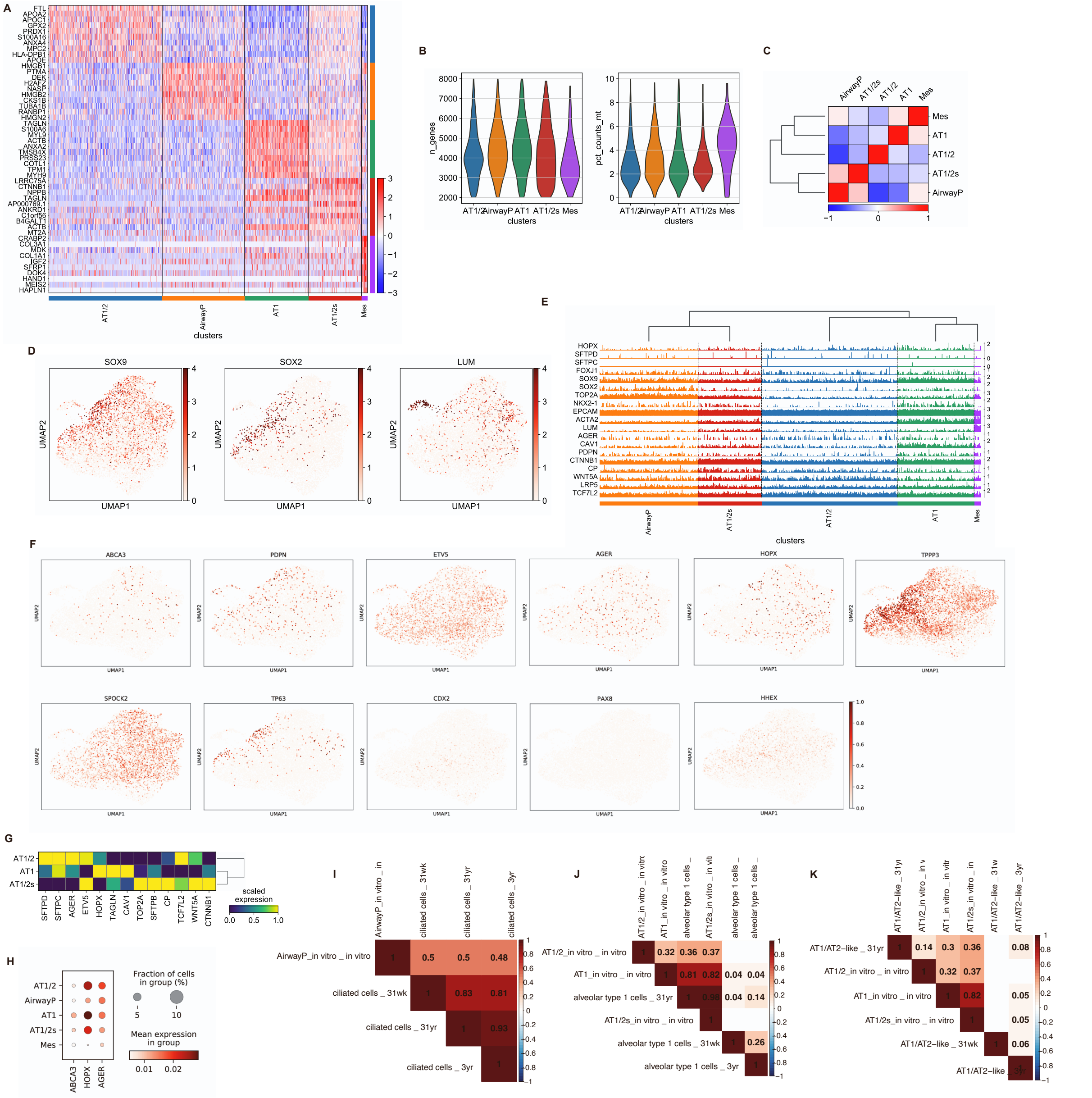

Supplementary Figure 2: Single-cell gene expression analysis of lung buds, related to Figure 2. A) Heatmap of top 10 differentially expressed genes for each cluster identified in synthetic lung buds. z-score normalized expression values are shown. B) Violin plots of the number of genes (n_genes) and percentage of mitochondrial genes (pct_counts_mt) for each cluster identified in synthetic lung buds. C) Cluster-level gene expression Pearson correlation analysis of clusters identified in synthetic lung buds. z-score normalized correlation values are shown. D) UMAP expression plots of SOX9, SOX2 and lumican (LUM). E) Gene expression trackplots of cell type-specific markers. Each peak represents a single cell and its height denotes the expression level of each gene. F) UMAP expression plots of alveolar, airway, hepatic, thymic and intestinal markers. G-H) Heatmap and dotplot of scaled gene expression levels of alveolar markers. I-K) Cluster-level stage-dependent gene expression Pearson's correlation analysis of airway (I) and alveolar cell types (J-K) identified in *in vitro*-derived lung buds and in adult lung tissue at different developmental stages.

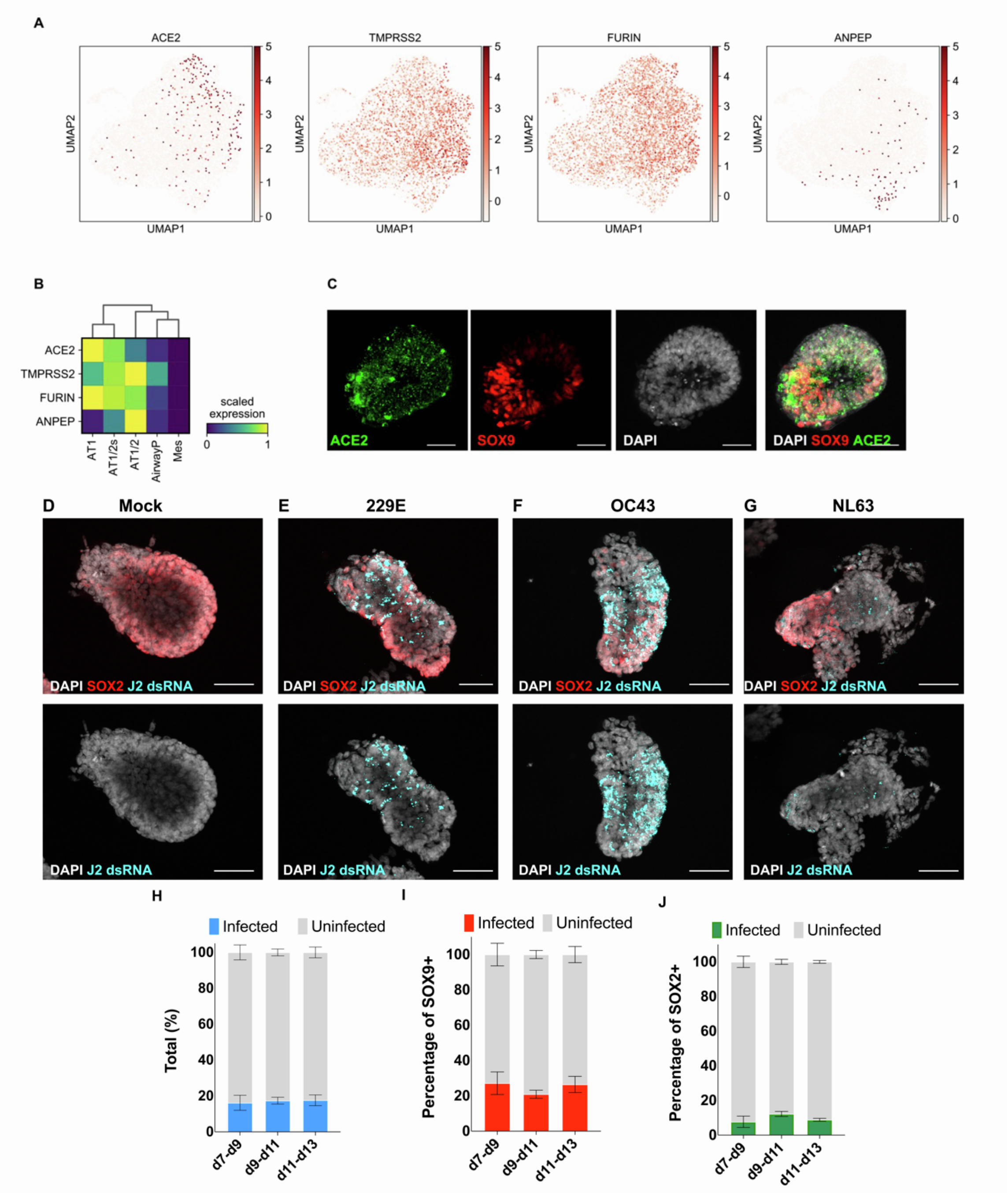

Supplementary Figure 3: Expression of coronaviruses-associated genes and infection by endemic coronaviruses, related to Figure 3. A) UMAP expression plots of ACE2, TMPRSS2, FURIN and ANPEP. B) Scaled expression of SARS-CoV-2 entry factors for each of the identified clusters in synthetic human lung buds. C) Expression of the ACE2 receptor in *in vitro*-derived lung buds. D-G) Synthetic lung buds infected with endemic coronaviruses HCoV-229E (E), HCoV-OC43 (F) and HCoV-NL63 (G). Infected cells were identified by staining with J2 antibody detecting dsRNA. H-J) Percentage of total (H), SOX9+ (I) and SOX2+ (J) cells infected by SARS-CoV-2 at multiple stages of lung bud formation (N=9 independent experiments). (scale bar: 50µm)

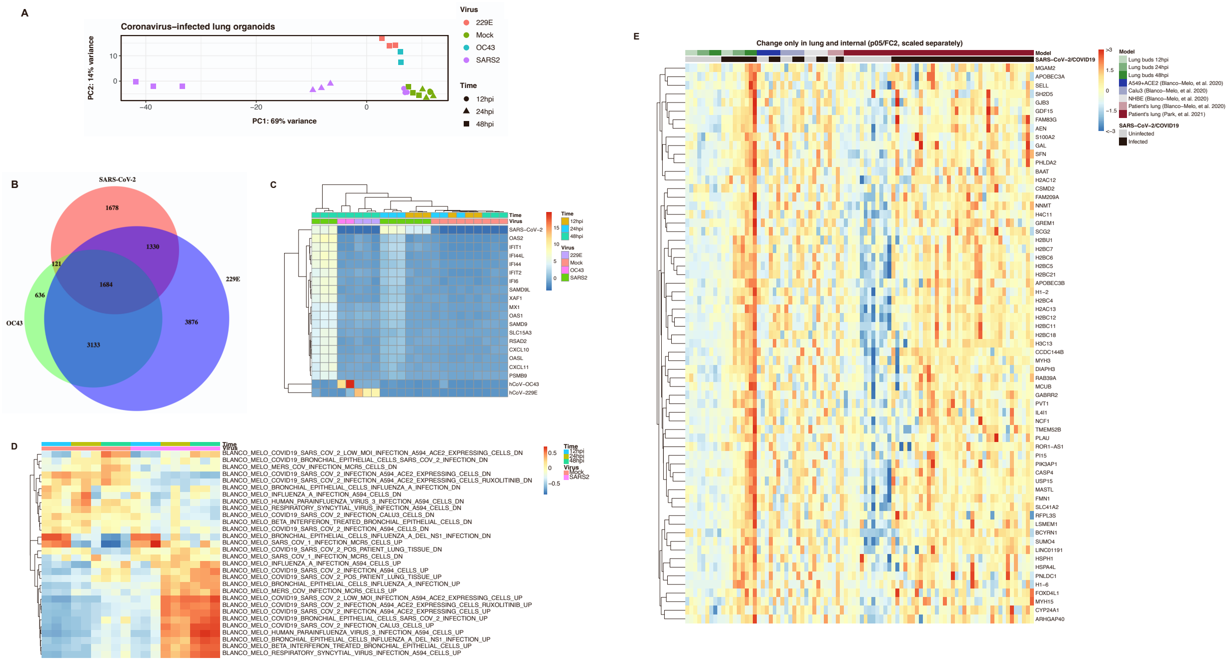

Supplementary Figure 4: Gene expression analysis of infected synthetic lung buds, related to Figure 6. A) PCA analysis of gene expression of infected and mock synthetic lung buds 12, 24, and 48 hpi. B) Venn diagram of DEG in synthetic lung buds collected 48 hpi after infection by SARS-CoV-2, OC43 or 229E. C) Heatmap of the expression of top 20 SARS-CoV-2-associated DEG. D) Heatmap of enrichment scores of COVID-19-associated gene sets in each sample from Blanco-Melo et al., 2020[38]. E) Heatmap of genes identified to be up-regulated in SARS-CoV-2-infected synthetic lung buds and postmortem lung tissue but not cancer cell lines.

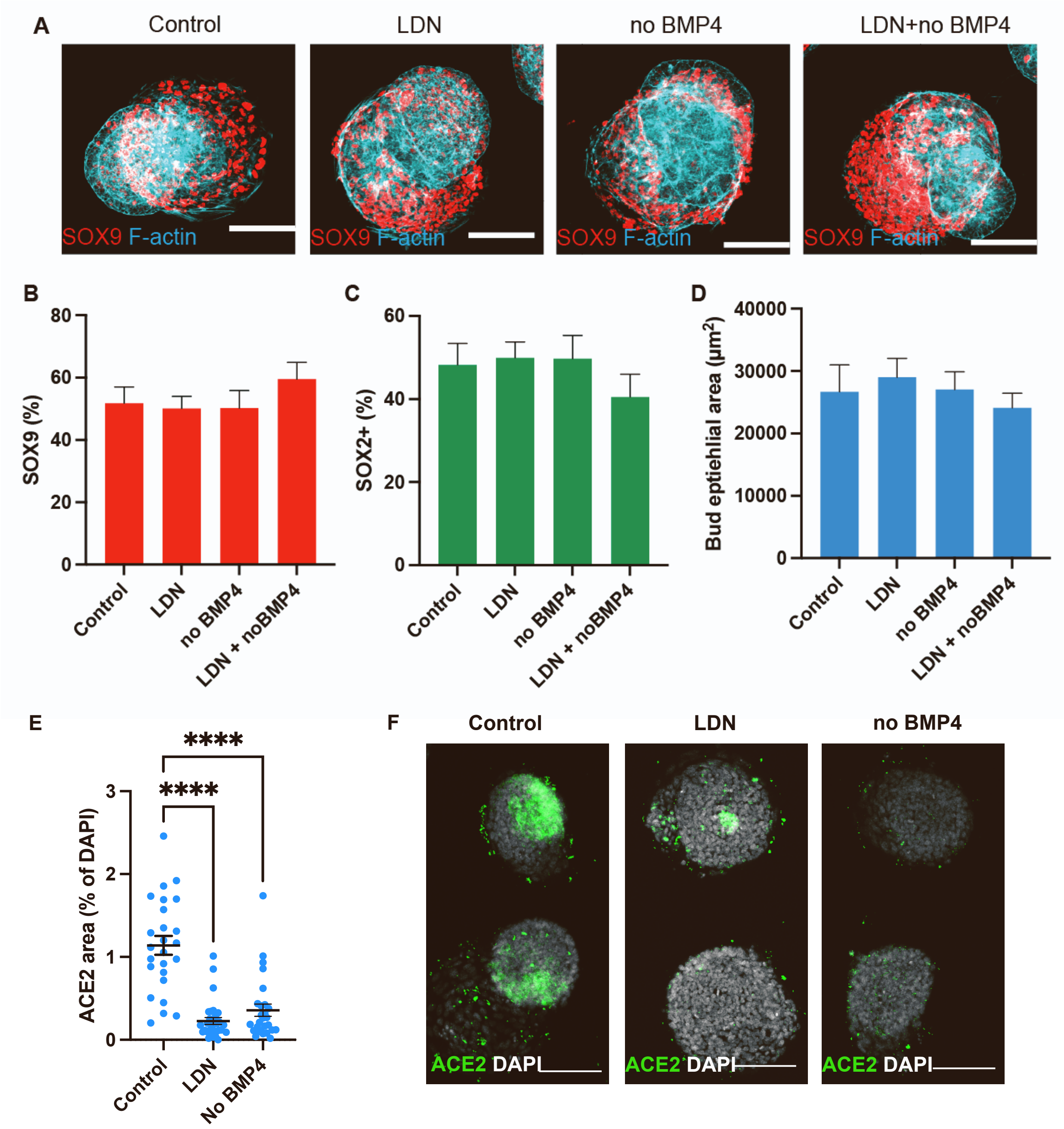

Supplementary Figure 5: Effect of BMP inhibition on lung buds and ACE2 expression. A) Expression of SOX9 and F-actin in lung buds treated with LDN and/or no BMP4 (scale bar: 50µm). B-C) Quantification of SOX9+ alveolar and SOX2+ airway cells. D) Quantification of epithelial area. E-F) Expression of ACE2 in lung buds upon BMP inhibition or removal. (N=3 independent experiments) (scale bar: 100µm)

Supplementary Experimental Procedures

Immunostaining

Micropattern coverslips were fixed with 4% paraformaldehyde (Electron Microscopy Sciences 15713) in warm medium for 30 min, rinsed three times with $PBS^{-/-}$, and then blocked and permeabilized with 3% normal donkey serum (Jackson Immunoresearch 017-000-121) with 0.5% Triton X-100 (Sigma 93443) in $PBS^{-/-}$ for 30 min. Micropatterns were incubated with primary antibodies for 1.5 h, washed three times in $PBS^{-/-}$ for 5 min each, incubated with secondary antibodies conjugated with Alexa 488, Alexa 555, Alexa 594 or Alexa 647 (1:1,000 dilution, Molecular Probes), fluorescently-conjugated phalloidin (1:400; Life Technologies) and 10 $ng*ml^{-1}$ of DAPI (Thermo Fisher Scientific D1306) for 30 min and then washed three times with $PBS^{-/-}$. Coverslips were mounted on slides using ProLong Gold antifade mounting medium (Molecular Probes P36934).

The primary antibodies used were as follows: rabbit anti-SOX9 (Millipore; AB5535; 1:250), goat anti-SOX2 (R&D Systems; AF2018; 1:250), rabbit anti-NKX2.1 (Abcam; ab76013; 1:200), mouse anti-Acetylated Tubulin (Sigma; T7451; 1:1000), goat anti-TP63 (R&D Systems; BAF1916; 1:250), rabbit anti-proSPC (Seven Hills; WRAB-9337; 1:500), mouse anti-HOPX (Santa Cruz; sc-398703; 1:250), rabbit anti-nucleocapsid SARS-CoV-2 (GeneTex; GTX135357; 1;1000); rabbit anti-Active Caspase-3 (R&D Systems; AF835; 1:250); mouse anti-Mucin 5AC (Abcam; ab3649; 1:250); human anti-Spike SARS-CoV-2 (1:1,000) (Robbianit et al., 2020); anti-HNF-3BETA/FOXA2 (Neuromics; GT15186; 1:200); mouse J2 dsRNA (SCICONS; 1;1,000), phospho Histone H3 (Cell Signaling; 9706S; 1:200), goat anti-SOX17 (R&D Systems; AF1924; 1:200), rabbit anti-pSMAD1/5 (Cell Signaling; 9516; 1:200) and goat anti-AGER (R&D Systems; AF1145; 1:200). To detect infected cells for HCoV-229E, HCoV-OC43 and HCoV-NL63, a mouse monoclonal anti-dsRNA antibody (Scicons: catalog no. 10010500) was used under similar conditions.

Single cell RNA-sequencing analysis

Micropatterned coverslips with 225-μm diameter synthetic lung buds at day 7 of lung induction were dissociated with TrypLE Express (Gibco) for 10 min at 37 °C. After dissociation, the cells

were washed three times in $PBS^{-/-}$ (Gibco) with 0.04% BSA and strained through a Flowmi tip 40 µm strainer. Cell count and viability were determined on a Countess II Automated Cell Counter. Samples were loaded for capture with the Chromium System using the Single Cell 3′ v3 reagents (10X Genomics). Following cell capture and lysis, cDNA was synthesized and amplified according to the manufacturer's instructions (10X Genomics). The resulting libraries were sequenced on the NovaSeq platform. The Cell Ranger (v.2.0.2) software pipeline was used to create FASTQ files which were aligned to the hg19 genome using default parameters. These data are available through the NCBI GEO accession number GSE163698.

Filtered gene expression matrices were generated using Cell Ranger and subsequently used for downstream analyses using Scanpy (v.1.6.0) (https://pypi.org/project/scanpy/). Data was filtered to have a minimum of 2000 and a maximum of 8000 detected genes per cell and genes were filtered to be expressed in at least 3 cells. Cells with over 10% mitochondrial genes were discarded. Clustering of cells was performed using the Leiden algorithm (resolution=0.4) and visualized using UMAP plots (n.neighbors = 50, PCA components= 50). Differential gene expression analysis was performed using the Wilcoxon rank-sum (Mann-Whitney-U) test to identify cluster markers.

To compute correlations with human lung tissue, we performed data integration of our dataset with a published single cell dataset of lung tissue from a 30wk, 3 yr and 30 yr old donors (Wang et al. 2020). We integrated these datasets using canonical correlation analysis (Seurat v4.0) (Stuart et al. 2019) with FindIntegrationAnchors() and IntegrateData() using the first 30 principal components. After integration, we estimated the average expression value for each gene in each cell type and computed Pearson's correlations for all possible pairs of cell types with rcorr(). Significant correlation values (adjusted p-value < 0.05) were plotted with corrplot(). Hierarchical clustering based on correlation coefficients was performed with hclust().

RNA-sequencing analysis

Synthetic lung buds were collected 12, 24 and 48 hpi for total RNA extraction. The quality of RNA samples was determined using an Agilent 2100 Bioanalyzer, and all samples for sequencing had

RNA integrity (RIN) numbers of more than 8. Poly(A) selection and library preparation using an Illumina TrueSeq mRNA sample preparation kit, and sequencing on a NovaSeq SP 2x50 platform.

Reads were trimmed and quality checked with Trim Galore! v0.6.6 powered by Cutadapt v1.18 (Kechin et al. 2017); command: trim_galore -q 20 --fastqc -e 0.1 --length 20 --paired --path_to_cutadapt cutadapt <read1> <read2>. Next, reads were mapped on a combined human genome (GRCh38.p13), HCoV-229E genome (GenBank: NC_002645.1) (Thiel et al. 2001), HCoV-OC43 genome (GenBank: NC_006213) (St-Jean et al. 2004), and SARS-CoV-2 genome (GenBank: MN985325.1) (Harcourt et al. 2020) with STAR v2.7.6a (Dobin et al. 2013); command: STAR --readFilesIn <read1> <read2> --outFileNamePrefix <prefix> --genomeDir <combined_genome> --readFilesCommand zcat --runThreadN 12 --chimScoreJunctionNonGTAG -1 --outSAMtype BAM SortedByCoordinate --chimOutType Junctions SeparateSAMold --alignSJDBoverhangMin 5 --outFilterMultimapScoreRange 1 --outFilterMultimapNmax 5 --outMultimapperOrder Random --outSAMattributes NH HI AS nM NM XS ch --chimSegmentMin 10 --chimJunctionOverhangMin 10 --chimScoreMin 1 --chimScoreDropMax 30 --chimScoreSeparation 7 --chimSegmentReadGapMax 3 --chimFilter None --twopassMode None --alignSJstitchMismatchNmax 5 -1 5 5 --chimMainSegmentMultNmax 10. Resulting bam files were tagged with read groups and merged by sample using Picard tools v2.23.6 (http://broadinstitute.github.io/picard/), and indexed with samtools v1.3.1 (Li et al. 2009). Genes were counted using features_count powered by Rsubread v2.2.6 in R v4.0.2 (Liao et al. 2019), using recommended parameters for stranded paired-end RNAseq and a combined human/HCoV-229E/HCoV-OC43/SARS-CoV-2 annotation GTF file. Estimation of variance–mean dependence from the count data and principal component analysis was performed with DESeq2 v1.28.1 (Love et al. 2014) using R v4.0.2, using the constructor function DESeqDataSetFromMatrix(design = ~virus + time). All the result tables were built using the DESeq2 results() function.

Sequence and transcript coordinates for mouse mm10 UCSC genome and gene models were retrieved from the BSgenome.Hsapiens.UCSC.hg38 Bioconductor package (version 1.4.1) and TxDb.Mmusculus.UCSC.hg38.knownGene (version 3.4.0) Bioconductor libraries, respectively. Transcript expressions were calculated using the Salmon quantification software (Patro et al. 2017)

and gene expression levels as TPMs and counts retrieved using Tximport[52] (version 1.8.0) (Love et al. 2015). Normalization and rlog transformation of raw read counts in genes were performed using DESeq2 (version 1.20.0) (Love et al. 2016). Gene set enrichment analysis (GSEA), over representation analysis, and visualization of the enriched gene ontology/pathway terms as network plots were performed with the clusterProfiler R Bioconductor package (version 3.18.1) (Yu et al. 2012, Subramanian et al. 2005). Gene set variation analysis (GSVA) was done on the RNAseq counts matrix (normalized to sequencing depth) using the GSVA R Bioconductor package (version 1.38.2) (Hänzelmann et al. 2013). Published COVID19 gene lists for the GSVA analysis were extracted from the Molecular Signatures Database (MSigDB) using the msigdbr R package (version 7.2.1). Heatmaps (for both GSVA and gene expression) were generated using the pheatmap R package (Kolde et al. 2019). These data are available through the NCBI GEO accession number GSE225564.

References

Dobin et al. (2013) STAR: ultrafast universal RNA-seq aligner. Bioinformatics 29(1):15-21.

Hänzelmann S, Castelo R, Guinney J (2013). "GSVA: gene set variation analysis for microarray and RNA-Seq data." BMC Bioinformatics, 14, 7. doi: 10.1186/1471-2105-14-7, http://www.biomedcentral.com/1471-2105/14/7.

Harcourt et al. (2020) Severe Acute Respiratory Syndrome Coronavirus 2 from Patient with Coronavirus Disease, United States. Emerg Infect Dis. 26(6):1266-1273.

Kechin et al. (2017) cutPrimers: A New Tool for Accurate Cutting of Primers from Reads of Targeted Next Generation Sequencing. J. Comput. Biol. 24(11):1138-1143.

Kolde, R. pheatmap: Pretty Heatmaps. R package version 1.0.12 (2019).

Li et al. (2009) The Sequence Alignment/Map format and SAMtools. Bioinformatics 25(16):2078-9.

Liao et al. (2019) The R package Rsubread is easier, faster, cheaper and better for alignment and quantification of RNA sequencing reads. Nucleic Acids Res 47(8):e47.

Love MI, Hogenesch J, Irizarry R. (2016) Modeling of RNA-seq fragment sequence bias reduces systematic errors in transcript abundance estimation. Nat Biotechnol. 34(12):1287-1291

Love, MI et al. (2014) Moderated estimation of fold change and dispersion for RNA-seq data with DESeq2. Genome Biol 15(12):550.

Patro R, Duggal G, Love MI, Irizarry RA, Kingsford C. (2017) Salmon provides fast and bias-aware quantification of transcript expression. Nat Methods. 14(4):417-419.

Robbiani et al. (2020) Convergent antibody responses to SARS-CoV-2 in convalescent individuals. Nature 584, 437–442.

St-Jean et al. (2004) Human respiratory coronavirus OC43: genetic stability and neuroinvasion. J Virol. 78(16):8824-34.

Subramanian, Tamayo, et al. Gene set enrichment analysis: A knowledge-based approach for interpreting genome-wide expression profiles 2005, PNAS 102, 15545-15550

Thiel et al. (2001) Infectious RNA transcribed in vitro from a cDNA copy of the human coronavirus genome cloned in vaccinia virus. J Gen Virol. 82, 1273-1281.

Yu G, Wang L, Han Y, He Q (2012). “clusterProfiler: an R package for comparing biological themes among gene clusters.” OMICS: A Journal of Integrative Biology, 16(5), 284-287.
